# Immunomodulatory Treatment of Multisystem Inflammatory Syndrome in Children

**DOI:** 10.1056/NEJMoa2102968

**Published:** 2021-06-16

**Authors:** Andrew J. McArdle, Ortensia Vito, Harsita Patel, Eleanor G. Seaby, Priyen Shah, Clare Wilson, Claire Broderick, Ruud Nijman, Adriana Tremoulet, Daniel Munblit, Rolando Ulloa-Gutierrez, Michael J Carter, Tisham De, Clive Hoggart, Elizabeth Whittaker, Jethro A. Herberg, Myrsini Kaforou, Aubrey J. Cunnington, Michael Levin

**Affiliations:** 1 Department of Infectious Disease, Section of Paediatric Infectious Disease, Imperial College London, London, United Kingdom; 2 Genomic Informatics Group, University of Southampton, Southampton, United Kingdom; 3 Translational Genomics Group, Broad Institute of MIT and Harvard, Cambridge, MA, USA; 4 Department of Paediatrics, Imperial College Healthcare NHS Trust, London, United Kingdom; 5 Kawasaki Disease Research Center, Department of Pediatrics, University of California San Diego; 6 Department of Pediatrics and Pediatric Infectious Diseases, Institute of Child's Health, Sechenov First Moscow State Medical University (Sechenov University), Moscow, Russia; 7 Inflammation, Repair, and Development Section, National Heart and Lung Institute, Faculty of Medicine, Imperial College London, London, United Kingdom; 8 Hospital Nacional de Niños “Dr. Carlos Sáenz Herrera”, Centro de Ciencias Médicas, Caja Costarricense de Seguro Social (C.C.S.S.), San José, Costa Rica; 9 Department of Women and Children's Health, School of Life Course Sciences, King's College London, St Thomas' Hospital, SE1 7EH, London, UK; 10 Department of Genetics and Genomic Sciences, Icahn School of Medicine at Mount Sinai, New York City, NY USA

## Abstract

**Background:**

Evidence is urgently needed to support treatment decisions for SARS-CoV-2 associated **m**ultisystem **i**nflammatory **s**yndrome in **c**hildren (MIS-C).

**Methods:**

The **B**est **A**vailable **T**reatment **S**tudy (BATS) evaluated immunomodulatory treatments for MIS-C in an international observational cohort. Clinical and outcome data were collected using webbased enrollment. Inverse probability weighting and generalized linear models were used to compare treatments with intravenous immunoglobulin (IVIG), IVIG plus glucocorticoids (IVIG+G), or glucocorticoids alone. Primary outcomes were inotropic or ventilator support day 2 or later, or death; and improvement on an ordinal severity scale by day 2. Secondary outcomes included treatment escalation and time to improvement of organ failure and inflammation.

**Results:**

Courses of 614 children from 32 countries enrolled between June 2020 and February 2021 were analyzed. 490 met WHO MIS-C criteria. 246 received primary treatment with IVIG; 208 IVIG and glucocorticoids (IVIG+G); 99 glucocorticoids alone; 22 other combinations including biologicals, and 39 no immunomodulator.

The odds ratios (95% confidence interval) relative to IVIG for ventilation, inotropic support or death were 0.77 (0.33-1.82) and 0.54 (0.22-1.33) for IVIG+G and glucocorticoids, respectively, and for ordinal scale improvement, 0.90 (0.48-1.69) and 0.93 (0.43-2.04), respectively. The odds ratio for immunomodulator treatment escalation for IVIG+G compared to IVIG was 0.18 (CI 0.10-0.33), and glucocorticoids compared to IVIG was 1.31 (0.64-2.68). Time to improvement was similar for the three treatments.

**Conclusion:**

We did not find evidence that recovery rate differs following primary treatment of MIS-C with IVIG, glucocorticoids, or IVIG+G, although significant differences may emerge as more data accrue. (ISRCTN registry - ISRCTN69546370.)

Since recognition in April 2020, **m**ulti-system **i**nflammatory **s**yndrome in **c**hildren (MIS-C), temporally associated with SARS-CoV-2 infection,^1–4^ has emerged as a rare but serious post-infectious illness.^5–8^ MIS-C occurs 2-6 weeks after SARS-CoV-2 infection and is characterized by persistent fever, and non-specific symptoms, particularly abdominal pain, vomiting, headache, and fatigue. Conjunctival injection and rash resembling Kawasaki disease (KD) occur in a high proportion of patients.^4,6,9–11^ Severely affected children develop shock, requiring inotropic support, and multi-organ failure. Laboratory studies show intense inflammation with elevation of CRP, ferritin, troponin and pro-brain natriuretic peptide (pro-BNP), and reduced hemoglobin, platelets and lymphocytes.

Faced with a new disease with no proven therapy, pediatricians have treated patients using their “best guess” as to what might be beneficial. Based on resemblance of MIS-C to KD ^2,4,9,11^, macrophage activation syndrome,^12^ and staphylococcal toxic shock syndrome,^13,14^ immunomodulatory agents, which have shown benefit in these diseases have been preferentially chosen, often used in combination, or sequentially when initial treatments failed.^15–17^

As coronary artery aneurysms (CAA) are an important overlapping feature of both MIS-C and KD, intravenous immunoglobulin (IVIG), the proven treatment for KD,^18^ has been widely adopted as initial therapy, and withholding IVIG is considered unacceptable by some clinicians. However, as some patients recover with supportive treatment alone,^3,15^ aggressive attempts to suppress the inflammatory response may not necessarily translate into clinical benefit.

Randomized trials are ideally needed to establish optimal treatment of MIS-C. However, the speed with which MIS-C emerged, and concern that it may lead to death, multiorgan failure and long-term cardiac damage has led to a range of unproven immunomodulators being adopted, and recommended in local and national treatment guidelines.^19,20^

The **B**est **A**vailable **T**reatment **S**tudy (BATS) aimed to provide evidence for treatment recommendations for MIS-C by systematic collection, and analysis of outcomes of the treatments chosen by individual pediatricians responsible for patient care.

## Methods

Pediatricians world-wide were invited to join the present study and upload data from patients with MIS-C onto a web-based REDCap database.^21^ As accuracy of current MIS-C definitions are unknown and emerging experience suggests a wide spectrum of inflammatory illness following SARS-CoV-2 infection,^22,23^ our study invited pediatricians to enroll not only patients meeting published criteria^24–26^ but also children with suspected inflammatory illness post SARS-CoV-2. De-identified longitudinal data were collected on presenting features, demography, laboratory findings, immunomodulatory (IVIG, glucocorticoids or biologicals) and supportive treatments. Treatments and daily data were collected by calendar day. Duration of admission, organ support required, and health status on discharge were recorded (see the Supplementary Appendix for the study handbook). The protocol is available at NEJM.org.

### Treatments and End Points

The first calendar day of immunomodulatory treatment was defined as day 0, and subsequent treatment and outcomes defined relative to this. Additional immunomodulators administered on the same calendar day as the primary treatment were defined as co-primary treatments, while those on subsequent days were considered secondary treatments.

Primary outcomes were first, a composite of inotropic support on day 2 or later, ventilator support (invasive or noninvasive) on day 2 or later, and death; and second, improvement between day 0 and day 2 of at least one level on an ordinal clinical severity scale (ventilated and on inotropic support; ventilated; on inotropic support; receiving oxygen; no supportive therapy with CRP 50 mg/l or more; no supportive therapy with CRP below 50mg/l; and discharged).

Secondary analyses included temporal dynamics of blood markers of inflammation and organ damage; immunomodulator escalation (any additional immunomodulator, and if initial treatment included glucocorticoids, an increment of 5 mg/kg equivalent daily dose of prednisolone were considered secondary treatments); time to one-point improvement in the ordinal severity scale; left ventricular (LV) dysfunction on echocardiography and CAA following treatment (coronary artery Z-score ≥2.5 or aneurysm documented)^27^, and any increase in cardiorespiratory supportive therapy after day 0; death and therapeutic complications.

We performed subgroup analysis of patients meeting the WHO MIS-C criteria, and an alternative definition of primary treatments as those received on days 0 and 1, with escalation being additional treatment from day 2.

### Analysis and Statistics

Analyses of dichotomous outcomes used weighted logistic regression and time-to-event analyses used weighted Cox regression with weights determined by inverse probability weighting using covariate-balancing propensity scores to account for baseline differences between treatment groups (IVIG, glucocorticoids, and IVIG and glucocorticoids (IVIG+G)). These analyses included the same covariates as used for the propensity scores to produce doubly robust estimates. The average treatment effect was estimated except where described otherwise. Outcomes were reported as odds ratios (ORs) or average hazard ratios (AHR) with 95% confidence intervals (CIs) (see Supplementary Methods).

Inflammatory markers were plotted as percentages of each patient’s peak value. Weighted generalized additive models (GAM) were fitted to produce smoothed curves with confidence intervals. Weights were produced as above, but for untreated patients the average treatment effect in the untreated was estimated, to preserve the sample size of this smaller and more distinct subgroup.

### Oversight

The study was designed by the study team at Imperial College London (members and roles in Supplementary Appendix). Patient data were collected by local investigators (Consortium members, Supplementary Appendix). The statistical analysis plan was developed by the statistical group (see Supplementary Appendix) who also undertook the analysis. Study progress, and publication strategy were overseen by an international advisory board (see Supplementary Appendix). The study was approved by the UK REC (20/HRA/2957) and registered with the international trial registry (ISRCTN69546370). Participating centers obtained ethical approval based on requirements in each country. The initial manuscript was drafted by the corresponding author and developed by all members of the writing group. The corresponding author, data management group, and analysis group had access to all data, vouching for the completeness and accuracy of data, and for fidelity to the protocol and analysis plan.

## Results

From June 20, 2020 to February 24, 2021, data from 651 MIS-C patients from 34 countries and 81 hospitals were uploaded to the study database (Supplementary Appendix, Figs. S1, S2, S3). Thirty-seven were excluded for incomplete data or duplicate entries (Fig.1A); 246 received primary treatment with IVIG, 99 with glucocorticoids, 208 with combination IVIG+G, 22 with other immunomodulators, and 39 with no immunomodulators (Fig.1A). In the primary treatment arms, 136 patients (26%) received additional immunomodulators by day 2, with 238 receiving secondary agents in total (44%). The complex changes in treatments are shown in Fig.1B and Supplementary Appendix, Table S1).

Clinical and laboratory findings were similar between treatment groups (Table 1 and Table S2), but troponin levels were higher in the IVIG+G group (Fig. S4, S5), as were the proportion of patients receiving inotropes on day 0 (Fig. S6A,S6B).

490/614 patients (80%) met WHO MIS-C criteria (Table S3). The most common criterion missing was evidence of SARS-CoV-2 exposure (Fig. S7). SARS-CoV-2 antibody measurements were not tested in 14%, and negative in 14%. Bacteria were cultured in blood of a small proportion of patients (Table S3).

37% overall, and 39% of those meeting WHO MIS-C criteria also met the American Heart Association (AHA) definitions for KD^18^ (Table S4, Fig. S8).

### Primary Outcomes

**Figure 2:**
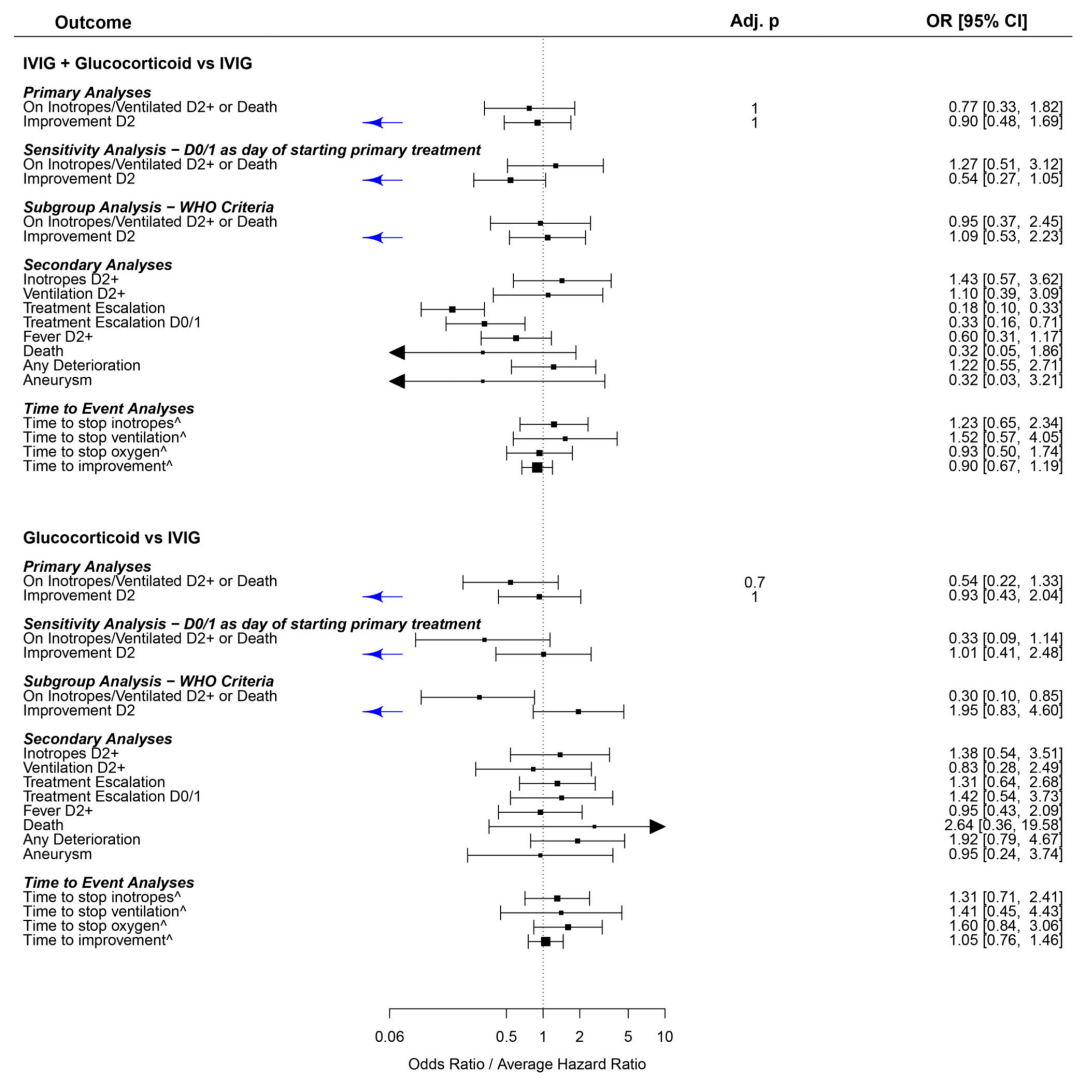
(A) Forest plot summarizing point estimates and 95% confidence intervals for primary, secondary and subgroup analyses. Displayed values are odds ratios or average hazard ratios (indicated by ^A^ for time-to-event analyses). The blue arrows indicate outcomes for which the direction of superiority is reversed.

Fifty patients (9.0%) received immunomodulators prior to transfer to the reporting hospital and were excluded from weighted analyses. For the primary outcome of receipt of inotropic support or ventilation on day 2 or later, or death, the odds ratios (OR) (95% CIs) for patients receiving initial treatment with IVIG+G, or glucocorticoids alone as compared with IVIG were 0.77 (0.33-1.82) and 0.54 (0.22-1.33) respectively (Fig. 2). Unadjusted numbers shown in Table S5.

Subgroup analysis, including only those meeting WHO MIS-C criteria, produced OR for IVIG+G and glucocorticoids each versus IVIG of 0.95 (0.37-2.45) and 0.30 (0.10-0.85), respectively (Fig. 2, Table S5B). Individual components of the composite outcome showed no apparent differences. For improvement in ordinal clinical severity scale by day 2, the OR for patients receiving IVIG+G vs. IVIG was 0.90 (0.48-1.69) and those receiving glucocorticoids alone vs. IVIG was 0.93 (0.43-2.04).

When WHO criteria for MIS-C were considered in subgroup analysis, the OR for improving ordinal scale was 1.09 (0.53-2.23) for IVIG+G, and 1.95 (0.83-4.60) for glucocorticoids alone vs. IVIG: (Fig. 2; Table S5B). Acceptable covariate balance was achieved for both primary outcome analyses (Fig. S9). Analyses with standardized weights yielded similar inferences.

### Secondary Outcomes

Escalation of immunomodulator treatment was less common in the IVIG+G group than the IVIG group (OR 0.18 [0.10-0.33]), but inconclusive between glucocorticoid and IVIG and groups (OR 1.31 [0.64-2.68]) (Table S5). No clear differences were seen in blood markers, inotropic support or ventilation between patients who escalated to other treatments by day 2 and those remaining on primary treatment (Fig. S5; Fig. S6B).

Left ventricular dysfunction was reported in 11.8% of patients with echocardiograms from day 2 onwards, with no apparent differences between groups. Coronary artery aneurysms were present on the latest echocardiogram (two days following initiation of treatment or later) in 6.1% of patients with echocardiograms reported in this period, but 35% reported no results. There was no apparent increase in CAA in patients not receiving IVIG, but numbers with post treatment echocardiogram were low (Table S6). Death was reported in 5/192, 4/91 and 3/238 patients in the IVIG+G, glucocorticoid and IVIG groups, respectively (Table S5).

In time-to-event analyses of improvement on the ordinal severity scale, AHRs (95% CIs) were: IVIG+G vs. IVIG 0.89 (0.67-1.19); glucocorticoid vs. IVIG 1.03 (0.72-1.46) (Fig. S9).

Drug complications were reported by clinicians in 16 (3.9%) patients receiving any glucocorticoids and 9 (1.8%) patients receiving IVIG. Glucocorticoid-related complications were predominantly hypertension and hyperglycemia (Table S7).

### Effect of Immunomodulation on Blood Markers

**Figure 3:**
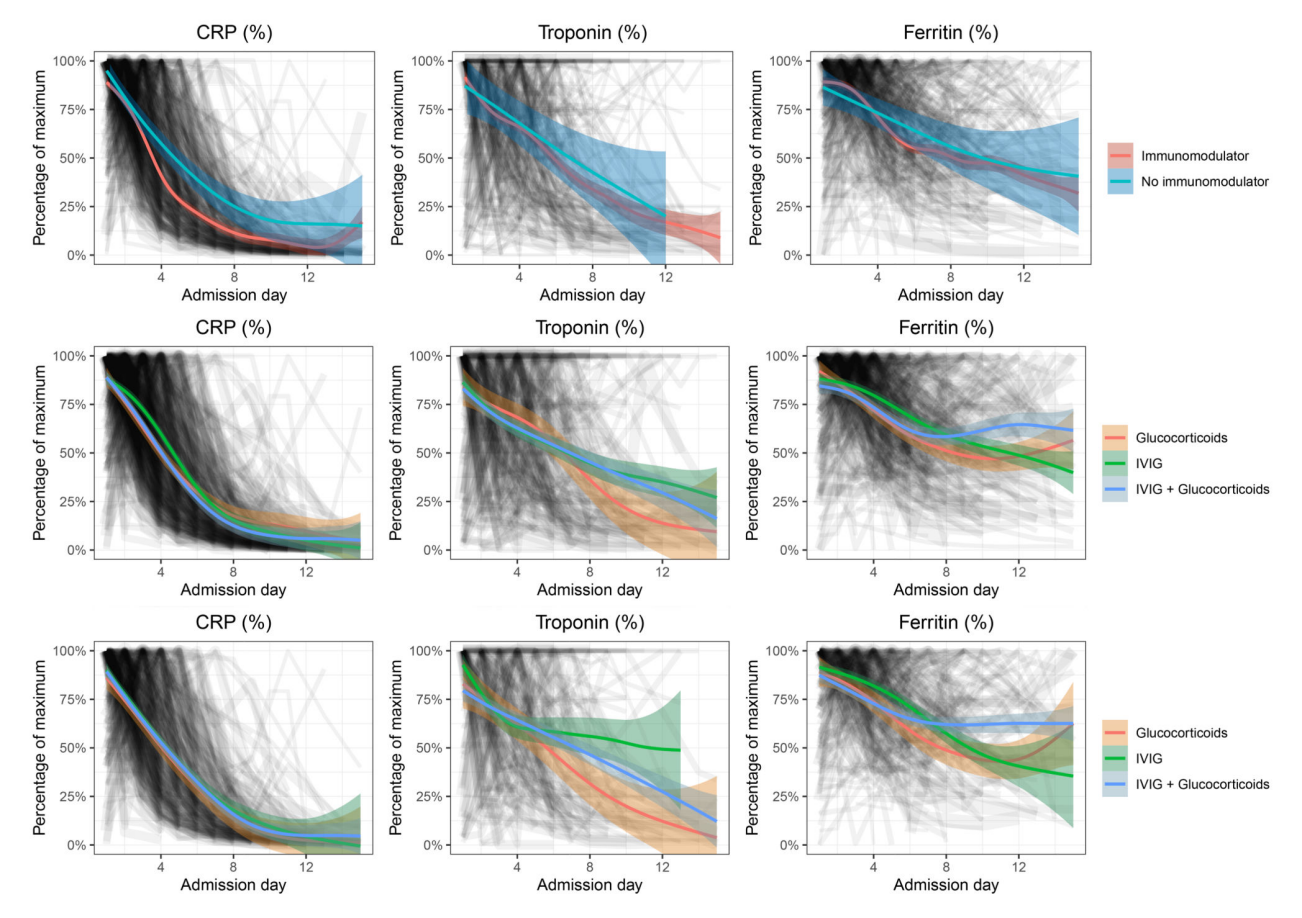
Change in C-reactive protein (CRP), troponin and ferritin over time from admission. Percentage of the maximum value of CRP, troponin and ferritin for each patient at each time point (day) are plotted as a line and weighted by CBPS. A generalized additive model (GAM) was used to fit the curves. Panel A shows the fitted curves for CRP, troponin and ferritin of patients who received any immunomodulators versus patients who did not receive immunomodulating treatments. Panel B shows the fitted curves for patients in the IVIG, IVIG+G and glucocorticoid primary treatment groups. Panel C shows the fitted curves for IVIG, glucocorticoids and IVIG+G combined for those patients whose primary treatment did not change between the day of admission and day 3.

Changes over time in CRP, ferritin and troponin were compared between individuals receiving different treatments. CRP appeared to decline more rapidly in patients receiving immunomodulators than those not receiving treatment (Fig. 1A). Changes in CRP, troponin and ferritin followed a similar temporal trend for patients receiving primary treatment with IVIG, glucocorticoid, and IVIG+G (Fig. 3B), although there was some variation in the rate of decline which was most obvious in subjects who did not change treatment before day 3 (Fig. 3C).

To investigate whether inclusion of children with KD within the present study might have influenced treatment responses, we explored changes in blood markers separately in children likely to have KD versus not. As KD is generally a disease in children aged 5 years and below and MIS-C is generally reported in older children, we compared those meeting AHA criteria for KD, and all children under 6 years (which may be “KD-like”), with the remaining MIS-C patients. The smoothed curves showed similar rates of decline in CRP in children under 6 with features of KD compared to others who received IVIG, but there was a suggestion that children without KD criteria may have slightly more rapid decline in CRP in response to glucocorticoids (Fig. S10).

## Discussion

In a pragmatically-defined international MIS-C cohort we have not found evidence that the most common treatments (glucocorticoids alone, IVIG alone and IVIG+G) are associated with differences in primary outcomes (requirements for inotropic support, ventilation on day two or beyond, or death; or improvement on the ordinal severity scale by day two). However, when restricted to patients meeting the WHO criteria, there is modest, yet inconclusive evidence for benefit of glucocorticoids vs. IVIG for both primary outcomes.

Analysis of secondary outcomes showed lower rates of immunomodulator treatment escalation (addition of secondary agents) in patients receiving IVIG+G. We did not find clear evidence for association between initial treatment with glucocorticoids, IVIG+G, or IVIG and progression to, or time to improvement in organ failure, resolution of inflammation, or discharge from hospital. CAA rates were similar between IVIG-containing regimes and the glucocorticoid treated groups, but numbers with echocardiographic studies after treatment were too low for firm conclusions to be made.

A French national surveillance study of MIS-C^28^ also found lower rates of treatment escalation in patients receiving IVIG+G than IVIG alone, and suggested reduced need for hemodynamic support, and time in intensive care with combination IVIG+G. Their study did not include a glucocorticoid-only group and had smaller numbers of patients than our study.

The high rates of escalation to additional treatments in patients receiving single agents may be explained by a greater readiness to escalate when only one treatment was given, by failure of initial treatment, or by severity of illness in those escalating. A higher proportion of patients receiving IVIG+G were receiving inotropes at day 0 and they had higher levels of troponin suggesting more severely ill patients may have received IVIG+G. However, patients receiving additional treatment by day 2 did not have greater inotrope or ventilation support or troponin levels than non-escalating patients.

We explored how the different agents might affect inflammation using CRP and ferritin as surrogates for overall inflammation, and troponin as marker of cardiac injury. Controlling for baseline differences in severity, rate of reduction in CRP appeared more rapid in those receiving immunomodulators.

As clinical features of MIS-C overlap those of KD, a major dilemma in treatment decisions for MIS-C has been whether treatment regimens that do not include IVIG, the proven treatment for KD, may delay recovery and increase risk of CAA. We found no evidence for delayed recovery from organ failure in patients receiving glucocorticoids as initial treatment. When restricted to patients meeting the WHO MIS-C criteria in subgroup analysis, there was suggestive benefit of glucocorticoids in reducing organ failure and ordinal scale improvement, although a proportion escalated to IVIG+G. The rates of CAA across all three treatment groups were low (6%) and firm conclusions could not be drawn from the small number in each treatment group.

We explored whether inadvertent inclusion of KD among the MIS-C group might have prevented detection of benefit from non-IVIG treatments. We found suggestive evidence that the rate of decline in CRP associated with glucocorticoid treatment may differ between younger children meeting KD criteria and other children with MIS-C, supporting our concern that the challenges in distinguishing between these two diseases clinically may make it more difficult to identify differences between treatments for MIS-C.

Our study included patients meeting the WHO definition of MIS-C as well as patients lacking one or more diagnostic criteria. The current criteria are imperfect, as antibody testing is not always available and history of exposure to SARS-CoV-2 lacks specificity as asymptomatic infection is common. MIS-C is a spectrum of illness, and until a diagnostic test is developed, clinicians will face difficult treatment decisions on a wider group of inflammatory diseases following SARS-CoV-2 than are identified by WHO criteria.

MIS-C has emerged as an important childhood problem in low- and middle-income countries^29,30^. As IVIG and biological agents are costly, and have limited availability in many countries, evidence to support their use in preference to cheaper anti-inflammatory agents such as glucocorticoids is needed. As the current study does not provide conclusive evidence for either equivalence or superiority of any of three treatments evaluated, ongoing recruitment and analysis of a larger number of patients is needed to provide definitive evidence.

This study has some limitations. A major concern in use of non-randomized studies such as this one, is whether treatment selection is biased by illness severity. Propensity score weighting reduces bias. However, bias may also have arisen from patients receiving additional treatments after primary treatment. Higher rates of treatment escalation in single agent groups may mask differences in efficacy. Additionally, 35% of the study participants had no echocardiogram reported during the study period, and follow up data on coronary artery aneurysms after treatment were not reported for the majority of patients, thus the current findings are insufficient to suggest equivalence of glucocorticoids with IVIG in preventing coronary artery aneurysms.

In conclusion, we did not find evidence of differences in outcomes between treatment with glucocorticoids or IVIG as single agents, or between the single and dual agent primary treatments. The confidence intervals for inferences about treatment effect admit the possibility of actual benefit from one or more of the treatments relative to the others.

## Supplementary Material

Supplement

## Figures and Tables

**Figure 1A:**
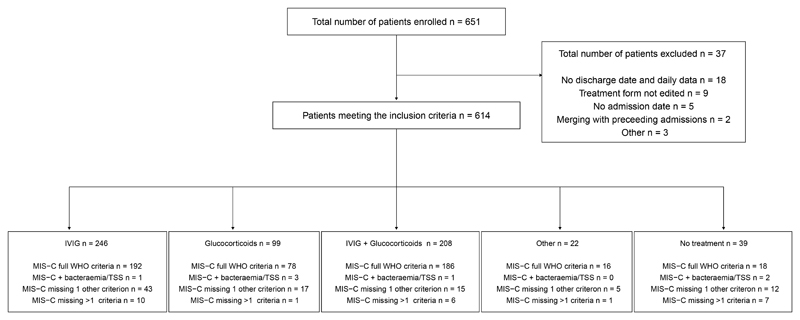
Study flowchart The study flow chart gives an overview of the total number of patients enrolled, excluded and included for the analyses. Patients meeting the inclusion criteria are categorized by treatment groups (IVIG, glucocorticoids, IVIG and glucocorticoids, Other [this includes: anti-tumor necrosis factor, anti-interleukin 1, anti-interleukin 6] and No treatment) and subdivided by WHO clinical criteria.

**Figure 1B:**
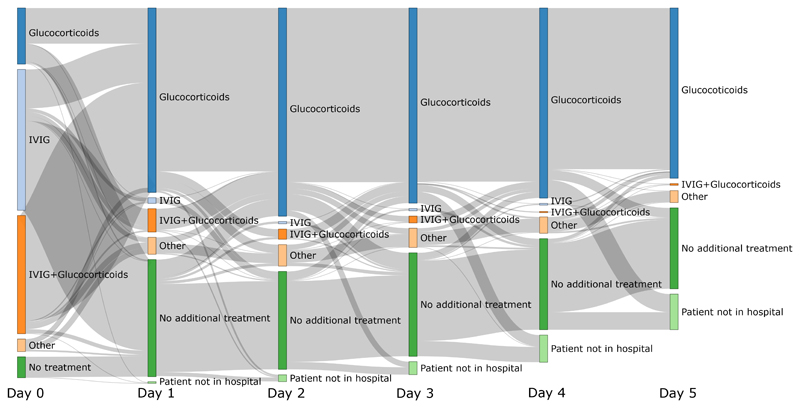
Treatments received by patients following admission over time. The Sankey diagram demonstrates the number of patients that received each treatment on days following admission. Each vertical stack represents a different day in the patients’ admission (Days 0 – 5). The grey bands represent movement of patients between treatment groups from day 0 to 1, day 1 to 2, day 2 to 3, day 3 to 4 and day 4 to 5. The width of the grey bands is proportional to the number of patients (flow). The flow of patients is independent between day 0 to 1, day 1 to 2, day 2 to 3, day 3 to 4 and day 4 to 5; there is no continuous correspondence across days 1 to 5. The treatment groups are as stated. Of note, “glucocorticoids” include intravenous and oral glucocorticoids, and daily doses of glucocorticoids as part of a glucocorticoid course do not constitute an additional treatment. “Other” includes one or more other immunomodulatory treatment(s) given alone or in combination with glucocorticoids and/or IVIG. Other immunomodulatory treatments include: anti-interleukin1, anti-interleukin 6, anti-tumor necrosis factor, Cytosorb, granulocyte colony stimulating factor, colchicine, mesenchymal stem cells and convalescent plasma.

**Table 1:** Clinical and demographic features in all treatment groups

	Everyone(N=614)	IVIG (N=246)	Glucocorticoi ds (N=99)	IVIG and glucocorticoid s (N=208)	Other (N=22)	No treatment (N=39)
^+^ **Age**	8.3 [4.2 - 12]	7.0 [3.7 - 11]	8.8 [5.0 - 12]	8.8 [4.6 - 12]	13 [9.5 - 15]	9.6 [4.4 - 13]
^ **Proportion male**	376 (61.2%)	157 (63.8%)	59 (59.6%)	127 (61.1%)	15 (68.2%)	18 (46.2%)
**(age-adjusted z score ≥ 2)**	90 (14.7%)	28 (11.4%)	10 (10.1%)	45 (21.6%)	4 (18.2%)	3 (7.69%)
^ **Significant comorbidity**	21 (3.42%)	5 (2.03%)	7 (7.07%)	5 (2.40%)	1 (4.55%)	3 (7.69%)
^ **SARS-CoV-2 PCR positive**	133 (21.9%)	36 (14.8%)	26 (26.5%)	53 (26.0%)	8 (36.4%)	10 (26.3%)
^ **SARS-CoV-2 Ab positive**	424 (70.4%)	163 (67.6%)	68 (70.1%)	163 (80.3%)	16 (72.7%)	14 (35.9%)
^ **Organ support**	138 (22.5%)	38 (15.4%)	20 (20.2%)	66 (31.7%)	8 (36.4%)	6 (15.4%)
^ **Clinical features on admission**						
Fever	580 (94.5%)	237 (96.3%)	92 (92.9%)	196 (94.2%)	20 (90.9%)	35 (89.7%)
Sore throat	149 (27.9%)	62 (30.1%)	23 (25.6%)	50 (26.2%)	3 (17.6%)	11 (35.5%)
Cough	124 (21.8%)	49 (21.4%)	24 (25.3%)	38 (19.6%)	7 (35.0%)	6 (19.4%)
Respiratory distress	88 (15.3%)	29 (12.8%)	12 (12.5%)	36 (18.2%)	6 (28.6%)	5 (14.3%)
Abdominal pain	365 (63.7%)	142 (63.7%)	48 (51.6%)	138 (69.0%)	16 (72.7%)	21 (60.0%)
Diarrhea	281 (48.0%)	100 (43.3%)	36 (38.3%)	120 (58.8%)	7 (31.8%)	18 (51.4%)
Vomiting	324 (56.1%)	118 (52.0%)	43 (45.7%)	135 (66.5%)	13 (65.0%)	15 (44.1%)
Headache	164 (31.6%)	66 (33.3%)	22 (25.6%)	61 (33.2%)	7 (35.0%)	8 (25.8%)
Irritability	116 (20.9%)	39 (18.1%)	22 (23.9%)	47 (23.9%)	1 (5.56%)	7 (21.9%)
Lethargy	222 (39.4%)	89 (40.8%)	46 (47.9%)	64 (32.3%)	11 (55.0%)	12 (37.5%)
^ **KD criteria**	225 (36.6%)	106 (43.1%)	30 (30.3%)	82 (39.4%)	5 (22.7%)	2 (5.13%)
^+^ **Bloods on admission**						
Lymphocytes (10^9/L)	1.2 [0.74 - 1.9]	1.4 [0.80 - 2.2]	1.1 [0.76 - 1.7]	1.1 [0.70 - 1.7]	0.81 [0.48 - 1.5]	1.1 [0.78 - 2.3]
Troponin (ng/L)	42 [10 - 190]	18 [8.0 - 55]	50 [16 - 150]	50 [30 - 260]	200 [13 - 2900]	11 [7.3 - 120]
CRP (mg/L)	150 [90 - 230]	150 [82 - 210]	130 [50 - 250]	150 [90 - 250]	160 [120 - 260]	160 [67 - 200]
Ferritin (ug/L)	460 [230 - 860]	410 [200 - 620]	530 [230 - 1100]	560 [300 - 920]	640 [310 - 1300]	230 [140 - 330]
Albumin (g/L)	33 [28 - 38]	34 [29 - 40]	31 [27 - 34]	32 [28 - 38]	32 [29 - 37]	34 [30 - 39]

Descriptive table of demographic features, clinical features and blood markers on admission, and proportion of patients meeting Kawasaki Disease criteria according to American Heart Association criteria. Patients with coronary artery aneurysms met the definition of Kawasaki Disease with less than 4 Kawasaki Disease clinical features. Patients were divided by treatment arm on day 0 (IVIG alone, glucocorticoids alone, IVIG+G, no treatment, and other (any other treatment combination including biologicals)). SARS-CoV-2 PCR data refer to test taken during admission. Organ support refers to receipt of ventilation, inotropes or ECMO on admission. Missing data (where applicable) are available in a full unabridged version in Table S1. Abbreviations: Ab: Antibody; CRP: C-reactive protein; ECMO: extracorporeal membrane oxygenation; PCR: polymerase chain reaction.

^ Clinical and demographic features given as raw values and (%).

+ Numerical values given as median values and [interquartile ranges].
