# Supplementary material for: Immunomodulatory Treatment of Multisystem Inflammatory Syndrome in Children
Source: N Engl J Med. Author manuscript; Available in PMC 2021 Jul 19. (PMC8220965; doi:10.1056/NEJMoa2102968)
Supplement: Supplement [file EMS126605-supplement-Supplement.pdf]

SUPPLEMENTARY APPENDIX

Table of Contents

The Best Available Treatment Study (BATS) Consortium 3

Supplementary Methods 11

Patient Recruitment 11

Data preparation 11

Primary outcome definitions: 12

Secondary outcomes: 13

Analysis 14

Supplementary Tables 17

Table S1| Details of additional treatments given by initial immunomodulatory therapy groups. 17

Table S2 | Unabridged clinical features, demographic information, and blood results for all patients included in analysis. 17

Table S3 | Clinical features, demographic information, and blood results for all patients included in analysis and subgroups meeting more restricted criteria 21

Table S4 | Distribution of patients meeting WHO criteria subdivided by Kawasaki Disease status and initial treatment given. 25

Table S5 | Primary Outcomes and Sensitivity Analyses 26

Table S5 | Secondary Outcomes and Time to Event Analyses 27

Table S6 | Coronary artery aneurysms by initial immunomodulatory therapy groups 32

Table S7 | Treatment related complications 32

Treatment related complications reported by clinicians. 32

Supplementary Figures 36

Figure S1 | World map displaying the location of countries registered to the Best Available Treatment Study. 37

Figure S2a | Number of enrolment sites registered per country. 37

Figure S2b | Number of patients enrolled in BATS per country. 38

Figure S2c | Number of patients enrolled in BATS by sites in each country. 38

Figure S3 | BATS registration by month between May 2020 and February 2021. 39

Figure S4 | Comparison of blood results across treatment groups at day 0. 40

Figure S5 | Comparison of baseline CRP and troponin across treatment groups between day 0 and day 2 41

Figure S6A | Proportion of patients on inotropes or ventilated at baseline across treatment arms at day 0. 42

Figure S6B | Proportion of patients on inotropes or ventilated at baseline across treatment groups between day 0 and day 2. 43

Figure S7 | Missing components in patients with all but one component of the WHO MIS-C criteria. 44

Figure S8 | Proportion of patients with clinical features of Kawasaki disease across treatment groups at day 0.. 45

Figure S10 | Percentage of the CRP peak value by admission day for three primary treatments (IVIG, glucocortioids and IVIG and glucocorticoids combined). 47

Figure S11 | Inverse probability weight distributions and covariate balance plots 48

References 50

The Best Available Treatment Study (BATS) Consortium

The BATS consortium (https://bestavailabletreatmentstudy.co.uk) is composed, in alphabetical order for each country, by:

Imperial College London (Study Management group):

Study Coordinator: Professor Michael Levin

Co-investigators: Dr Claire Broderick, Dr Aubrey Cunnington, Dr Jethro Herberg, Dr Myrsini Kaforou, Dr Andrew McArdle, Dr Ruud Nijman, Dr Harsita Patel, Dr Eleanor Seaby, Dr Priyen Shah, Ms Ortensia Vito, Dr Elizabeth Whittaker, Dr Clare Wilson

Statisticians: Dr Clive Hoggart, Dr Myrsini Kaforou, Dr Andrew McArdle

Data management: Dr Tisham De, Ms Ortensia Vito

International Advisory Board:

Daniel Munblit[1], Adriana Tremoulet[2], Rolando Ulloa-Gutierrez[3]

[1]*Department of Pediatrics and Pediatric Infectious Diseases, Institute of Child's Health, Sechenov First Moscow State Medical University (Sechenov University), Moscow, Russia;* [2]*Department of Pediatrics, Rady Children's Hospital - San Diego, 3020 Children's Way, San Diego, CA 92123;* [3]*Servicio de Infectología Pediátrica, Hospital Nacional de Niños "Dr. Carlos Sáenz Herrera", C.C.S.C., San José, Costa Rica*

ARGENTINA

Jorge Agrimbau Vázquez[1], Rodrigo Carmona[2], Laura Pérez[3], Mayra Rubiños[4], Natalia Veliz[4], Silvana Yori[1]

[1] *Department of Pediatrics, Outpatient Services, Hospital de Pediatría "Prof. Dr. Juan P. Garrahan", Buenos Aires, Argentina*

[2] *Pediatric Critical Care Unit, Hospital de Pediatría "Prof. Dr. Juan P. Garrahan", Buenos Aires, Argentina*

[3] *Department of Pediatrics, Emergency Medicine, Hospital de Pediatría "Prof. Dr. Juan P. Garrahan", Buenos Aires, Argentina*

[4] *Department of Pediatrics, Internal Medicine, Hospital de Pediatría "Prof. Dr. Juan P. Garrahan", Buenos Aires, Argentina*

AUSTRIA

Wolfgang Holter[1], Matthias Krainz[1], Raphael Ulreich[2], Christoph Zurl[2]

[1] *St. Anna Children's Hospital, Medical University of Vienna, Vienna, Austria*

[2] *Department of Pediatrics, Pediatric Intensive Care Unit, Medical University Graz, Austria*

BELGIUM

Filomeen Haerynck[1], Levi Hoste[2]

[1] *Primary Immunodeficiency Research Lab, Center for Primary Immunodeficiency Ghent, Jeffrey Modell Diagnosis and Research Center, Ghent University Hospital, Ghent, Belgium*

[2] *Department of Pediatric Pulmonology, Infectious Diseases and Immunology, Ghent University Hospital, Ghent, Belgium*

BRAZIL

Izabel Alves Leal[4], André Ricardo Araujo da Silva[2], Anna Esther Araujo e Silva[1], Andrea Barchik[4], Sabrina T. A. Barreiro[1], Natalia Cochrane[4], Cristiane Henriques Teixeira[4], Julienne Martins Araujo[1], Rolando Andres Paternina-de la Ossa[3], Cristina Souza Vieira[4]

[1] *Department of Pediatrics, Getulio Vargas Filho Hospital, Niteroi, RJ, Brazil*
[2] *Materno Infantil Department, Federal Fluminense University, Brazil*
[3] *Faculdade de Medicina de Ribeirão Preto, Universidad de São Paolo, São Paolo, Brazil*
[4] *Prontobaby Group, Rio de Janeiro, Brazil*

BULGARIA

Anna Dimitrova[1], Margarita Ganeva[1], Stefan Stefanov[1], Albena Telcharova-Mihaylovska[1]
[1] *Department of pediatric rheumatology, University Children's Hospital, Medical University Sofia, Bulgaria*

CANADA

Catherine M. Biggs[1], Rosie Scuccimarri[2], Davinia Withington[2]
[1] *Department of Pediatrics, The University of British Columbia, Vancouver, BC, Canada*
[2] *Departments of Anesthesia and Pediatrics, Montreal Children's Hospital, McGill University Health Sciences Centre, Montreal, Quebec H4A 3J1, Canada*

CHILE

Camila Ampuero[1], Javiera Aravena[2], Raul Bustos B[3], Daniel Casanova[4], Pablo Cruces[4,5,6], Franco Diaz[4,6,7], Tamara García-Salum[2], Loreto Godoy[8], Rafael A. Medina[2], Gonzalo Valenzuela Galaz[2]
[1] *Unidad de Paciente Crítico, Hospital Clínico La Florida, Santiago, Chile*
[2] *Department of Pediatric Infectious Diseases and Immunology, Pontifical Catholic University of Chile, Marcoleta 391, Santiago, Chile*
[3] *Pediatric Intensive Care, Clínica Sanatorio Alemán, Concepción, Chile*
[4] *Unidad de Paciente Crítico Pediátrico, Hospital de El Carmen de Maipú, Santiago, Chile*
[5] *Centro de Investigación de Medicina Veterinaria, Escuela de Medicina Veterinaria, Facultad de Ciencias de la Vida, Universidad Andres Bello, Santiago, Chile*
[6] *LARed Network, Santiago, Chile*
[7] *Escuela de Medicina, Universidad Finis Terrae, Santiago, Chile*
[8] *Unidad de Paciente Crítico Pediátrico (UPCP), Complejo Asistencial Dr. Sotero del Río, Santiago, Chile*

COSTA RICA

María L. Avila-Aguero[1,4], Helena Brenes-Chacón[1], Gabriela Ivankovich-Escoto[2], Rolando Ulloa-Gutierrez[1], Adriana Yock-Corrales[1]
[1] *Servicio de Infectología Pediátrica, Hospital Nacional de Niños "Dr. Carlos Sáenz Herrera", C.C.S.C., San José, Costa Rica*
[2] *Servicio de Immunología y Reumatología Pediátrica, Hospital Nacional de Niños "Dr. Carlos Sáenz Herrera", C.C.S.C., San José, Costa Rica*
[3] *Servicio de Emergencias Pediátricas, Hospital Nacional de Niños "Dr. Carlos Sáenz Herrera", C.C.S.C., San José, Costa Rica*
[4] *Center for Infectious Disease Modeling and Analysis (CIDMA), Yale University New Haven, Haven, CT, USA*

EGYPT

Adham Badib[1], Karim Badreldin[1], Yara Elkhashab[1], Hassan Heshmat[1]
[1] *Neonatal Intensive Care Unit, Smouha International Hospital, Alexandria, Egypt*

FINLAND

Santtu Heinonen[1]
[1] *New Children's Hospital, Pediatric Research Center, University of Helsinki, and Helsinki University Hospital, 00029 HUS, Helsinki, Finland*

FRANCE

François Angoulvant[1,2], Alexandre Belot[3], Naïm Ouldali[4,5,6]
[1] Assistance Publique–Hôpitaux de Paris, Pediatric Emergency Department, Necker-Enfants Malades University Hospital, Université de Paris, Paris, France

[2] INSERM, Centre de Recherche des Cordeliers, UMRS 1138, Sorbonne Université, Université de Paris, Paris, France
[3] Hospices Civils de Lyon, Pediatric Nephrology, Rheumatology, Dermatology, Hopital Femme, Mère Enfant, Centre International de Recherche en Infectiologie/INSERM U1111, Bron, France
[4] Assistance Publique–Hôpitaux de Paris, Department of General Pediatrics, Pediatric Infectious Disease and Internal Medicine, Robert Debré University Hospital, Université de Paris, Paris, France
[5] ACTIV, Association Clinique et Thérapeutique Infantile du Val-de-Marne, Créteil, France
[6] Université de Paris, INSERM UMR 1123, ECEVE, Paris, France

GERMANY

Florian Beske[1], Axel Heep[1], Katja Masjosthusmann[2], Karl Reiter[3], Ingeborg van den Heuvel[2], Ulrich von Both[3]
[1] *Department of Pediatrics, University Hospital Oldenburg, Rahel-Straus-Str. 10, 26133 Oldenburg, Germany*
[2] *University Hospital Muenster, Department of General Pediatrics, Albert-Schweizer-Campus A1, 48149 Muenster, Germany*
[3] *University Hospital, Ludwig Maximilians University (LMU) Munich, Hauner Children's Hospital, Germany*

GREECE

Aikaterini Agrafiotou[1], Charalampos Antachopoulos[2], Irini Eleftheriou[3], Evangelia Farmaki[4], Lampros Fotis[1,5,6], Dimitrios Kafetzis[6], Stavroula Lampidi[3], Theodota Liakopoulou[1], Despoina Maritsi[3], Elisa Michailidou[2], Maria Milioudi[2], Ioanna Mparmpounaki[1], Eleni Papadimitriou[2,4], Vassiliki Papaevangelou[5], Emmanuel Roilides[2], Olga Tsiatsiou[2], Georgios Tsolas[6], Maria Tsolia[3], Petrina Vantsi[2]
[1] *IASO Children's Hospital, Athens, Greece*
[2] *Infectious Diseases Unit, 3rd Department of Pediatrics, School of Medicine, Faculty of Health Sciences, Aristotle University, Hippokration General Hospital, Thessaloniki, Greece*
[3] *Second Department of Pediatrics, "P.& A. Kyriakou" Children's Hospital, Athens Medical School, National and Kapodistrian University of Athens, Greece*
[4] *First Dept of Pediatrics, Hippokratio Hospital of Thessaloniki, Aristotle of Thessaloniki, Greece*
[5] *ATTIKON General Hospital, Department of Pediatrics, National and Kapodsitrian University of Athens, Greece*
[6] *Metropolitan General Hospital, Piraeus, Greece*

HONDURAS

Linda Yajeira Banegas Pineda[1], Karla Leversia Borjas Aguilar[2], Edwin Mauricio Cantillano Quintero[1]
[1] *PICU, Department of Pediatric, North Hospital, IHSS, San Pedro Sula, Honduras*
[2] *Pediatric Immunology Service, Hospital María, Especialidades Pediátricas, Tegucigalpa, Honduras*

HONG KONG

Patrick Ip[1], Mike Yat Wah Kwan[2], Janette Kwok[4], Yu Lung Lau[1], Kelvin To[3], Joshua Sung Chih Wong[2]
[1] *Department of Pediatrics and Adolescent Medicine, Li Ka Shing Faculty of Medicine, The University of Hong Kong, Hong Kong SAR, China*
[2] *Pediatric Infectious Disease Unit, Hong Kong Hospital Authority Infectious Disease Center, Princess Margaret Hospital, Hong Kong SAR, China*
[3] *Department of Microbiology, Li Ka Shing Faculty of Medicine, The University of Hong Kong, Hong Kong SAR, China*
[4] *Department of Transplant and Immunogenetics, Queen Mary Hospital, Hong Kong SAR, China*

HUNGARY

Mate David[1], David Farkas[1], Szofia Kalcakosz[1], Klaudia Szekeres[1], Borbala Zsigmond[1]
[1] *General Pediatrics, Heim Pal Children Hospital, ulloi ut 86. Budapest 1089, Hungary*

INDIA
Nadeem Aslam[1]
[1] *Lotus Hospital for Women and Children, Pediatric ICU, Hyderabad, India*

ITALY
Laura Andreozzi[5], Francesco Bianco[1], Valentina Bucciarelli[1], Danilo Buonsenso[2], Rolando Cimaz[3], Patrizia D'Argenio[4], Rosa Maria Dellepiane[3], Marianna Fabi[5], Maria Vincenza Mastrolia[6], Angela Mauro[7], Angelo Mazza[8], Lorenza Romani[4], Gabriele Simonini[6], Vincenzo Tipo[7], Lucio Verdoni[8]
[1] *Department of Pediatric and Congenital Cardiology and Cardiac Surgery, Azienda Ospedaliero-Universitaria "Ospedali Riuniti", Ancona, Italy*
[2] *Department of Woman and Child Health and Public Health, Fondazione Policlinico Universitario A. Gemelli IRCCS, Rome, Italy*
[3] *University of Milano, Italy*
[4] *Immunology and Infectious Diseases Unit, Academic Department of Pediatrics, Bambino Gesù Children's Hospital, IRCCS, Piazza Sant'Onofrio, 4, 00165 Rome, Italy*
[5] *Department of Pediatrics, Sant'Orsola Malpighi University Hospital, Bologna, Italy*
[6] *Rheumatology Unit, Meyer Children's Hospital, University of Florence, Florence, Italy*
[7] *Emergency Department, COVID Unit, Santobono-Pausilipon Children's Hospital, Italy*
[8] *Pediatric General Department, ASST Papa Giovanni XXIII, Bergamo, Italy*

KENYA
Bhupi Reel[1]
[1] *MP Shah Hospital, Pediatric Intensive Care Unit, Nairobi, Kenya*

MALTA
David Pace[1], Paul Torpiano[1]
[1] *Department of Pediatric and Adolescent Health, Mater Dei Hospital, Malta*

MEXICO
Marisol Fonseca Flores[1], Miguel García Domínguez[2], Ana Luisa Giron Vargas[1], Liliana Lopez Hernández[1], Roanne Patrician Mota Figueroa[1], Giordano Pérez Gaxiola[2], Julio Valadez[3]
[1] *Department of pediatric critical care, Centro Medico Nacional Siglo XXI, Mexico City, Mexico*
[2] *Department of Allergy and Immunology, Hospital Pediatrico de Sinaloa, Culiacan, Mexico*

NORWAY
Sjur Klevberg[1], Per Kristian Knudsen[2], Per Helge Måseide[1]
[1] *Children Department, Drammen Hospital, Vestre Viken Hospital Trust, Norway*
[2] *Department of Pediatric Medicine, Oslo University Hospital, PB 4956 Nydalen, 0424 Oslo, Norway*

PANAMA
Jose Manuel Carrera[1], Elizabeth Castaño G[2], Carlos Alberto Daza Timana[1], Tirza De Leon[1], Dora Estripeaut[3], Jacqueline Levy Z[2], Ximena Norero[4], Javier Record[1], Magda Rojas-Bonilla[5]
[1] *Department of Infectious Disease, Hospital Materno Infantil José Domingo De Obaldía, David, Chiriquí, Panamá*
[2] *Pediatric Infectious Diseases Service, Hospital del Niño Dr. José Renán Esquivel, Panamá City, Panamá*
[3] *Pediatric Infectious Diseases, Hospital Paitilla, Panamá, Panamá and Sistema Nacional de Investigación (SNI), SENACYT, Panamá*
[4] *Pacifica Salud, Pediatric Infectious diseases, Panama City, Panama*
[5] *Department of Pediatrics Infectious Diseases, Hospital de Especialidades Pediatricas, Panama, Republic of Panama*

PARAGUAY
Ricardo Iramain[1]
[1] *Emergency Department, Hospital Clinicas-National University of Asuncion, Paraguay*

PERU
Roger Hernandez[1,2], Gian Huamán[1,2], Manuel Munaico[1,2], Carlos Peralta[1,2], Diego Seminario[1,2], Elmer Hans Zapata Yarlequé[1,2]
[1] *Departamento de pediatría, Hospital Cayetano Heredia, Lima, Perú*
[2] *Servicio de pediatría, Clínica San Felipe, Lima, Perú*

POLAND
Justyna Gadzinska[1], Joanna Mandziuk[1], Magdalena Okarska-Napierała[1]
[1] *Department of Pediatrics with Clinical Assessment Unit, Medical University of Warsaw, Żwirki i Wigury 63A Street, 02-091 Warsaw, Poland*

RUSSIA
Zalina A. Alacheva[1], Ekaterina Alexeeva[1,2], Petr V. Ananin[1], Margarita Antsupova[3], Maya D. Bakradze[1], Polina Bobkova[4], Svetlana Borzakova[5,6], Irina L. Chashchina[1], Andrey P. Fisenko[1], Marina S. Gautier[1], Anastasia Glazyrina[7], Elena Kondrikova[4], Evgeniya Korobyants[7], Anatoliy A. Korsunskiy[4], Karina Kovygina[4], Ekaterina Krasnaya[4], Seda Kurbanova[7], Maria K. Kurdup[1], Anna V. Mamutova[1], Lyudmila Mazankova[8], Ilya L. Mitushin[1], Daniel Munblit[4,9], Anzhelika Nargizyan[7], Yanina O. Orlova[1], Ismail M Osmanov[3,5], Anastasia S. Polyakova[1], Olga Romanova[4], Elmira Samitova[3,8], Anna Sologub[7], Ekaterina Spiridonova[4], Rustem F. Tepaev[1,2], Anna A. Tkacheva[1], Valeriya Yusupova[7], Elena Zholobova[10]
[1] *Infectious diseases hospital for children with COVID, National Medical Research Center for Children's Health, Moscow, Russia*
[2] *Department of Pediatrics and Pediatric Rheumatology, Institute of Child's Health, Sechenov First Moscow State Medical University (Sechenov University), Moscow, Russia*
[3] *ZA Bashlyaeva Children's Municipal Clinical Hospital, Moscow, Russia*
[4] *Department of Pediatrics and Pediatric Infectious Diseases, Institute of Child's Health, Sechenov First Moscow State Medical University (Sechenov University), Moscow, Russia*
[5] *Pirogov Russian National Research Medical University, Moscow, Russia*
[6] *Research Institute for Healthcare Organization and Medical Management of Moscow Healthcare Department, Moscow, Russia*
[7] *Morozov Children's Municipal Clinical Hospital of the Moscow City Health Department, Moscow, Russia*
[8] *Russian Medical Academy of Continuous Professional Education of the Ministry of Healthcare of the Russian Federation, Moscow, Russia*
[9] *Inflammation, Repair and Development Section, National Heart and Lung Institute, Faculty of Medicine, Imperial College London, London, United Kingdom*
[10] *Department of Children Diseases, Institute of Child's Health, Sechenov First Moscow State Medical University (Sechenov University), Moscow, Russia*

SPAIN
Carlos Daniel Grasa[1], Nuria Lopez Segura[2], Federico Martinon-Torres[3], Susana Melendo[4], Ana Mendez Echevarria[1], Juan Miguel Mesa Guzmán[8,9], Jorge Roberto Palacios Argueta[6], Irene Rivero-Calle[3], Jacques Rivière[4], Moisés Rodríguez-González[7], Pablo Rojo[5], Judith Sanchez Manubens[6], Pere Soler-Palacin[4], Antoni Soriano-Arandes[4], Alfredo Tagarro[5], Serena Villaverde[5]
[1] *Department of Pediatric Infectious Diseases, Hospital Universitario La Paz, Madrid, 28046, Madrid, Spain*
[2] *Hospital del Mar, Pediatrics, Spain*
[3] *Translational Pediatrics and Infectious Diseases Department. Hospital Clínico Universitario de Santiago de Compostela. Av. Choupana, 15706 Santiago de Compostela, Spain*
[4] *Pediatric Infectious Diseases and Immunodeficiencies Unit, Hospital Universitario Vall d'Hebron, Barcelona, Catalonia, Spain*

[5] *Pediatric Research and Clinical Trials Unit (UPIC), Instituto de Investigación Sanitaria Hospital 12 de Octubre (IMAS12), Spain*
[6] *Pediatric Cardiology Unit, Pediatric Medicine Service, Consorcio Sanitario Parc Tauli, Universitat Autonoma de Barcelona, Sabadell, Spain*
[7] *Department of Pediatric Cardiology, Puerta del Mar University Hospital, Cadiz, Spain*
[8] *Hospital Infanta Sofía, Pediatrics Department, San Sebastian de los Reyes, Madrid, Spain*
[9] *Fundación para la Investigación e Innovación Biomédica de los Hospitales Infanta Sofía y Alcalá de Henares, Madrid, Spain*

SWEDEN
Maria Altman[1], Petter Brodin[2], AnnaCarin Horne[3], Karin Palmblad[3]
[1] *Clinical Epidemiology Division, Department of Medicine Solna, Karolinska University Hospital, Karolinska Institutet, Stockholm, Sweden*
[2] *Science for Life Laboratory, Department of Women's and Children's Health, Karolinska Institutet, 17165, Solna, Sweden*
[3] *Unit of Pediatric Rheumatology, Department of Women's and Children's Health, Karolinska Institutet, Karolinska University Hospital, Solna, 171 76, Stockholm, Sweden*

SWITZERLAND
Barbara Brotschi[1], Patrick Meyer Sauteur[1], Jana Pachlopnik Schmid[1], Seraina Prader[1], Christa Relly[1], Luregn J. Schlapbach[1], Michelle Seiler[1], Johannes Trück[1], Daniela Wütz[1]
[1] *University Children's Hospital Zurich and Children's Research Center, University of Zurich (UZH), Switzerland*

THE NETHERLANDS
Naomi Ketharanathan[1], Clementien Vermont[1]
[1] *Department of Pediatric Infectious Diseases and Immunology, Erasmus MC-Sophia Children's Hospital, Rotterdam, The Netherlands*

TURKEY
Esra Akyüz Özkan[1], Emine Hafize Erdeniz[1]
[1] *Ondokuz Mayıs University Medical Faculty, Department of Pediatrics, Samsun, Turkey*

UKRAINE
Galina Borisova[3], Lidiya Boychenko[3], Nadiia Diudenko[3], Olexandr Kasiyan[2], Kostiantyn Katerynych[4], Kateryna Melnyk[3], Nelia Miagka[3], Maria Teslenko[4], Mykola Trykosh[2], Alla Volokha[1]
[1] *Shupyk National Medical Academy of Postgraduate Education, Kyiv, Ukraine*
[2] *Kyiv City Children’s Clinical Hospital N°1, Kyiv, Ukraine*
[3] *Kyiv City Children’s Clinical Hospital N°2, Kyiv, Ukraine*
[4] *Medical Centre Dobrobut, Kyiv, Ukraine*

UNITED KINGDOM
Toju Akomolafe[1], Eslam Al-Abadi[17], Nele Alders[2], Paula Avram[19], Alasdair Bamford[2], Millie Banks[1], Robin Basu Roy[1], Thomas Beattie[3], Olga Boleti[1], Jonathan Broad[3], Enitan D. Carrol[20], Michael Carter[3], Anchit Chandran[1], Hannah Cooper[18], Patrick Davies[16], Marieke Emonts[5,6], Ceri Evans[7], Katy Fidler[15], Caroline Foster[1], Chen Gong[3], Berin Gongrun[1], Carmen Gonzalez[19], Louis Grandjean[2], Karlie Grant[2], Yael Hacohen[2], Jack Hall[1], Jane Hassell[2], Christine Hesketh[8], Jessica Hewlett[3], Ahmad Hnieno[1], Hannah Holt-Davis[9], Aleena Hossain[1], Lee D Hudson[2], Mae Johnson[2], Sarah Johnson[1], Deepthi Jyothish[18], Beate Kampmann[10], Akhila Kavirayani[11], Deborah Kelly[12], Filip Kucera[2], Daniel Langer[8], Jon Lillie[13], Katherine Longbottom[1], Hermione Lyall[1], Niamh Mackdermott[1], Sarah Maltby[19], Thomas Mclelland[18], Anne-Marie McMahon[19], Danielle Miller[11], Zoe Morrison[12], Karyn Moshal[2], Jennifer Muller[12], Evangelia Myttaraki[1], Simon Nadel[1], Daniella Osaghae[1], Fatima Osman[1], Anna Ostrzewska[1], Mrinalini Panthula[1], Eleni Papachatzi[1],

Charalampia Papadopoulou[2], Harsita Patel[1], Justin Penner[2], Shervin Polandi[1], Andrew J. Prendergast[7], Padmanabhan Ramnarayan[2], Sophie Rhys-Evans[1], Andrew Riordan[4], Charlene M.C. Rodrigues[2], Sam Romaine[20], James Seddon[1], Delane Shingadia[2], Anand Srivastava[14], Siske Struik[12], Alice Taylor[2], Amanda Taylor[1], Andrew Taylor[18], Steven Tran[1], Gareth Tudor-Williams[1], Fabian van der Velden[5,6], Lyn Ventilacion[13], Paul A Wellman[3], Michael P. Yanney[14], Shunmay Yeung[1]

[1] *Department of Pediatric Infectious Diseases, Imperial College Healthcare NHS Trust, London, W2 1NY, UK*
[2] *Great Ormond Street Hospital for Children NHS Foundation Trust, London, WC1N 3JH, UK*
[3] *Department of Women and Children's Health, School of Life Course Sciences, King's College London, St Thomas' Hospital, SE1 7EH, London, UK*
[4] *Alder Hey Children's hospital, Pediatric Infectious Diseases, Liverpool, UK*
[5] *Pediatric Immunology, Infectious Diseases and Allergy, Great North Children's Hospital, Newcastle upon Tyne Hospitals NHS Foundation Trust, Queen Victoria Road NE1 4LP, Newcastle upon Tyne, UK*
[6] *Translational and Clinical Research Institute, Newcastle University, Newcastle upon Tyne, UK*
[7] *Department of Pediatric Infectious Diseases, Royal London Hospital, Barts Health NHS Trust, London E1 1BB, UK and Blizard Institute, Queen Mary University of London, London E1 2AT, UK*
[8] *Pediatric Emergency Department, St Helier Hospital, Wrythe Lane, Carshalton, Surrey, SM5 1AA, UK*
[9] *Pediatric Department, West Cumberland Hospital, Whitehaven, CA28 8JG, UK*
[10] *Faculty of Infectious and Tropical Disease, London School of Hygiene and Tropical Medicine, London, WC1E 7HT, UK*
[11] *Department of Pediatric Rheumatology, Oxford University Hospitals NHS Foundation Trust, Oxford, OX3 7HE, UK*
[12] *Children and Young Adult's Research Unit, Noah's Ark Children's Hospital for Wales, Heath Park, Cardiff, CF14 4XW, UK*
[13] *Pediatric Department, Lister Hospital, East and North Hertfordshire Hospital NHS Trust, Stevenage, SG1 4AB, UK*
[14] *Department of Pediatrics, Sherwood Forest Hospitals NHS Foundation Trust, UK*
[15] *Department of Pediatrics, Royal Alexandra Children's Hospital, Eastern Road, Brighton East Sussex BN2 5BE, UK*
[16] *Pediatric Critical Care Unit, Nottingham University Hospitals NHS Trust, Nottingham, UK*
[17] *Childhood Arthritis and Rheumatic Diseases Unit, Birmingham Women's and Children's Hospital NHS FT, Birmingham, B4 6NH, UK*
[18] *Department of General Pediatrics, Birmingham Women's and Children's NHS Foundation Trust, Birmingham, B4 6NH, UK*
[19] *Pediatric Rheumatology Department, Sheffield Children's Hospital Western Bank, Sheffield, S10 2TH, UK*
[20] *Institute of Infection Veterinary and Ecological Sciences, University of Liverpool, Liverpool, UK*

UNITED STATES OF AMERICA

Aditya Badheka[1], Sarah Badran[2], Dwight M. Bailey[3], Anna Kathryn Burch[4], Jane C. Burns[5], Catherine Cichon[5], Blake Cirks[6], Michael D. Dallman[7], Dennis R. Delany[8], Mary Fairchok[9], Samantha Friedman[10], Jennifer Geracht[6], Allison Langs-Barlow[9], Kelly Mann[9], Amruta Padhye[10], Alexis Quade[5], Kacy Alyne Ramirez[14], John Rockett[10], Imran Ali Sayed[11], Amr A. Shahin[12], Adriana Tremoulet[5], Samuel Umaru[13], Rebecca Widener[4]

[1] *Department of Pediatrics, University of Iowa Stead Family Children's Hospital, Iowa City, IA, 52242, USA*
[2] *Cardiology, Children's Hospital Los Angeles, Los Angeles, CA, 90027, USA*
[3] *Division of Pediatric Critical Care Medicine, Levine Children's Hospital, Atrium Health, Charlotte, NC, USA*
[4] *Division of Infectious Disease, Department of Pediatrics. University of South Carolina School of Medicine, Prisma Health Children's Hospital - Midlands. Columbia, SC, 29203, USA*
[5] *Department of Pediatrics, Rady Children's Hospital - San Diego, 3020 Children's Way, San Diego, CA 92123, USA*
[6] *Walter Reed National Military Medical Center, 8901 Wisconsin Avenue Bethesda Maryland, 20889-0001, USA*
[7] *University of South Carolina School of Medicine, Department of Pediatrics, Division of Critical Care Medicine, Prisma Health Children's Hospital – Midlands, Columbia, SC 29203, USA*

[8] *Department of Pediatric Cardiology, Medical University of South Carolina, 10 McClennan Banks Drive, Charleston, SC, USA*
[9] *Department of Pediatric Infectious Diseases M, Mary Bridge Children's Hospital Tacoma, WA, USA*
[10] *Pediatric Critical Care Division, University of Missouri Health Care, 400 N. Keene St., Columbia, Missouri, USA*
[11] *Department of Pediatric Critical Care Medicine, University of Colorado, Children's Hospital of Colorado at Colorado Springs, USA*
[12] *Department of pediatric intensive care, Tucson Medical Center, Tucson, Arizona, USA*
[13] *Pediatric Critical Care, Lehigh Valley Reilly Children's Hospital, Allentown, Pennsylvania, USA*
[14] *Wake Forest Baptist Medical Center, Medical Center Blvd, Meads Hall, 3rd Floor, Department of Pediatrics Winston-Salem, North Carolina, 27157, USA*

ZIMBABWE

Mujuru Hilda Angela[1], Gwendoline Kandawasvika[1]
[1] *University of Zimbabwe, College of Health Sciences, Child and Adolescent Health Unit, Box A 168, Avondale, Harare, Zimbabwe*

Supplementary Methods

Patient Recruitment

BATS invited recruitment of children with a wide, inclusive definition of MIS-C. The instructions to participating centres, including the various definitions in use, are contained in the 'BATS handbook' which is available as supplementary material.

The protocol and study information were translated into Spanish by Gabriela Ivankovich-Escoto and Rolando Ulloa-Gutierrez and into Portuguese by Rolando Andres Paternina-de la Ossa. Enrolment at individual study sites was undertaken by local investigators. The statistical group (listed in Consortium Membership) wrote the statistical analysis plan.

Data preparation

Data were entered in RedCap version 6.14.2. All subsequent processing and analysis were undertaken in R version 4.0.2. The included patients were finalized on 24 February 2021, with data changes restricted to correction of errors and missing data. Validation and correction of admission, discharge and immunomodulatory treatment dates was undertaken. Data were processed such that repeated clinical, laboratory and treatment variables were represented in a table with one row per patient-day.

Clinicians included the patients on their judgement of the patient meeting one or more of the international definitions for MIS-C.[1–3] Patients were excluded from analysis if an admission date was unavailable, data was not entered on the treatment form, there was no daily data and no discharge date, or the date of first immunomodulatory treatment was unclear. Only patients treated on or after the day of admission or transfer could contribute adjusted outcomes. Unadjusted death and complication rates were reported on all included patients.

Level of care variables, including respiratory support and inotropes, and the clinical variable fever were interpolated for missing daily data where preceding and following values were identical. Where missing data for respiratory support and inotropes followed a final value, if the final value indicated no support was needed, subsequent daily values were considered to be the same. Further, where total number of days of invasive ventilation, non-invasive ventilation, oxygen and inotropic support were available, missing data was entered assuming no discontinuous periods of treatment (supported by a low frequency of multiple episodes of inotropes, ventilation or oxygen usage in complete data).

Where multiple hospitals within one location reported patients, we inspected plots of admissions and ages to identify possible adjoining admissions. Two pairs of admissions in London were identified likely corresponding to the same patient based on age, gender, weight, admission periods and compatible laboratory and clinical variables. The data were merged and original records excluded.

Each site reported laboratory variables in units prespecified in the data collection tool, or with alternative units. Conversion to the same units was undertaken. Manual inspection of result distributions from individual sites was undertaken to identify and correct incorrect or discrepant units. Extreme outliers were inspected on a per individual basis and corrected when the value was discrepant with the rest of the biomarker time course.

For each day of admission, clinical severity was assessed on an ordinal scale:

1. Ventilated (invasive or non-invasive) and on inotropic support
2. Ventilated (invasive or non-invasive)
3. Inotropic support

4. Receiving oxygen
5. No supportive therapy last CRP ≥ 50
6. No supportive therapy last CRP < 50
7. Discharged

(Items 5–6 bracketed: No supportive therapy CRP unknown)

Additional levels were added for graphical presentation: death, ECMO and transferred (Figure S9B). This ordinal scale was developed by clinical consensus because there are no existing clinical severity scales for this condition. It would be inappropriate to use scales intended for acute COVID-19, which is initially a respiratory illness progressing to systemic disease, whereas MIS-C is a systemic illness with cardiovascular compromise predominating, and secondary respiratory compromise. Our scale considers escalating levels of clinical support, and in those not on support differentiates by level of CRP and admission status. This accords with clinical priorities when caring for patients: for those receiving organ support, coming off support is a key sign of improvement. For those not receiving organ support, improvement in inflammation is very important, and following that being fit for discharge.

Age was recorded in years and additional months. Where additional months were missing, they were assumed to be zero. Age in years was always present.

Patients' weight-for-age Z scores were calculated from the WHO reference data using the UK Royal College of Paediatrics and Child Health (RCPCH) Growth API[4]. The World Bank lending group classification was used for economic status.

Significant past medical history was recorded as primary or secondary immunodeficiency, HIV, autoimmune disease, chronic lung disease, chronic neurological disorder or malignancy.

Primary outcome definitions:

Inotropic support, ventilation and death (dichotomous)
Inotropic support and ventilation (invasive or non-invasive) at any time from the second day post-treatment, or death at any time. Inotropic support and ventilation were regarded as not available if the patient was transferred or died on day one or two, without report of support being received on day 2. If the patient was discharged on day 1 or 2, the outcome was regarded as negative. Death was regarded as missing for all patients transferred to other hospitals, and as negative for all patients whose destination was not recorded.

Improvement at day 2 (dichotomous)
Improvement at day 2 was reported relative to day 0 for:

- Any patient who was discharged on or before day 2
- Patients stepped down from ventilation or inotropic support
- Patients not ventilated or on inotropes who stepped down from oxygen
- Patients not receiving organ support whose CRP fell from above 50 mg/l on or before the day of treatment to below 50 mg/l.

Improvement was regarded as unknown if a patient was transferred on or before day 2, and negative for a patient who died on or before day 2.

Sensitivity analysis

- One planned sensitivity analyses was undertaken: Defining primary treatment as first treatments over two consecutive days (day 0-1)

Subgroup analysis
One planned subgroup analysis was undertaken (referred to as a sensitivity analysis in the protocol and statistical analysis plan):

- Patients fully meeting the WHO criteria for MIS-C

Secondary outcomes:

Failure of primary treatment

Defined as the addition of any immunomodulator from the first day after primary treatment. For patients receiving glucocorticoids within primary treatment, an escalation of more than 5 mg/kg prednisolone equivalent in total daily dose was required for further glucocorticoid usage to class as failure. If transferred before the fifth day following primary treatment, failure was regarded as not available.

Time to improvement in clinical severity

For each patient the time to improvement in clinical severity was calculated as:

- Time to come off ventilator or inotropes for patients receiving both therapies
- Time to come off ventilator for patients ventilated
- Time to come off inotropes for patients receiving inotropes
- Time to come off oxygen for patients receiving oxygen
- Time for CRP to fall below 50 mg/l for patients with final CRP on day of treatment or earlier of greater than or equal to 50 mg/l
- Time until discharge for all patients where preceding other event

Death / Inotropic support / Ventilation

As defined in composite primary outcome.

Fever

Presence of fever at any point from day 2. If no fever reported, but missing data, outcome regarded as not available.

Increase in level of support:

This was based on any commencement of:

- ECMO for patients not on ECMO on day 0
- Ventilation for patients not ventilated on day 0
- Inotropic support for patients not ventilated on day 0
- Oxygen for patients not on oxygen on day 0

Where none of the above led to classification of deterioration, death was regarded as deterioration and transfer was regarded as the outcome being unavailable. Patients discharged home or with unreported discharge destination were regarded as not having increased support.

Persisting coronary artery dilatation

The presence of a coronary artery with Lopez z-score ≥ 2.5 or a report of aneurysm without z-score on the final echocardiogram, undertaken on the second or subsequent days following treatment.[5] Regarded as not available if no echocardiogram reported, and negative if echocardiogram reported with no aneurysm or z-score ≥ 2.5.

Inflammatory markers and troponin

Time courses of CRP, ferritin and troponin were analysed for each treatment group.

Complications of drug therapy

Complications deemed to be the result of immunomodulatory treatment, including but not limited to: allergy/anaphylaxis, cataracts, gastric perforation, gastric ulceration, hip necrosis, hyperglycaemia, hyperlactataemia, opportunistic infection, profound bradycardia, psychosis and glucocorticoid-induced hypertension.

Left ventricular dysfunction

The presence of left ventricular dysfunction on any echocardiogram from the second day after commencement of primary immunomodulatory treatment. For this analysis, the presence of left

ventricular dysfunction prior to starting immunomodulatory treatment was added as an additional covariate for calculation of propensity scores (see below) to control for confounding due to differences in the prevalence of left ventricular dysfunction prior to treatment in each of the treatment arms.

Analysis

Confounding

All primary outcomes, sensitivity analyses, and secondary outcomes (excluding drug complications) underwent analysis following unstandardized inverse probability weighting by multinomial covariate-balanced propensity scores to control for baseline confounding factors, as implemented by WeightIt version 0.11.0, using the "just-identified" approach. The Average Treatment Effect (ATE) was estimated, except when comparing inflammatory markers between treated and untreated patients, when the Average Treatment Effect in the Untreated (ATU) was calculated (equivalent to the Average Treatment Effect in the Treated with the untreated group as the reference. In this way, treated patients were weighted to ensure covariates balanced with the untreated patients).

The analysis plan detailed the following variables could be considered for balancing:

1. Transfer vs. admission (dichotomous)
2. Treated in referring hospital (dichotomous)
3. Age (continuous)
4. Sex (binary)
5. Weight-for-age z-score greater than 2 (binary with missingness indicator)
6. Significant comorbidity (binary)
7. Days since fever at admission (continuous with missingness indicator)
8. Days of admission at treatment (continuous)
9. Total number of important clinical features reported up to day 0 (continuous)
10. COVID status: PCR positive, serology positive (if not PCR positive) or no positive result
11. Peak clinical severity to day of treatment (categorical)
12. Direction of change in clinical severity at day of treatment: increasing, stable, decreasing or unavailable (categorical)
13. Peak CRP up to day of treatment (quartile, or missing)
14. Direction of change in CRP at day of treatment (increasing, decreasing or unavailable)
15. Peak troponin up to day of treatment (quartile, or missing)
16. Peak BNP up to day of treatment (quartile, or missing)
17. Peak D-dimer up to day of treatment (quartile, or missing)
18. Coronary artery status up to day of treatment: last Z score ≥ 2.5, last Z score < 2.5, or not available

This was rationalised based on data availability and likely importance as determinants of treatment and outcome. For example, pre-treatment peak BNP and troponin were available less often than D-dimers (58 and 24% vs 72% respectively), and change in inflammatory markers and clinical severity was more often unavailable. The reduced covariates comprise:

1. Age
2. Sex
3. Weight-for-age z-score greater than 2
4. Significant comorbidity
5. Days of fever at admission
6. Days of admission at treatment
7. Total number of clinical features reported up to day of treatment
8. COVID status
9. Peak clinical severity to day of treatment
10. Peak CRP to day of treatment

11. Peak D dimer to day of treatment

The World Bank resource group was also added based on the importance of resource level to treatment availability. Important covariates were added for certain secondary analyses. When comparing patients receiving and not receiving immunomodulator therapy, variables reporting features up to the day of treatment were replaced with corresponding variables on admission (variables 7, 9, 10 and 11), and days of admission at treatment was removed, due to the lack of a corresponding first treatment day for those not receiving any immunomodulator. Balancing was repeated for every analysis on the population providing the outcome. No imputation for missing outcome data was undertaken.

We aimed for absolute standardized mean differences of 0.1 in continuous variables, and below, and Kolmogorov-Smirnov distances of 0.1 and below. Love plots were used to examine the extent of imbalance and consider the potential impact. We tolerated some deviation since covariates were also included in generalized linear outcome models. Weight distributions and propensity model coefficients are presented in Figure S11 and Tables S8-11.

Models
Modelling approaches producing robust sandwich standard errors were used. Dichotomous outcomes were estimated using weighted generalized linear models (quasibinomial family) as implemented within the survey package, adding all covariates used in covariate balancing, to produce doubly-robust estimates.

Time to event analyses were undertaken using weighted Cox proportional hazards model[6] estimated average hazard ratios. Covariates were reduced to resource group, age, peak clinical severity and peak CRP to day of treatment for ventilation due to low numbers.

Hypothesis testing
P values for primary hypotheses are corrected for multiple hypothesis testing with the Bonferroni-Holm procedure. All other outcomes are presented with 95% confidence intervals alone.

Sensitivity analysis
E-values are presented for primary outcomes as per the method of vanDerWeele and Ding[7].

Clinical severity over time
Clinical severity over time was presented as proportional column charts from two days before treatment to 10 days after treatment. Only patients treated after day 1 of admission contributed severity data for preceding days. Small numbers of patients had missing clinical severity data (maximum 4% on any day). The charts are presented weighted by the covariate-balanced propensity score.

Baseline comparison of treatment groups
Blood results, the proportion of patients ventilated and on inotropes and clinical features of Kawasaki disease were compared across treatment groups at the point of starting the first immunomodulator treatment, or the day of admission for patients who did not receive immunomodulatory treatment.

Inflammatory markers and troponin
Inflammatory markers were plotted as percentages of the peak value, per patient, throughout the course of their admission for each treatment group. Line plots were weighted by covariate-balancing propensity scores as described in Confounding. Smoothed curves with confidence intervals were plotted using a generalized additive model (geom_smooth from the ggplot2 package in R). Comparisons were also made within each treatment group for age and for patients who fulfilled the 2017 AHA criteria for Kawasaki Disease. Patients whose treatment commenced on day 7 of admission or beyond were excluded, as time courses would principally

represent the natural course.

Supplementary Tables

Table S1| Details of additional treatments given by initial immunomodulatory therapy groups.

Initial immunomodulatory therapy	Number of patients	No additional treatment	Additional treatment	Details of specific additional treatment						Glucocorticoid duration median days (IQR)
				IVIG	Glucocorticoid	Anti-IL1	Anti-IL6	Anti-TNF	Other immunomodulator	
**IVIG**	217	103	114	25	99	0	4	9	1	4 (3-6.5)
**IVIG + Glucocorticoid**	197	158	39	18	0	0	11	6	0	5 (3-7.75)
**Glucocorticoid**	89	40	49	47	0	0	2	0	1	7 (4-10)

Table S2 | Unabridged clinical features, demographic information, and blood results for all patients included in analysis.
Descriptive table of demographic features, clinical features and blood markers on admission, and Kawasaki Disease features during admission. Patients were divided by treatment arm on day 0 (IVIG alone, glucocorticoid alone, IVIG+Glucocorticoid, no treatment, and other (any other treatment combination including biologicals)). SARS-CoV-2 PCR data refer to tests taken during admission. Missing data are given as raw values and (%) where applicable.
Abbreviations: Ab: Antibody; ALT: alanine aminotransferase; APTT: activated partial thromboplastin time; BCG: Bacillus Calmette–Guérin; BNP: brain natriuretic peptide; CRP: C-reactive protein; ECMO: extracorporeal membrane oxygenation; KD: Kawasaki Disease; LDH: lactate dehydrogenase; PCR: polymerase chain reaction; PT: prothrombin time, WBC: white blood cell count.

	Everyone (N=614)	IVIG (N=246)	Glucocorticoid (N=99)	IVIG and Glucocorticoid (N=208)	Other (N=22)	No treatment (N=39)
**⁺Age**	8.3 [4.2 - 12]	7.0 [3.7 - 11]	8.8 [5.0 - 12]	8.8 [4.6 - 12]	13 [9.5 - 15]	9.6 [4.4 - 13]
**^Proportion male at birth**	376 (61.2%)	157 (63.8%)	59 (59.6%)	127 (61.1%)	15 (68.2%)	18 (46.2%)
**^Overweight (age-adjusted z score ≥ 2)**	90 (14.7%)	28 (11.4%)	10 (10.1%)	45 (21.6%)	4 (18.2%)	3 (7.69%)
**^Ethnicity**						
White	310 (50.5%)	124 (50.4%)	64 (64.6%)	95 (45.7%)	9 (40.9%)	18 (46.2%)
Latino	112 (18.2%)	33 (13.4%)	11 (11.1%)	60 (28.8%)	5 (22.7%)	3 (7.69%)
Black	75 (12.2%)	30 (12.2%)	2 (2.02%)	33 (15.9%)	4 (18.2%)	6 (15.4%)

	Everyone (N=614)	IVIG (N=246)	Glucocorticoid (N=99)	IVIG and Glucocorticoid (N=208)	Other (N=22)	No treatment (N=39)
Asian	49 (7.98%)	28 (11.4%)	6 (6.06%)	8 (3.85%)	2 (9.09%)	5 (12.8%)
Other or not known	68 (11.1%)	31 (12.6%)	16 (16.2%)	12 (5.77%)	2 (9.09%)	7 (17.9%)
^**Significant comorbidity**	21 (3.42%)	5 (2.03%)	7 (7.07%)	5 (2.40%)	1 (4.55%)	3 (7.69%)
^**Home country economic status**						
High-income economies	474 (77.2%)	209 (85.0%)	49 (49.5%)	158 (76.0%)	21 (95.5%)	37 (94.9%)
Upper-middle income economies	105 (17.1%)	32 (13.0%)	34 (34.3%)	37 (17.8%)	0 (0%)	2 (5.13%)
Lower-middle income economies	35 (5.70%)	5 (2.03%)	16 (16.2%)	13 (6.25%)	1 (4.55%)	0 (0%)
^**SARS-CoV-2 PCR positive**						
Yes	133 (21.9%)	36 (14.8%)	26 (26.5%)	53 (26.0%)	8 (36.4%)	10 (26.3%)
Tested but negative	427 (70.5%)	186 (76.2%)	67 (68.4%)	135 (66.2%)	14 (63.6%)	25 (65.8%)
Not tested	46 (7.59%)	22 (9.02%)	5 (5.10%)	16 (7.84%)	0 (0%)	3 (7.89%)
Missing	8 (1.3%)	2 (0.8%)	1 (1.0%)	4 (1.9%)	0 (0%)	1 (2.6%)
^**SARS-CoV-2 Ab positive**						
Yes	424 (70.4%)	163 (67.6%)	68 (70.1%)	163 (80.3%)	16 (72.7%)	14 (35.9%)
Tested but negative	89 (14.8%)	49 (20.3%)	7 (7.22%)	16 (7.88%)	4 (18.2%)	13 (33.3%)
Not tested	89 (14.8%)	29 (12.0%)	22 (22.7%)	24 (11.8%)	2 (9.09%)	12 (30.8%)
Missing	12 (2.0%)	5 (2.0%)	2 (2.0%)	5 (2.4%)	0 (0%)	0 (0%)
^**At admission level of care**						
No support	424 (69.1%)	191 (77.6%)	65 (65.7%)	124 (59.6%)	12 (54.5%)	32 (82.1%)
Oxygen	52 (8.47%)	17 (6.91%)	14 (14.1%)	18 (8.65%)	2 (9.09%)	1 (2.56%)
Inotropes	73 (11.9%)	18 (7.32%)	11 (11.1%)	40 (19.2%)	2 (9.09%)	2 (5.13%)
Ventilation	9 (1.47%)	3 (1.22%)	1 (1.01%)	3 (1.44%)	0 (0%)	2 (5.13%)
Inotropes and ventilation or ECMO	56 (9.12%)	17 (6.91%)	8 (8.08%)	23 (11.1%)	6 (27.3%)	2 (5.13%)
^**Clinical features on admission**						
**Fever**	580 (94.5%)	237 (96.3%)	92 (92.9%)	196 (94.2%)	20 (90.9%)	35 (89.7%)
**Sore throat**	149 (27.9%)	62 (30.1%)	23 (25.6%)	50 (26.2%)	3 (17.6%)	11 (35.5%)
Missing	79 (12.9%)	40 (16.3%)	9 (9.1%)	17 (8.2%)	5 (22.7%)	8 (20.5%)
**Cough**	124 (21.8%)	49 (21.4%)	24 (25.3%)	38 (19.6%)	7 (35.0%)	6 (19.4%)
Missing	45 (7.3%)	17 (6.9%)	4 (4.0%)	14 (6.7%)	2 (9.1%)	8 (20.5%)

	Everyone (N=614)	IVIG (N=246)	Glucocorticoid (N=99)	IVIG and Glucocorticoid (N=208)	Other (N=22)	No treatment (N=39)
**Respiratory distress**	88 (15.3%)	29 (12.8%)	12 (12.5%)	36 (18.2%)	6 (28.6%)	5 (14.3%)
Missing	38 (6.2%)	20 (8.1%)	3 (3.0%)	10 (4.8%)	1 (4.5%)	4 (10.3%)
**Abdominal pain**	365 (63.7%)	142 (63.7%)	48 (51.6%)	138 (69.0%)	16 (72.7%)	21 (60.0%)
Missing	41 (6.7%)	23 (9.3%)	6 (6.1%)	8 (3.8%)	0 (0%)	4 (10.3%)
**Diarrhea**	281 (48.0%)	100 (43.3%)	36 (38.3%)	120 (58.8%)	7 (31.8%)	18 (51.4%)
Missing	28 (4.6%)	15 (6.1%)	5 (5.1%)	4 (1.9%)	0 (0%)	4 (10.3%)
**Vomiting**	324 (56.1%)	118 (52.0%)	43 (45.7%)	135 (66.5%)	13 (65.0%)	15 (44.1%)
Missing	36 (5.9%)	19 (7.7%)	5 (5.1%)	5 (2.4%)	2 (9.1%)	5 (12.8%)
**Headache**	164 (31.6%)	66 (33.3%)	22 (25.6%)	61 (33.2%)	7 (35.0%)	8 (25.8%)
Missing	95 (15.5%)	48 (19.5%)	13 (13.1%)	24 (11.5%)	2 (9.1%)	8 (20.5%)
**Encephalopathy**	19 (3.39%)	5 (2.30%)	4 (4.40%)	8 (4.02%)	2 (10.0%)	0 (0%)
Missing	54 (8.8%)	29 (11.8%)	8 (8.1%)	9 (4.3%)	2 (9.1%)	6 (15.4%)
**Irritability**	116 (20.9%)	39 (18.1%)	22 (23.9%)	47 (23.9%)	1 (5.56%)	7 (21.9%)
Missing	60 (9.8%)	31 (12.6%)	7 (7.1%)	11 (5.3%)	4 (18.2%)	7 (17.9%)
**Lethargy**	222 (39.4%)	89 (40.8%)	46 (47.9%)	64 (32.3%)	11 (55.0%)	12 (37.5%)
Missing	50 (8.1%)	28 (11.4%)	3 (3.0%)	10 (4.8%)	2 (9.1%)	7 (17.9%)
**^Kawasaki Disease features during admission**						
Rash	396 (64.5%)	177 (72.0%)	63 (63.6%)	126 (60.6%)	11 (50.0%)	19 (48.7%)
Oral mucosal changes	327 (53.3%)	152 (61.8%)	51 (51.5%)	110 (52.9%)	9 (40.9%)	5 (12.8%)
Conjunctival injection	365 (59.4%)	164 (66.7%)	52 (52.5%)	132 (63.5%)	8 (36.4%)	9 (23.1%)
Edema or erythema of extremities	233 (37.9%)	94 (38.2%)	35 (35.4%)	89 (42.8%)	9 (40.9%)	6 (15.4%)
Skin peeling	75 (12.2%)	36 (14.6%)	11 (11.1%)	26 (12.5%)	1 (4.55%)	1 (2.56%)
Lymphadenopathy	224 (36.5%)	101 (41.1%)	31 (31.3%)	77 (37.0%)	6 (27.3%)	9 (23.1%)
BCG reactivity	24 (3.91%)	11 (4.47%)	2 (2.02%)	10 (4.81%)	1 (4.55%)	0 (0%)
**+Blood results on admission**						
**WBC (10^9/L)**	10 [7.0 - 14]	9.9 [7.1 - 14]	10 [6.8 - 15]	9.9 [7.0 - 14]	10 [8.2 - 12]	12 [6.6 - 14]
Missing	46 (7.5%)	20 (8.1%)	10 (10.1%)	10 (4.8%)	2 (9.1%)	4 (10.3%)
**Neutrophils (10^9/L)**	7.5 [5.1 - 11]	7.1 [5.1 - 10]	8.0 [4.7 - 13]	7.5 [5.1 - 11]	8.4 [6.6 - 9.8]	8.9 [5.3 - 11]
Missing	85 (13.8%)	38 (15.4%)	21 (21.2%)	20 (9.6%)	2 (9.1%)	4 (10.3%)
**Lymphocytes (10^9/L)**	1.2 [0.74 - 1.9]	1.4 [0.80 - 2.2]	1.1 [0.76 - 1.7]	1.1 [0.70 - 1.7]	0.81 [0.48 - 1.5]	1.1 [0.78 - 2.3]

	**Everyone (N=614)**	**IVIG (N=246)**	**Glucocorticoid (N=99)**	**IVIG and Glucocorticoid (N=208)**	**Other (N=22)**	**No treatment (N=39)**
Missing	74 (12.1%)	35 (14.2%)	13 (13.1%)	20 (9.6%)	2 (9.1%)	4 (10.3%)
**Hemoglobin (g/L)**	120 [110 - 130]	120 [110 - 130]	120 [110 - 130]	110 [100 - 120]	110 [100 - 120]	120 [110 - 130]
Missing	39 (6.4%)	16 (6.5%)	11 (11.1%)	6 (2.9%)	2 (9.1%)	4 (10.3%)
**Platelets (10^9/L)**	180 [120 - 260]	190 [130 - 290]	160 [120 - 240]	180 [130 - 240]	150 [91 - 230]	250 [150 - 320]
Missing	47 (7.7%)	21 (8.5%)	11 (11.1%)	8 (3.8%)	2 (9.1%)	5 (12.8%)
**PT (sec)**	15 [13 - 17]	15 [13 - 17]	15 [13 - 17]	15 [13 - 17]	16 [14 - 23]	13 [12 - 15]
Missing	253 (41.2%)	111 (45.1%)	46 (46.5%)	66 (31.7%)	7 (31.8%)	23 (59.0%)
**APTT (sec)**	32 [28 - 37]	33 [29 - 38]	33 [29 - 38]	30 [26 - 36]	35 [30 - 37]	29 [26 - 35]
Missing	223 (36.3%)	97 (39.4%)	41 (41.4%)	59 (28.4%)	6 (27.3%)	20 (51.3%)
**Fibrinogen (g/L)**	5.6 [4.5 - 6.8]	5.6 [4.5 - 6.4]	5.6 [4.6 - 6.4]	5.8 [4.5 - 7.0]	5.7 [4.7 - 6.9]	5.9 [3.4 - 7.2]
Missing	243 (39.6%)	108 (43.9%)	35 (35.4%)	70 (33.7%)	7 (31.8%)	23 (59.0%)
**D Dimer (ng/mL)**	2200 [1000 - 4200]	2300 [1000 - 4400]	2200 [1000 - 4000]	2100 [980 - 4000]	2500 [1600 - 4700]	1300 [480 - 4500]
Missing	232 (37.8%)	108 (43.9%)	38 (38.4%)	59 (28.4%)	4 (18.2%)	23 (59.0%)
**Troponin (ng/L)**	42 [10 - 190]	18 [8.0 - 55]	50 [16 - 150]	50 [30 - 260]	200 [13 - 2900]	11 [7.3 - 120]
Missing	310 (50.5%)	126 (51.2%)	63 (63.6%)	90 (43.3%)	4 (18.2%)	27 (69.2%)
**BNP (ng/L)**	130 [35 - 650]	74 [20 - 400]	380 [86 - 750]	160 [65 - 820]	150 [68 - 430]	18 [12 - 75]
Missing	477 (77.7%)	191 (77.6%)	81 (81.8%)	156 (75.0%)	14 (63.6%)	35 (89.7%)
**CRP (mg/L)**	150 [90 - 230]	150 [82 - 210]	130 [50 - 250]	150 [90 - 250]	160 [120 - 260]	160 [67 - 200]
Missing	84 (13.7%)	33 (13.4%)	20 (20.2%)	25 (12.0%)	2 (9.1%)	4 (10.3%)
**Ferritin (ug/L)**	460 [230 - 860]	410 [200 - 620]	530 [230 - 1100]	560 [300 - 920]	640 [310 - 1300]	230 [140 - 330]
Missing	237 (38.6%)	93 (37.8%)	53 (53.5%)	69 (33.2%)	2 (9.1%)	20 (51.3%)
**LDH (U/L)**	340 [260 - 470]	350 [280 - 460]	320 [250 - 470]	330 [250 - 480]	290 [250 - 570]	400 [260 - 480]
Missing	302 (49.2%)	129 (52.4%)	51 (51.5%)	87 (41.8%)	8 (36.4%)	27 (69.2%)
**Creatinine (µmol/L)**	47 [36 - 66]	43 [34 - 55]	55 [44 - 71]	52 [36 - 73]	55 [44 - 86]	53 [37 - 66]
Missing	94 (15.3%)	44 (17.9%)	21 (21.2%)	21 (10.1%)	2 (9.1%)	6 (15.4%)
**ALT (U/L)**	29 [18 - 52]	28 [16 - 45]	32 [20 - 56]	32 [20 - 54]	44 [20 - 140]	27 [15 - 59]
Missing	122 (19.9%)	51 (20.7%)	29 (29.3%)	29 (13.9%)	5 (22.7%)	8 (20.5%)
**Albumin (g/L)**	33 [28 - 38]	34 [29 - 40]	31 [27 - 34]	32 [28 - 38]	32 [29 - 37]	34 [30 - 39]
Missing	177 (28.8%)	72 (29.3%)	41 (41.4%)	47 (22.6%)	3 (13.6%)	14 (35.9%)

^Clinical and demographic features given as raw values and (%).

Table S3 | Clinical features, demographic information, and blood results for all patients included in analysis and subgroups meeting more restricted criteria

Descriptive table of demographic features, clinical features on admission, Kawasaki Disease features during admission, and blood markers on admission. All patients included in the analysis were classified as “Clinician diagnosed MIS-C”. This population was subdivided by those who met the full WHO MIS-C criteria, those who met full WHO MIS-C criteria with presence of bacteremia or toxic shock syndrome, and those who were missing one or more mandatory criteria (fever >3 days; 2 of more of rash/non-purulent conjunctivitis, or mucocutaneous signs/hypotension or shock/features of myocardial dysfunction/evidence of coagulopathy/acute gastrointestinal symptoms; elevated markers of inflammation; evidence of Covid-19). All “Clinician diagnosed MIS-C” cases were further divided by patients that met the definition of Kawasaki Disease as set out by the American Heart Association (persistent fever, and at least 4 of the 5 following mucocutaneous features: erythema and cracking lips; strawberry tongue, and/or erythema of oral and pharyngeal mucosa; bilateral non-purulent conjunctivitis; rash; erythema and edema of the hands and feet and/or skin peeling; and lymphadenopathy). Patients with coronary artery aneurysms were also classified as Kawasaki Disease, even if they did not have at least 4 mucocutaneous features. Atypical KD was defined as patients with persistent fever, CRP >30, and meeting at least 2 or 3 mucocutaneous features. 37 % of all patients included in the analysis met the definition of KD by American Heart Association guidelines[8]. SARS-CoV-2 PCR data refer to tests taken during admission.

Abbreviations: ALT: alanine aminotransferase; APTT: activated partial thromboplastin time; BCG: Bacillus Calmette–Guérin; BNP: brain natriuretic peptide; KD: Kawasaki Disease; LDH: lactate dehydrogenase; PCR: polymerase chain reaction; PT: prothrombin time, TSS: toxic shock syndrome; WBC: white blood cell count.

	**Clinician diagnosed MIS-C matched on WHO criteria**					**All patients with clinician diagnosed MIS-C**		
	**Overall (N=614)**	**MIS-C (full WHO criteria) (N=490)**	**MIS-C with bacteremia or TSS (N=7)**	**MIS-C missing 1 other criterion (N=92)**	**MIS-C missing >1 criteria (N=25)**	**KD (N=225)**	**Atypical KD (N=186)**	**Not KD (N=203)**
**+Age (years)**	8.3 [4.2 - 12]	8.6 [4.7 - 12]	9.9 [5.6 - 12]	7.2 [4.1 - 11]	4.1 [1.2 - 8.9]	6.5 [3.3 - 10]	8.8 [4.7 - 12]	10 [5.5 - 14]
**^Proportion male at birth**	376 (61.2%)	307 (62.7%)	4 (57.1%)	49 (53.3%)	16 (64.0%)	141 (62.7%)	113 (60.8%)	122 (60.1%)
**^Overweight (age-adjusted z score ≥ 2)**	90 (14.7%)	82 (16.7%)	1 (14.3%)	7 (7.61%)	0 (0%)	30 (13.3%)	27 (14.5%)	33 (16.3%)
**^Ethnicity**								
White	310 (50.5%)	239 (48.8%)	4 (57.1%)	52 (56.5%)	15 (60.0%)	110 (48.9%)	99 (53.2%)	101 (49.8%)
Latino	112 (18.2%)	102 (20.8%)	1 (14.3%)	8 (8.70%)	1 (4.00%)	42 (18.7%)	34 (18.3%)	36 (17.7%)
Black	75 (12.2%)	65 (13.3%)	2 (28.6%)	5 (5.43%)	3 (12.0%)	24 (10.7%)	21 (11.3%)	30 (14.8%)
Asian	49 (7.98%)	38 (7.76%)	0 (0%)	7 (7.61%)	4 (16.0%)	21 (9.33%)	16 (8.60%)	12 (5.91%)
Other or not known	68 (11.1%)	46 (9.39%)	0 (0%)	20 (21.7%)	2 (8.00%)	28 (12.4%)	16 (8.60%)	24 (11.8%)

	Clinician diagnosed MIS-C matched on WHO criteria					All patients with clinician diagnosed MIS-C		
	**Overall (N=614)**	**MIS-C (full WHO criteria) (N=490)**	**MIS-C with bacteremia or TSS (N=7)**	**MIS-C missing 1 other criterion (N=92)**	**MIS-C missing >1 criteria (N=25)**	**KD (N=225)**	**Atypical KD (N=186)**	**Not KD (N=203)**
^**Significant comorbidity**	21 (3.42%)	9 (1.84%)	1 (14.3%)	8 (8.70%)	3 (12.0%)	2 (0.889%)	4 (2.15%)	15 (7.39%)
^**Home country economic status**								
High-income economies	474 (77.2%)	366 (74.7%)	5 (71.4%)	82 (89.1%)	21 (84.0%)	164 (72.9%)	145 (78.0%)	165 (81.3%)
Upper-middle income economies	105 (17.1%)	94 (19.2%)	0 (0%)	8 (8.70%)	3 (12.0%)	49 (21.8%)	26 (14.0%)	30 (14.8%)
Lower-middle income economies	35 (5.70%)	30 (6.12%)	2 (28.6%)	2 (2.17%)	1 (4.00%)	12 (5.33%)	15 (8.06%)	8 (3.94%)
^**SARS-CoV-2 PCR positive**								
Yes	133 (21.9%)	113 (23.3%)	1 (14.3%)	13 (14.4%)	6 (25.0%)	39 (17.5%)	38 (20.8%)	56 (28.0%)
Tested but negative	427 (70.5%)	335 (69.1%)	6 (85.7%)	69 (76.7%)	17 (70.8%)	171 (76.7%)	125 (68.3%)	131 (65.5%)
Not tested	46 (7.59%)	37 (7.63%)	0 (0%)	8 (8.89%)	1 (4.17%)	13 (5.83%)	20 (10.9%)	13 (6.50%)
^**SARS-CoV-2 Ab positive**								
Yes	424 (70.4%)	390 (80.6%)	6 (85.7%)	23 (26.1%)	5 (21.7%)	168 (76.4%)	137 (74.9%)	119 (59.8%)
Tested but negative	89 (14.8%)	42 (8.68%)	1 (14.3%)	38 (43.2%)	8 (34.8%)	30 (13.6%)	27 (14.8%)	32 (16.1%)
Not tested	89 (14.8%)	52 (10.7%)	0 (0%)	27 (30.7%)	10 (43.5%)	22 (10.0%)	19 (10.4%)	48 (24.1%)
^**At admission level of care**								
No support	424 (69.1%)	327 (66.7%)	1 (14.3%)	74 (80.4%)	22 (88.0%)	159 (70.7%)	139 (74.7%)	126 (62.1%)
Oxygen	52 (8.47%)	41 (8.37%)	4 (57.1%)	7 (7.61%)	0 (0%)	14 (6.22%)	14 (7.53%)	24 (11.8%)
Inotropes	73 (11.9%)	68 (13.9%)	0 (0%)	5 (5.43%)	0 (0%)	28 (12.4%)	23 (12.4%)	22 (10.8%)
Ventilation	9 (1.47%)	6 (1.22%)	1 (14.3%)	0 (0%)	2 (8.00%)	4 (1.78%)	2 (1.08%)	3 (1.48%)
Inotropes and ventilation or ECMO	56 (9.12%)	48 (9.80%)	1 (14.3%)	6 (6.52%)	1 (4.00%)	20 (8.89%)	8 (4.30%)	28 (13.8%)
^**Clinical features on admission**								
Fever	580 (94.5%)	472 (96.3%)	7 (100%)	83 (90.2%)	18 (72.0%)	218 (96.9%)	181 (97.3%)	181 (89.2%)
Sore throat	149 (27.9%)	124 (28.0%)	3 (50.0%)	18 (26.1%)	4 (23.5%)	57 (28.2%)	59 (34.9%)	33 (20.1%)

	Clinician diagnosed MIS-C matched on WHO criteria					All patients with clinician diagnosed MIS-C		
	**Overall (N=614)**	**MIS-C (full WHO criteria) (N=490)**	**MIS-C with bacteremia or TSS (N=7)**	**MIS-C missing 1 other criterion (N=92)**	**MIS-C missing >1 criteria (N=25)**	**KD (N=225)**	**Atypical KD (N=186)**	**Not KD (N=203)**
Cough	124 (21.8%)	102 (22.0%)	2 (40.0%)	16 (19.5%)	4 (22.2%)	42 (19.4%)	39 (21.8%)	43 (24.9%)
Respiratory distress	88 (15.3%)	71 (15.4%)	3 (50.0%)	10 (11.2%)	4 (21.1%)	31 (14.4%)	21 (11.8%)	36 (19.8%)
Abdominal pain	365 (63.7%)	316 (67.7%)	2 (33.3%)	42 (50.0%)	5 (31.3%)	125 (59.8%)	125 (69.8%)	115 (62.2%)
Diarrhea	281 (48.0%)	243 (51.3%)	4 (57.1%)	30 (35.3%)	4 (20.0%)	91 (41.7%)	93 (52.0%)	97 (51.3%)
Vomiting	324 (56.1%)	275 (58.8%)	2 (33.3%)	43 (50.6%)	4 (21.1%)	126 (57.3%)	101 (57.1%)	97 (53.6%)
Headache	164 (31.6%)	140 (33.2%)	2 (50.0%)	20 (26.0%)	2 (12.5%)	54 (28.3%)	52 (32.5%)	58 (34.5%)
Encephalopathy	19 (3.39%)	17 (3.74%)	0 (0%)	0 (0%)	2 (10.5%)	9 (4.27%)	4 (2.35%)	6 (3.35%)
Irritability	116 (20.9%)	95 (21.1%)	1 (20.0%)	15 (19.5%)	5 (22.7%)	61 (28.5%)	28 (16.8%)	27 (15.6%)
Lethargy	222 (39.4%)	183 (39.8%)	2 (33.3%)	35 (43.2%)	2 (11.8%)	94 (42.9%)	70 (41.7%)	58 (32.8%)
**^Kawasaki Disease features during admission**								
Rash	396 (64.5%)	323 (65.9%)	5 (71.4%)	56 (60.9%)	12 (48.0%)	208 (92.4%)	134 (72.0%)	54 (26.6%)
Oral mucosal changes	327 (53.3%)	272 (55.5%)	3 (42.9%)	42 (45.7%)	10 (40.0%)	208 (92.4%)	97 (52.2%)	22 (10.8%)
Conjunctival injection	365 (59.4%)	305 (62.2%)	3 (42.9%)	46 (50.0%)	11 (44.0%)	202 (89.8%)	118 (63.4%)	45 (22.2%)
Edema or erythema of extremities	233 (37.9%)	199 (40.6%)	2 (28.6%)	23 (25.0%)	9 (36.0%)	143 (63.6%)	66 (35.5%)	24 (11.8%)
Skin peeling	75 (12.2%)	60 (12.2%)	1 (14.3%)	11 (12.0%)	3 (12.0%)	54 (24.0%)	13 (6.99%)	8 (3.94%)
Lymphadenopathy	224 (36.5%)	184 (37.6%)	1 (14.3%)	33 (35.9%)	6 (24.0%)	159 (70.7%)	50 (26.9%)	15 (7.39%)
BCG reactivity	24 (3.91%)	20 (4.08%)	0 (0%)	2 (2.17%)	2 (8.00%)	12 (5.33%)	8 (4.30%)	4 (1.97%)
**+Bloods on admission**								
WBC (10^9/L)	10 [7.0 - 14]	9.9 [7.0 - 14]	9.8 [4.7 - 13]	11 [7.6 - 16]	8.2 [5.0 - 13]	10 [7.1 - 14]	9.8 [7.3 - 14]	10 [6.5 - 15]
Neutrophils (10^9/L)	7.5 [5.1 - 11]	7.5 [5.1 - 11]	9.8 [5.2 - 12]	8.3 [5.5 - 12]	4.4 [2.7 - 8.7]	7.8 [5.1 - 11]	7.4 [5.4 - 11]	7.3 [4.2 - 12]
Lymphocytes (10^9/L)	1.2 [0.74 - 1.9]	1.1 [0.70 - 1.8]	1.3 [0.70 - 1.4]	1.4 [0.90 - 2.3]	2.6 [1.4 - 3.7]	1.3 [0.75 - 2.1]	1.2 [0.74 - 1.8]	1.2 [0.73 - 1.8]

	Clinician diagnosed MIS-C matched on WHO criteria					All patients with clinician diagnosed MIS-C		
	Overall (N=614)	MIS-C (full WHO criteria) (N=490)	MIS-C with bacteremia or TSS (N=7)	MIS-C missing 1 other criterion (N=92)	MIS-C missing >1 criteria (N=25)	KD (N=225)	Atypical KD (N=186)	Not KD (N=203)
Hemoglobin (g/L)	120 [110 - 130]	120 [100 - 130]	110 [99 - 110]	110 [110 - 130]	120 [110 - 120]	120 [100 - 120]	120 [110 - 130]	110 [110 - 130]
Platelets (10^9/L)	180 [120 - 260]	170 [120 - 240]	150 [140 - 170]	250 [170 - 340]	270 [230 - 370]	170 [120 - 240]	180 [120 - 260]	200 [140 - 280]
PT (sec)	15 [13 - 17]	15 [13 - 17]	16 [12 - 19]	15 [12 - 17]	14 [13 - 16]	15 [13 - 17]	15 [13 - 17]	15 [12 - 18]
APTT (sec)	32 [28 - 37]	32 [28 - 37]	34 [33 - 42]	34 [28 - 37]	31 [20 - 39]	33 [27 - 37]	32 [28 - 36]	33 [28 - 39]
Fibrinogen (g/L)	5.6 [4.5 - 6.8]	5.7 [4.6 - 6.8]	6.0 [4.5 - 7.0]	5.6 [4.0 - 6.9]	4.9 [4.6 - 5.4]	5.6 [4.7 - 6.7]	5.8 [4.6 - 6.9]	5.7 [4.2 - 6.8]
D Dimer (ng/mL)	2200 [1000 - 4200]	2300 [1000 - 4300]	4000 [2800 - 5200]	1700 [1000 - 3600]	980 [540 - 9100]	2200 [1000 - 4200]	2100 [1100 - 4200]	2200 [930 - 4500]
Troponin (ng/L)	42 [10 - 190]	46 [12 - 200]	48 [30 - 61]	22 [5.0 - 170]	4.0 [3.4 - 9.3]	42 [10 - 120]	27 [10 - 90]	51 [12 - 300]
BNP (ng/L)	130 [35 - 650]	150 [42 - 700]	2700 [1500 - 4000]	62 [19 - 150]	7.5 [6.3 - 8.8]	170 [33 - 660]	82 [36 - 480]	130 [43 - 660]
CRP (mg/L)	150 [90 - 230]	150 [90 - 230]	300 [200 - 410]	130 [62 - 210]	51 [12 - 120]	150 [90 - 220]	170 [110 - 250]	140 [61 - 220]
Ferritin (ug/L)	460 [230 - 860]	480 [250 - 900]	1400 [1100 - 1500]	320 [190 - 580]	160 [93 - 380]	530 [260 - 910]	440 [240 - 860]	420 [180 - 800]
LDH (U/L)	340 [260 - 470]	340 [260 - 470]	740 [710 - 880]	290 [240 - 380]	330 [310 - 450]	320 [250 - 440]	340 [260 - 490]	350 [280 - 510]
Creatinine (µmol/L)	47 [36 - 66]	48 [37 - 68]	93 [88 - 110]	40 [33 - 53]	41 [24 - 53]	43 [33 - 56]	50 [39 - 68]	53 [37 - 72]
ALT (U/L)	29 [18 - 52]	31 [19 - 54]	60 [31 - 100]	22 [11 - 45]	18 [14 - 31]	30 [19 - 54]	31 [19 - 52]	27 [17 - 51]
Albumin (g/L)	33 [28 - 38]	32 [28 - 38]	30 [29 - 33]	33 [28 - 39]	41 [35 - 43]	32 [28 - 37]	33 [29 - 39]	33 [28 - 39]

^Clinical and demographic features given as raw values and (%).
+Numerical values given as median values and [interquartile ranges].

Table S4 | Distribution of patients meeting WHO criteria subdivided by Kawasaki Disease status and initial treatment given.

Table showing patients matched on MIS-C WHO criteria and divided by whether they met the definition of Kawasaki Disease set out by the American Heart Association (persistent fever, and at least 4 of the 5 following mucocutaneous features: erythema and cracking lips; strawberry tongue, and/or erythema of oral and pharyngeal mucosa; bilateral non-purulent conjunctivitis; rash; erythema and edema of the hands and feet and/or skin peeling; and lymphadenopathy). Patients with coronary artery aneurysms were also classified as Kawasaki Disease, even if they did not have at least 4 mucocutaneous features. Atypical KD was defined as patients with persistent fever, CRP >30, and meeting at least 2 or 3 mucocutaneous features. These columns are compared with the primary treatments received on day 0, and whether they were under 6 or over 6. IVIG is used proportionately more in those meeting AHA criteria. Values given as raw values and (%). Abbreviations: KD: Kawasaki Disease; TSS: toxic shock syndrome.

	**MIS-C (full WHO criteria)**			**MIS-C with bacteremia or TSS**			**MIS-C missing 1 other criterion**			**MIS-C missing >1 criteria**			**All patients**		
	**KD (N=193)**	**Atypical KD (N=160)**	**Not KD (N=137)**	**KD (N=1)**	**Atypical KD (N=3)**	**Not KD (N=3)**	**KD (N=24)**	**Atypical KD (N=19)**	**Not KD (N=49)**	**KD (N=7)**	**Atypical KD (N=4)**	**Not KD (N=14)**	**KD (N=225)**	**Atypical KD (N=186)**	**Not KD (N=203)**
**Age (years)**															
Over 6	105 (54.4%)	120 (75.0%)	103 (75.2%)	1 (100%)	1 (33.3%)	3 (100%)	12 (50.0%)	8 (42.1%)	34 (69.4%)	2 (28.6%)	0 (0%)	7 (50.0%)	120 (53.3%)	129 (69.4%)	147 (72.4%)
Under 6	88 (45.6%)	40 (25.0%)	34 (24.8%)	0 (0%)	2 (66.7%)	0 (0%)	12 (50.0%)	11 (57.9%)	15 (30.6%)	5 (71.4%)	4 (100%)	7 (50.0%)	105 (46.7%)	57 (30.6%)	56 (27.6%)
**First immunomodulator given**															
IVIG	83 (43.0%)	61 (38.1%)	48 (35.0%)	0 (0%)	1 (33.3%)	0 (0%)	18 (75.0%)	12 (63.2%)	13 (26.5%)	5 (71.4%)	2 (50.0%)	3 (21.4%)	106 (47.1%)	76 (40.9%)	64 (31.5%)
Glucocorticoid	25 (13.0%)	29 (18.1%)	24 (17.5%)	0 (0%)	2 (66.7%)	1 (33.3%)	4 (16.7%)	1 (5.26%)	12 (24.5%)	1 (14.3%)	0 (0%)	0 (0%)	30 (13.3%)	32 (17.2%)	37 (18.2%)
IVIG and glucocorticoid	79 (40.9%)	56 (35.0%)	51 (37.2%)	1 (100%)	0 (0%)	0 (0%)	1 (4.17%)	3 (15.8%)	11 (22.4%)	1 (14.3%)	0 (0%)	5 (35.7%)	82 (36.4%)	59 (31.7%)	67 (33.0%)
Other	5 (2.59%)	6 (3.75%)	5 (3.65%)	0 (0%)	0 (0%)	0 (0%)	0 (0%)	0 (0%)	5 (10.2%)	0 (0%)	0 (0%)	1 (7.14%)	5 (2.22%)	6 (3.23%)	11 (5.42%)
No treatment	1 (0.518%)	8 (5.00%)	9 (6.57%)	0 (0%)	0 (0%)	2 (66.7%)	1 (4.17%)	3 (15.8%)	8 (16.3%)	0 (0%)	2 (50.0%)	5 (35.7%)	2 (0.889%)	13 (6.99%)	24 (11.8%)

Table S5 | Primary Outcomes and Sensitivity Analyses

Table showing the odds ratios and 95% confidence intervals for the primary outcomes in patients who received a combination of IVIG and glucocorticoids as primary treatment vs IVIG alone and patients who received glucocorticoids alone as primary treatment vs IVIG. Crude numbers are shown in the second column as the numerator/denominator for those providing the outcome, and the number where the outcome is unavailable in parentheses. Weighted proportions are also shown. The E-value for the strength of unmeasured confounding necessary to move a point estimate to the null value is shown for primary outcomes.

Primary Outcomes

	Raw and weighted proportions (missing)	Odds Ratio	Confidence Interval (95%)	Adj. p value	E-value
IVIG+Glucocorticoids vs IVIG					
On Inotropes/Ventilated D2+ or Death	IVIG: 44/211 (20.9%; 6 missing). Weighted: 31.2% IVIG+S: 56/180 (31.1%; 17 missing). Weighted: 28.1%	0.77	0.33 - 1.82	p=1	1.5
Improvement D2	IVIG: 54/191 (28.3%; 26 missing). Weighted: 29.6% IVIG+S: 54/166 (32.5%; 31 missing). Weighted: 28.3%	0.9	0.48 - 1.69	p=1	1.3
Glucocorticoids vs IVIG					
On Inotropes/Ventilated D2+ or Death	IVIG: 44/211 (20.9%; 6 missing). Weighted: 31.2% S: 17/83 (20.5%; 6 missing). Weighted: 26.4%	0.54	0.22 - 1.33	p=0.7	2.1
Improvement D2	IVIG: 54/191 (28.3%; 26 missing). Weighted: 29.6% S: 20/77 (26%; 12 missing). Weighted: 30.6%	0.93	0.43 - 2.04	p=1	1.2

Table S5 | Secondary Outcomes and Time to Event Analyses
A: Table showing the odds ratio and 95% confidence interval for each of the secondary outcomes for patients who received a combination of IVIG and glucocorticoids as primary treatment vs IVIG alone and patients who received glucocorticoids alone as primary treatment vs IVIG. Crude numbers for dichotomous outcomes are shown in the second column as the numerator/denominator for those providing the outcome, and the number where the outcome is unavailable in parentheses. Weighted proportions are also given.
B: Results of sensitivity analyses in subsets of patients that met the WHO MIS-C criteria and those where primary treatments were defined as first treatments over two consecutive days (day 0-1).
C: Table showing the average hazard ratios and 95% confidence intervals for time to stop inotropes, ventilation, oxygen and time to improvement for patients who received a combination of IVIG and glucocorticoids as primary treatment vs IVIG alone and patients who received glucocorticoids alone as primary treatment vs IVIG.

5A

Secondary Outcomes			
	Raw and weighted proportions (missing)	Odds Ratio	Confidence Interval (95%)
IVIG+Glucocorticoid vs IVIG			
Inotropes D2+	IVIG: 38/216 (17.6%; 1 missing). Weighted: 22.9% IVIG+S: 49/189 (25.9%; 8 missing). Weighted: 24.9%	1.43	0.57 - 3.62
Ventilation D2+	IVIG: 21/208 (10.1%; 9 missing). Weighted: 9% IVIG+S: 30/183 (16.4%; 14 missing). Weighted: 11.8%	1.1	0.39 - 3.09
Improvement D3	IVIG: 105/202 (52%; 15 missing). Weighted: 53.4% IVIG+S: 96/166 (57.8%; 31 missing). Weighted: 52.9%	0.97	0.54 - 1.73
Treatment Escalation	IVIG: 114/216 (52.8%; 1 missing). Weighted: 54.8% IVIG+S: 39/194 (20.1%; 3 missing). Weighted: 21.2%	0.18	0.10 - 0.33
Treatment Escalation D0/1	IVIG: 41/143 (28.7%; 2 missing). Weighted: 25.7% IVIG+S: 32/275 (11.6%; 4 missing). Weighted: 12.5%	0.33	0.16 - 0.71
Fever D2+	IVIG: 86/182 (47.3%; 35 missing). Weighted: 48% IVIG+S: 55/149 (36.9%; 48 missing). Weighted: 37.5%	0.6	0.31 - 1.17
Fever D3+	IVIG: 31/172 (18%; 45 missing). Weighted: 18.2% IVIG+S: 32/143 (22.4%; 54 missing). Weighted: 19.3%	1.1	0.46 - 2.63
Death	IVIG: 3/213 (1.4%; 4 missing). Weighted: 3.6% IVIG+S: 5/184 (2.7%; 13 missing). Weighted: 1.2%	0.32	0.05 - 1.86
Any Deterioration	IVIG: 23/213 (10.8%; 4 missing). Weighted: 10%	1.22	0.55 - 2.71

Secondary Outcomes			
	Raw and weighted proportions (missing)	Odds Ratio	Confidence Interval (95%)
	IVIG+S: 28/187 (15%; 10 missing). Weighted: 11.9%		
LV Dysfunction D2+	IVIG: 23/215 (10.7%; 2 missing). Weighted: 8.5% IVIG+S: 28/188 (14.9%; 9 missing). Weighted: 13.6%	1.65	0.78 - 3.49
Aneurysm	IVIG: 10/143 (7%; 74 missing). Weighted: 5.4% IVIG+S: 6/115 (5.2%; 82 missing). Weighted: 4.3%	0.32	0.03 - 3.21
Glucocorticoid vs IVIG			
Inotropes D2+	IVIG: 38/216 (17.6%; 1 missing). Weighted: 22.9% S: 16/87 (18.4%; 2 missing). Weighted: 23.6%	1.38	0.54 - 3.51
Ventilation D2+	IVIG: 21/208 (10.1%; 9 missing). Weighted: 9% S: 7/87 (8%; 2 missing). Weighted: 10.1%	0.83	0.28 - 2.49
Improvement D3	IVIG: 105/202 (52%; 15 missing). Weighted: 53.4% S: 34/78 (43.6%; 11 missing). Weighted: 50.3%	0.87	0.43 - 1.75
Treatment Escalation	IVIG: 114/216 (52.8%; 1 missing). Weighted: 54.8% S: 49/88 (55.7%; 1 missing). Weighted: 59.9%	1.31	0.64 - 2.68
Treatment Escalation D0/1	IVIG: 41/143 (28.7%; 2 missing). Weighted: 25.7% S: 19/58 (32.8%; 1 missing). Weighted: 32%	1.42	0.54 - 3.73
Fever D2+	IVIG: 86/182 (47.3%; 35 missing). Weighted: 48% S: 36/75 (48%; 14 missing). Weighted: 46.7%	0.95	0.43 - 2.09
Fever D3+	IVIG: 31/172 (18%; 45 missing). Weighted: 18.2% S: 25/71 (35.2%; 18 missing). Weighted: 31.4%	2.44	0.93 - 6.42
Death	IVIG: 3/213 (1.4%; 4 missing). Weighted: 3.6% S: 3/83 (3.6%; 6 missing). Weighted: 9%	2.64	0.36 - 19.58
Any Deterioration	IVIG: 23/213 (10.8%; 4 missing). Weighted: 10% S: 13/84 (15.5%; 5 missing). Weighted: 18.7%	1.92	0.79 - 4.67
LV Dysfunction D2+	IVIG: 23/215 (10.7%; 2 missing). Weighted: 8.5% S: 7/87 (8%; 2 missing). Weighted: 7.7%	0.85	0.24 - 2.96
Aneurysm	IVIG: 10/143 (7%; 74 missing). Weighted: 5.4%	0.95	0.24 - 3.74

Secondary Outcomes			
	Raw and weighted proportions (missing)	Odds Ratio	Confidence Interval (95%)
	S: 4/68 (5.9%; 21 missing). Weighted: 5.6%		

5B

Secondary Analyses - Patients who met WHO MIS-C criteria			
	Raw and weighted proportions (missing)	Odds Ratio	Confidence Interval (95%)
IVIG+Glucocorticoid vs IVIG			
On Inotropes/Ventilated D2+ or Death	IVIG: 40/169 (23.7%; 4 missing). Weighted: 30.2% IVIG+S: 54/162 (33.3%; 15 missing). Weighted: 28.6%	0.95	0.37 - 2.45
Improvement D2	IVIG: 43/152 (28.3%; 21 missing). Weighted: 26.8% IVIG+S: 52/152 (34.2%; 25 missing). Weighted: 28.4%	1.09	0.53 - 2.23
Glucocorticoid vs IVIG			
On Inotropes/Ventilated D2+ or Death	IVIG: 40/169 (23.7%; 4 missing). Weighted: 30.2% S: 12/68 (17.6%; 2 missing). Weighted: 19%	0.3	0.10 - 0.85
Improvement D2	IVIG: 43/152 (28.3%; 21 missing). Weighted: 26.8% S: 16/60 (26.7%; 10 missing). Weighted: 40.8%	1.95	0.83 - 4.60

Sensitivity Analyses - D0/1 as day of starting primary treatment			
	Raw and weighted proportions (missing)	Odds Ratio	Confidence Interval (95%)
IVIG+Glucocorticoid vs IVIG			
On Inotropes/Ventilated D2+ or Death	IVIG: 21/140 (15%; 5 missing). Weighted: 23.9% IVIG+S: 81/260 (31.2%; 19 missing). Weighted: 26.8%	1.27	0.51 - 3.12
Improvement D2	IVIG: 38/126 (30.2%; 19 missing). Weighted: 36.4% IVIG+S: 71/240 (29.6%; 39 missing). Weighted: 26.1%	0.54	0.27 - 1.05
Glucocorticoid vs IVIG			
On Inotropes/Ventilated D2+ or Death	IVIG: 21/140 (15%; 5 missing). Weighted: 23.9% S: 9/55 (16.4%; 4 missing). Weighted: 15.9%	0.33	0.09 - 1.14
Improvement D2	IVIG: 38/126 (30.2%; 19 missing). Weighted: 36.4% IVIG+S: 71/240 (29.6%; 39 missing). Weighted: 26.1%	1.01	0.41 - 2.48

5C

Time to Event Analyses			
	Groups and censoring	Average Hazard Ratio	Confidence Interval (95%)
IVIG+Glucocorticoid vs IVIG			
Time to stop inotropes^	IVIG: n=32; 3 censored. IVIG+S: n=62; 12 censored.	1.23	0.646 - 2.339
Time to stop ventilation^	IVIG: n=21; 9 censored. IVIG+S: n=24; 6 censored.	1.52	0.506 - 4.565
Time to stop oxygen^	IVIG: n=30; 3 censored. IVIG+S: n=51; 12 censored.	0.93	0.502 - 1.739
Time to improvement^	IVIG: n=216; 16 censored. IVIG+S: n=196; 21 censored.	0.89	0.665 - 1.185
Glucocorticoid vs IVIG			
Time to stop inotropes^	IVIG: n=32; 3 censored. S: n=18; 2 censored.	1.31	0.71 - 2.407
Time to stop ventilation^	IVIG: n=21; 9 censored. S: n=8; 3 censored.	1.41	0.375 - 5.288
Time to stop oxygen^	IVIG: n=30; 3 censored. S: n=22; 2 censored.	1.60	0.840 - 3.058
Time to improvement^	IVIG: n=216; 16 censored. S: n=89; 6 censored.	1.03	0.727 - 1.460

Table S6 | Coronary artery aneurysms by initial immunomodulatory therapy groups

Initial immunomodulatory therapy	Number of patients	Aneurysms pre-treatment	Aneurysms after treatment	Timing of last post-treatment echocardiogram median days (IQR)
**IVIG**	246	16/65 (181 unknown)	19/201 (45 unknown)	5 (2-8)
**IVIG+ Glucocorticoid**	208	16/77 (131 unknown)	11/149 (59 unknown)	5 (3-9)
**Glucocorticoid**	99	0/16 (83 unknown)	4/84 (15 unknown)	6 (3-10.25)
**No immunomodulator**	39	0/0 (39 unknown)	0/29 (10 unknown)	4 (3-7)
**Other treatment combination**	22	5/13 (9 unknown)	4/20 (2 unknown)	3.5 (2.75-5.25)

Table S7 | Treatment related complications
Treatment related complications reported by clinicians.

Treatment	Complication	Number of patients
**Glucocorticoid**	Profound Bradycardia	1
	Hyperglycaemia	7
	Glucocorticoid-induced hypertension	7
	Other complication not specified	1
	Total (% of patients receiving glucocorticoids)	16/411 (3.9%)
**IVIG**	Mild rash and lip swelling	1
	Other complication not specified	8
	Total (% of patients receiving IVIG)	9/508 (1.8%)
**Anakinra**	Superficial cutaneous skin reaction	1
**Anticoagulant**	Significant bleeding	1
	Mild bleeding	1
**ECMO**	Cerebrovascular accident	1
**Vancomycin**	Acute kidney injury	1

Table S8 | Coefficients for covariate-balancing propensity score multinomial model
Primary outcome: inotropes/ventilation day 2+ or death

J - statistic: 0.6427698 Log-Likelihood: -459.3349	Estimate	Std. Error	z value	Pr(>|z|)
**IVIG: (Intercept)**	9.1273488	3.2637292	2.7966012	0.0051643
**IVIG: baseline_resource_groupHigh.income.economies**	0.7105361	0.1346049	5.2786797	0.0000001
**IVIG: baseline_resource_groupLower.middle.income.economies**	-1.0793630	0.1487181	-7.2577802	0.0000000
**IVIG: baseline_age**	0.2885691	0.1686887	1.7106609	0.0871437
**IVIG: baseline_weight_z_over_2**	-0.6335239	0.1364810	-4.6418469	0.0000035
**IVIG: baseline_weight_z_missingTRUE**	-2.0015045	0.1335843	-14.9830823	0.0000000
**IVIG: baseline_sig_comorbidityYes**	-0.9945116	0.1752417	-5.6750842	0.0000000
**IVIG: baseline_fever_days_at_treating_admission**	0.2177913	0.1266418	1.7197427	0.0854792
**IVIG: baseline_fever_days_missingTRUE**	0.1188209	0.1207425	0.9840847	0.3250739
**IVIG: genderMale**	-0.1179749	0.1671981	-0.7055998	0.4804370
**IVIG: baseline_number_clin_feat**	0.0939253	0.1431293	0.6562270	0.5116781
**IVIG: baseline_admission_day_at_treatment**	-0.1173088	0.2014868	-0.5822160	0.5604212
**IVIG: baseline_covid_positivePCR**	-1.0568009	0.1546772	-6.8323006	0.0000000
**IVIG: baseline_covid_positiveSerology**	0.3088184	0.1410060	2.1901085	0.0285164
**IVIG: baseline_peak_levin_LOCNo.support.CRP...50**	-2.1685618	0.1811456	-11.9713769	0.0000000
**IVIG: baseline_peak_levin_LOCNo.support.CRP.unknown**	-0.4008751	0.2486952	-1.6119135	0.1069808
**IVIG: baseline_peak_levin_LOCNo.support.CRP....50**	-0.0170510	0.2727179	-0.0625227	0.9501466
**IVIG: baseline_peak_levin_LOCOxygen**	-0.1874003	0.2220194	-0.8440720	0.3986292
**IVIG: baseline_peak_levin_LOCInotropes**	1.1078531	0.2209830	5.0132943	0.0000005
**IVIG: baseline_peak_levin_LOCVentilation**	-1.2116638	0.1864877	-6.4972843	0.0000000
**IVIG: baseline_peak_CRP.75th**	-0.1412343	0.1602199	-0.8815024	0.3780459
**IVIG: baseline_peak_CRP25.50th**	-0.4762655	0.1584424	-3.0059229	0.0026478
**IVIG: baseline_peak_CRP50.75th**	0.3055584	0.1582154	1.9312814	0.0534483
**IVIG: baseline_peak_CRPMissing**	-0.5614239	0.2281199	-2.4610918	0.0138515
**IVIG: baseline_peak_ddimer.75th**	0.2302374	0.1987300	1.1585437	0.2466422
**IVIG: baseline_peak_ddimer25.50th**	0.2034261	0.1958822	1.0385126	0.2990315
**IVIG: baseline_peak_ddimer50.75th**	-0.7322349	0.1926503	-3.8008500	0.0001442
**IVIG: baseline_peak_ddimerMissing**	0.5034953	0.2207765	2.2805655	0.0225742
**IVIG+G: (Intercept)**	4.2697071	5.2837250	0.8080865	0.4190408
**IVIG+G: baseline_resource_groupHigh.income.economies**	2.5823048	0.2432365	10.6164361	0.0000000
**IVIG+G: baseline_resource_groupLower.middle.income.economies**	-1.6934589	0.2549718	-6.6417501	0.0000000
**IVIG+G: baseline_age**	0.9965438	0.3440187	2.8967718	0.0037702
**IVIG+G: baseline_weight_z_over_2**	0.8686548	0.1964212	4.4224082	0.0000098
**IVIG+G: baseline_weight_z_missingTRUE**	-1.6943983	0.2305578	-7.3491267	0.0000000
**IVIG+G: baseline_sig_comorbidityYes**	-1.9618243	0.2887382	-6.7944743	0.0000000
**IVIG+G: baseline_fever_days_at_treating_admission**	0.3873925	0.1927034	2.0103039	0.0443990
**IVIG+G: baseline_fever_days_missingTRUE**	-1.2798756	0.1912329	-6.6927592	0.0000000
**IVIG+G: genderMale**	-0.2115380	0.2885544	-0.7330958	0.4635000
**IVIG+G: baseline_number_clin_feat**	-1.5966681	0.2315833	-6.8945738	0.0000000
**IVIG+G: baseline_admission_day_at_treatment**	0.0330626	0.3487229	0.0948106	0.9244653
**IVIG+G: baseline_covid_positivePCR**	-1.6921022	0.2238540	-7.5589531	0.0000000
**IVIG+G: baseline_covid_positiveSerology**	0.7081940	0.2157062	3.2831412	0.0010266
**IVIG+G: baseline_peak_levin_LOCNo.support.CRP...50**	-1.1079819	0.2830362	-3.9146297	0.0000905
**IVIG+G: baseline_peak_levin_LOCNo.support.CRP.unknown**	0.5444296	0.3888916	1.3999522	0.1615276
**IVIG+G: baseline_peak_levin_LOCNo.support.CRP....50**	1.2637763	0.3971384	3.1822065	0.0014616
**IVIG+G: baseline_peak_levin_LOCOxygen**	1.3649816	0.3373189	4.0465608	0.0000520
**IVIG+G: baseline_peak_levin_LOCInotropes**	-0.4881318	0.3711278	-1.3152661	0.1884205
**IVIG+G: baseline_peak_levin_LOCVentilation**	2.4988654	0.2717252	9.1962961	0.0000000
**IVIG+G: baseline_peak_CRP.75th**	-1.8028860	0.2244911	-8.0309892	0.0000000
**IVIG+G: baseline_peak_CRP25.50th**	-1.1610928	0.2188572	-5.3052524	0.0000001
**IVIG+G: baseline_peak_CRP50.75th**	-1.0317221	0.2264094	-4.5568873	0.0000052
**IVIG+G: baseline_peak_CRPMissing**	-0.8985850	0.3451813	-2.6032262	0.0092351
**IVIG+G: baseline_peak_ddimer.75th**	1.2642562	0.3070198	4.1178328	0.0000382
**IVIG+G: baseline_peak_ddimer25.50th**	-1.1656636	0.3119981	-3.7361246	0.0001869
**IVIG+G: baseline_peak_ddimer50.75th**	-0.5809624	0.3279889	-1.7712864	0.0765131
**IVIG+G: baseline_peak_ddimerMissing**	0.6587579	0.3431995	1.9194607	0.0549261

Table S9 | Coefficients for covariate-balancing propensity score multinomial model
Primary outcome: improvement by day 2

J - statistic: 1.021868 Log-Likelihood: -426.1468	Estimate	Std. Error	z value	Pr(>|z|)
**IVIG: (Intercept)**	6.2087905	4.4554055	1.3935411	0.1634562
**IVIG: baseline_resource_groupHigh.income.economies**	1.0256911	0.1570956	6.5290896	0.0000000
**IVIG: baseline_resource_groupLower.middle.income.economies**	-0.4974489	0.1566625	-3.1752909	0.0014969
**IVIG: baseline_age**	0.3224193	0.1487008	2.1682421	0.0301403
**IVIG: baseline_weight_z_over_2**	-0.8039662	0.1807795	-4.4472192	0.0000087
**IVIG: baseline_weight_z_missingTRUE**	-2.6181433	0.1585445	-16.5136178	0.0000000
**IVIG: baseline_sig_comorbidityYes**	-1.1843598	0.2182033	-5.4277804	0.0000001
**IVIG: baseline_fever_days_at_treating_admission**	0.4004949	0.1828159	2.1907008	0.0284735
**IVIG: baseline_fever_days_missingTRUE**	-0.6343413	0.2209236	-2.8713156	0.0040877
**IVIG: genderMale**	0.0645182	0.2012557	0.3205783	0.7485300
**IVIG: baseline_number_clin_feat**	0.0536852	0.1795748	0.2989570	0.7649729
**IVIG: baseline_admission_day_at_treatment**	-0.0352195	0.1453885	-0.2422442	0.8085910
**IVIG: baseline_covid_positivePCR**	-0.9682758	0.1819756	-5.3209104	0.0000001
**IVIG: baseline_covid_positiveSerology**	0.5020153	0.1610568	3.1170075	0.0018270
**IVIG: baseline_peak_levin_LOCNo.support.CRP...50**	-2.7504627	0.1940379	-14.1748702	0.0000000
**IVIG: baseline_peak_levin_LOCNo.support.CRP.unknown**	0.0358762	0.2705509	0.1326041	0.8945065
**IVIG: baseline_peak_levin_LOCNo.support.CRP....50**	0.1880336	0.2866932	0.6558704	0.5119075
**IVIG: baseline_peak_levin_LOCOxygen**	-0.5790028	0.2091684	-2.7681186	0.0056381
**IVIG: baseline_peak_levin_LOCInotropes**	1.4360265	0.2113477	6.7946159	0.0000000
**IVIG: baseline_peak_levin_LOCVentilation**	-0.6814367	0.1935498	-3.5207311	0.0004304
**IVIG: baseline_peak_CRP.75th**	0.1141795	0.1844924	0.6188846	0.5359924
**IVIG: baseline_peak_CRP25.50th**	-0.2273779	0.1796400	-1.2657424	0.2056053
**IVIG: baseline_peak_CRP50.75th**	0.1512790	0.1729420	0.8747381	0.3817164
**IVIG: baseline_peak_CRPMissing**	1.7382398	0.1403379	12.3861026	0.0000000
**IVIG: baseline_peak_ddimer.75th**	0.4937935	0.2378905	2.0757179	0.0379201
**IVIG: baseline_peak_ddimer25.50th**	0.4379791	0.2262901	1.9354759	0.0529319
**IVIG: baseline_peak_ddimer50.75th**	-0.3195057	0.2244334	-1.4236099	0.1545594
**IVIG: baseline_peak_ddimerMissing**	0.8966589	0.2335803	3.8387600	0.0001237
**IVIG+G: (Intercept)**	2.9272363	6.8766626	0.4256769	0.6703433
**IVIG+G: baseline_resource_groupHigh.income.economies**	2.5751756	0.2624052	9.8137384	0.0000000
**IVIG+G: baseline_resource_groupLower.middle.income.economies**	-1.3400022	0.1798357	-7.4512598	0.0000000
**IVIG+G: baseline_age**	1.0941010	0.2668695	4.0997603	0.0000414
**IVIG+G: baseline_weight_z_over_2**	0.2004048	0.2504993	0.8000214	0.4236984
**IVIG+G: baseline_weight_z_missingTRUE**	-4.0567488	0.2732349	-14.8471118	0.0000000
**IVIG+G: baseline_sig_comorbidityYes**	-2.5214488	0.3387671	-7.4430149	0.0000000
**IVIG+G: baseline_fever_days_at_treating_admission**	0.2540829	0.2674601	0.9499844	0.3421202
**IVIG+G: baseline_fever_days_missingTRUE**	-1.1136317	0.3343701	-3.3305359	0.0008668
**IVIG+G: genderMale**	-0.1555245	0.2985997	-0.5208460	0.6024741
**IVIG+G: baseline_number_clin_feat**	-1.1577452	0.2449999	-4.7254926	0.0000023
**IVIG+G: baseline_admission_day_at_treatment**	0.1754861	0.2153247	0.8149835	0.4150817
**IVIG+G: baseline_covid_positivePCR**	-1.3040940	0.2884215	-4.5214860	0.0000061
**IVIG+G: baseline_covid_positiveSerology**	0.4947898	0.3037595	1.6288870	0.1033369
**IVIG+G: baseline_peak_levin_LOCNo.support.CRP...50**	-1.0915887	0.3129929	-3.4875824	0.0004874
**IVIG+G: baseline_peak_levin_LOCNo.support.CRP.unknown**	0.9080603	0.3873030	2.3445736	0.0190489
**IVIG+G: baseline_peak_levin_LOCNo.support.CRP....50**	0.8871051	0.4220534	2.1018788	0.0355639
**IVIG+G: baseline_peak_levin_LOCOxygen**	0.6429781	0.3119923	2.0608778	0.0393147
**IVIG+G: baseline_peak_levin_LOCInotropes**	0.5768457	0.3485192	1.6551333	0.0978975
**IVIG+G: baseline_peak_levin_LOCVentilation**	3.1125208	0.3303072	9.4231090	0.0000000
**IVIG+G: baseline_peak_CRP.75th**	-0.6711493	0.2585840	-2.5954791	0.0094459
**IVIG+G: baseline_peak_CRP25.50th**	-0.3699440	0.2397466	-1.5430625	0.1228156
**IVIG+G: baseline_peak_CRP50.75th**	-0.4361976	0.2404264	-1.8142668	0.0696367
**IVIG+G: baseline_peak_CRPMissing**	-0.0933997	0.1976525	-0.4725451	0.6365378
**IVIG+G: baseline_peak_ddimer.75th**	2.0472264	0.3386930	6.0444901	0.0000000
**IVIG+G: baseline_peak_ddimer25.50th**	-0.6182960	0.3438155	-1.7983368	0.0721237
**IVIG+G: baseline_peak_ddimer50.75th**	-0.4813378	0.3386380	-1.4213932	0.1552025
**IVIG+G: baseline_peak_ddimerMissing**	0.7455251	0.3476955	2.1441894	0.0320177

Table S10 | Coefficients for covariate-balancing propensity score multinomial model
WHO MIS-C: inotropes/ventilation day 2+ or death

J - statistic: 0.8325656 Log-Likelihood: -383.392	Estimate	Std. Error	z value	Pr(>|z|)
**IVIG: (Intercept)**	6.7570607	4.8937413	1.3807556	0.1673541
**IVIG: baseline_resource_groupHigh.income.economies**	0.7579875	0.2071986	3.6582653	0.0002539
**IVIG: baseline_resource_groupLower.middle.income.economies**	-2.5026254	0.1615026	-15.4958872	0.0000000
**IVIG: baseline_age**	0.1764513	0.1889932	0.9336380	0.3504906
**IVIG: baseline_weight_z_over_2**	-0.5165437	0.2374051	-2.1757899	0.0295710
**IVIG: baseline_weight_z_missingTRUE**	-1.7344596	0.2256947	-7.6849827	0.0000000
**IVIG: baseline_sig_comorbidityYes**	-2.3055166	0.1945926	-11.8479137	0.0000000
**IVIG: baseline_fever_days_at_treating_admission**	0.4675929	0.2083437	2.2443343	0.0248109
**IVIG: baseline_fever_days_missingTRUE**	-0.4361620	0.2142856	-2.0354232	0.0418083
**IVIG: genderMale**	0.0208245	0.1442207	0.1443932	0.8851900
**IVIG: baseline_number_clin_feat**	0.2462560	0.1886408	1.3054232	0.1917488
**IVIG: baseline_admission_day_at_treatment**	-0.2158588	0.1580558	-1.3657121	0.1720293
**IVIG: baseline_covid_positivePCR**	-0.1906752	0.2623994	-0.7266600	0.4674343
**IVIG: baseline_covid_positiveSerology**	-0.5367068	0.2582702	-2.0780826	0.0377018
**IVIG: baseline_peak_levin_LOCNo.support.CRP...50**	-2.4132790	0.1990227	-12.1256472	0.0000000
**IVIG: baseline_peak_levin_LOCNo.support.CRP.unknown**	0.6409686	0.2686110	2.3862336	0.0170219
**IVIG: baseline_peak_levin_LOCNo.support.CRP....50**	0.6186316	0.2859891	2.1631298	0.0305312
**IVIG: baseline_peak_levin_LOCOxygen**	0.1905222	0.2649242	0.7191572	0.4720440
**IVIG: baseline_peak_levin_LOCInotropes**	1.0738660	0.2425416	4.4275534	0.0000095
**IVIG: baseline_peak_levin_LOCVentilation**	-0.6482202	0.1424515	-4.5504622	0.0000054
**IVIG: baseline_peak_CRP.75th**	0.0733777	0.2688172	0.2729650	0.7848801
**IVIG: baseline_peak_CRP25.50th**	-0.4292321	0.2634300	-1.6293968	0.1032291
**IVIG: baseline_peak_CRP50.75th**	0.7225624	0.2304757	3.1350913	0.0017180
**IVIG: baseline_peak_CRPMissing**	1.1915781	0.1837588	6.4844696	0.0000000
**IVIG: baseline_peak_ddimer.75th**	1.0623448	0.2327594	4.5641334	0.0000050
**IVIG: baseline_peak_ddimer25.50th**	0.2362024	0.1729005	1.3661180	0.1719019
**IVIG: baseline_peak_ddimer50.75th**	-0.5869631	0.1738248	-3.3767523	0.0007335
**IVIG: baseline_peak_ddimerMissing**	0.8651297	0.2189859	3.9506178	0.0000779
**IVIG+G: (Intercept)**	17.1556074	9.1096727	1.8832298	0.0596692
**IVIG+G: baseline_resource_groupHigh.income.economies**	2.8102455	0.2895786	9.7046032	0.0000000
**IVIG+G: baseline_resource_groupLower.middle.income.economies**	-2.8157735	0.2294032	-12.2743423	0.0000000
**IVIG+G: baseline_age**	0.9922981	0.4939373	2.0089555	0.0445419
**IVIG+G: baseline_weight_z_over_2**	0.6166618	0.5156704	1.1958449	0.2317571
**IVIG+G: baseline_weight_z_missingTRUE**	-1.9018983	0.4266601	-4.4576427	0.0000083
**IVIG+G: baseline_sig_comorbidityYes**	-3.4968213	0.3539877	-9.8783697	0.0000000
**IVIG+G: baseline_fever_days_at_treating_admission**	0.8586581	0.3757605	2.2851208	0.0223057
**IVIG+G: baseline_fever_days_missingTRUE**	-2.5546394	0.4438105	-5.7561490	0.0000000
**IVIG+G: genderMale**	0.1850045	0.4762645	0.3884491	0.6976837
**IVIG+G: baseline_number_clin_feat**	-1.4955455	0.3221473	-4.6424267	0.0000034
**IVIG+G: baseline_admission_day_at_treatment**	-0.2823823	0.3131069	-0.9018717	0.3671250
**IVIG+G: baseline_covid_positivePCR**	-0.9129338	0.4467291	-2.0435960	0.0409935
**IVIG+G: baseline_covid_positiveSerology**	-0.1676171	0.4370840	-0.3834895	0.7013569
**IVIG+G: baseline_peak_levin_LOCNo.support.CRP...50**	-4.8601043	0.3409385	-14.2550748	0.0000000
**IVIG+G: baseline_peak_levin_LOCNo.support.CRP.unknown**	-1.9456557	0.4224032	-4.6061578	0.0000041
**IVIG+G: baseline_peak_levin_LOCNo.support.CRP....50**	-0.8345144	0.4893711	-1.7052793	0.0881423
**IVIG+G: baseline_peak_levin_LOCOxygen**	-0.5161434	0.4515802	-1.1429716	0.2530504
**IVIG+G: baseline_peak_levin_LOCInotropes**	1.8540887	0.4782456	3.8768549	0.0001058
**IVIG+G: baseline_peak_levin_LOCVentilation**	0.5345090	0.2475489	2.1592059	0.0308342
**IVIG+G: baseline_peak_CRP.75th**	-2.6601369	0.4384927	-6.0665472	0.0000000
**IVIG+G: baseline_peak_CRP25.50th**	-1.9665872	0.4879568	-4.0302486	0.0000557
**IVIG+G: baseline_peak_CRP50.75th**	-1.4004961	0.4000502	-3.5008014	0.0004639
**IVIG+G: baseline_peak_CRPMissing**	1.3404591	0.3276091	4.0916414	0.0000428
**IVIG+G: baseline_peak_ddimer.75th**	2.4073701	0.3184987	7.5584931	0.0000000
**IVIG+G: baseline_peak_ddimer25.50th**	-1.4617189	0.2547130	-5.7386892	0.0000000
**IVIG+G: baseline_peak_ddimer50.75th**	-0.6514437	0.2825733	-2.3053976	0.0211443
**IVIG+G: baseline_peak_ddimerMissing**	0.5009076	0.4020522	1.2458769	0.2128096

Table S11 | Coefficients for covariate-balancing propensity score multinomial model
WHO MIS-C: improvement by day 2

J - statistic: 1.624243 Log-Likelihood: -367.0137	Estimate	Std. Error	z value	Pr(>|z|)
**IVIG: (Intercept)**	6.9214310	4.7033819	1.4715860	0.1411327
**IVIG: baseline_resource_groupHigh.income.economies**	0.9219918	0.1620957	5.6879480	0.0000000
**IVIG: baseline_resource_groupLower.middle.income.economies**	-2.3185373	0.1775774	-13.0564876	0.0000000
**IVIG: baseline_age**	0.2081126	0.2733497	0.7613421	0.4464528
**IVIG: baseline_weight_z_over_2**	-0.4545378	0.1887692	-2.4079021	0.0160445
**IVIG: baseline_weight_z_missingTRUE**	-2.2467441	0.1842572	-12.1935239	0.0000000
**IVIG: baseline_sig_comorbidityYes**	-3.1235955	0.2283978	-13.6761206	0.0000000
**IVIG: baseline_fever_days_at_treating_admission**	0.6404073	0.2315007	2.7663303	0.0056691
**IVIG: baseline_fever_days_missingTRUE**	-1.6029855	0.2202776	-7.2771165	0.0000000
**IVIG: genderMale**	0.2842586	0.2370032	1.1993875	0.2303773
**IVIG: baseline_number_clin_feat**	0.1777595	0.2025471	0.8776205	0.3801497
**IVIG: baseline_admission_day_at_treatment**	-0.2476265	0.1643947	-1.5062920	0.1319922
**IVIG: baseline_covid_positivePCR**	0.2783239	0.2748842	1.0125134	0.3112927
**IVIG: baseline_covid_positiveSerology**	-0.7302890	0.2619896	-2.7874732	0.0053121
**IVIG: baseline_peak_levin_LOCNo.support.CRP...50**	-2.8290638	0.2206877	-12.8193076	0.0000000
**IVIG: baseline_peak_levin_LOCNo.support.CRP.unknown**	0.7511316	0.3046027	2.4659390	0.0136655
**IVIG: baseline_peak_levin_LOCNo.support.CRP....50**	0.7051259	0.2760760	2.5541003	0.0106463
**IVIG: baseline_peak_levin_LOCOxygen**	-0.1383207	0.2576387	-0.5368786	0.5913515
**IVIG: baseline_peak_levin_LOCInotropes**	1.1774950	0.2683684	4.3876074	0.0000115
**IVIG: baseline_peak_levin_LOCVentilation**	-0.1744537	0.1971529	-0.8848650	0.3762295
**IVIG: baseline_peak_CRP.75th**	0.2270121	0.2757454	0.8232671	0.4103561
**IVIG: baseline_peak_CRP25.50th**	-0.2251256	0.2940386	-0.7656328	0.4438948
**IVIG: baseline_peak_CRP50.75th**	0.5399641	0.2540901	2.1250890	0.0335792
**IVIG: baseline_peak_CRPMissing**	2.2462739	0.1763733	12.7359082	0.0000000
**IVIG: baseline_peak_ddimer.75th**	1.2772028	0.2276389	5.6106527	0.0000000
**IVIG: baseline_peak_ddimer25.50th**	0.2074110	0.1791286	1.1578885	0.2469096
**IVIG: baseline_peak_ddimer50.75th**	-0.4800391	0.2139387	-2.2438161	0.0248442
**IVIG: baseline_peak_ddimerMissing**	1.4249262	0.1836085	7.7606752	0.0000000
**IVIG+G: (Intercept)**	15.9536251	9.2853723	1.7181460	0.0857700
**IVIG+G: baseline_resource_groupHigh.income.economies**	3.2990419	0.5063786	6.5149707	0.0000000
**IVIG+G: baseline_resource_groupLower.middle.income.economies**	-2.6882377	0.4113593	-6.5350120	0.0000000
**IVIG+G: baseline_age**	1.2276587	0.5560856	2.2076795	0.0272666
**IVIG+G: baseline_weight_z_over_2**	-0.3086173	0.3340857	-0.9237667	0.3556078
**IVIG+G: baseline_weight_z_missingTRUE**	-4.2251966	0.3629301	-11.6419030	0.0000000
**IVIG+G: baseline_sig_comorbidityYes**	-2.3465135	0.4260041	-5.5081943	0.0000000
**IVIG+G: baseline_fever_days_at_treating_admission**	0.6329291	0.5005709	1.2644146	0.2060813
**IVIG+G: baseline_fever_days_missingTRUE**	-2.2287431	0.4747304	-4.6947556	0.0000027
**IVIG+G: genderMale**	0.2459523	0.4620678	0.5322862	0.5945278
**IVIG+G: baseline_number_clin_feat**	-0.6902951	0.3534257	-1.9531548	0.0508013
**IVIG+G: baseline_admission_day_at_treatment**	-0.6547799	0.5024081	-1.3032828	0.1924782
**IVIG+G: baseline_covid_positivePCR**	-0.6594455	0.5465511	-1.2065578	0.2276025
**IVIG+G: baseline_covid_positiveSerology**	-0.3566245	0.4971788	-0.7172963	0.4731913
**IVIG+G: baseline_peak_levin_LOCNo.support.CRP...50**	-4.9043613	0.3772517	-13.0002381	0.0000000
**IVIG+G: baseline_peak_levin_LOCNo.support.CRP.unknown**	-1.5537708	0.5140837	-3.0224081	0.0025077
**IVIG+G: baseline_peak_levin_LOCNo.support.CRP....50**	-0.1978904	0.5367914	-0.3686541	0.7123856
**IVIG+G: baseline_peak_levin_LOCOxygen**	-0.7743659	0.4894305	-1.5821775	0.1136091
**IVIG+G: baseline_peak_levin_LOCInotropes**	1.9643395	0.4259248	4.6119393	0.0000040
**IVIG+G: baseline_peak_levin_LOCVentilation**	-1.0700221	0.4026325	-2.6575650	0.0078707
**IVIG+G: baseline_peak_CRP.75th**	-2.5428766	0.4950475	-5.1366320	0.0000003
**IVIG+G: baseline_peak_CRP25.50th**	-1.8892584	0.4889947	-3.8635562	0.0001117
**IVIG+G: baseline_peak_CRP50.75th**	-1.4697895	0.4119465	-3.5679140	0.0003598
**IVIG+G: baseline_peak_CRPMissing**	3.8401372	0.4377577	8.7722891	0.0000000
**IVIG+G: baseline_peak_ddimer.75th**	2.9546530	0.3407864	8.6701012	0.0000000
**IVIG+G: baseline_peak_ddimer25.50th**	-0.6406178	0.3744696	-1.7107338	0.0871303
**IVIG+G: baseline_peak_ddimer50.75th**	-0.7934098	0.3906214	-2.0311479	0.0422400
**IVIG+G: baseline_peak_ddimerMissing**	0.5982886	0.3513954	1.7026076	0.0886415

Supplementary Figures

Figure S1 | World map displaying the location of countries registered to the Best Available Treatment Study.
BATS patients were enrolled from across five continents (Europe, Asia, Africa, North America, and South America). Each blue dot may correspond to more than 1 site.

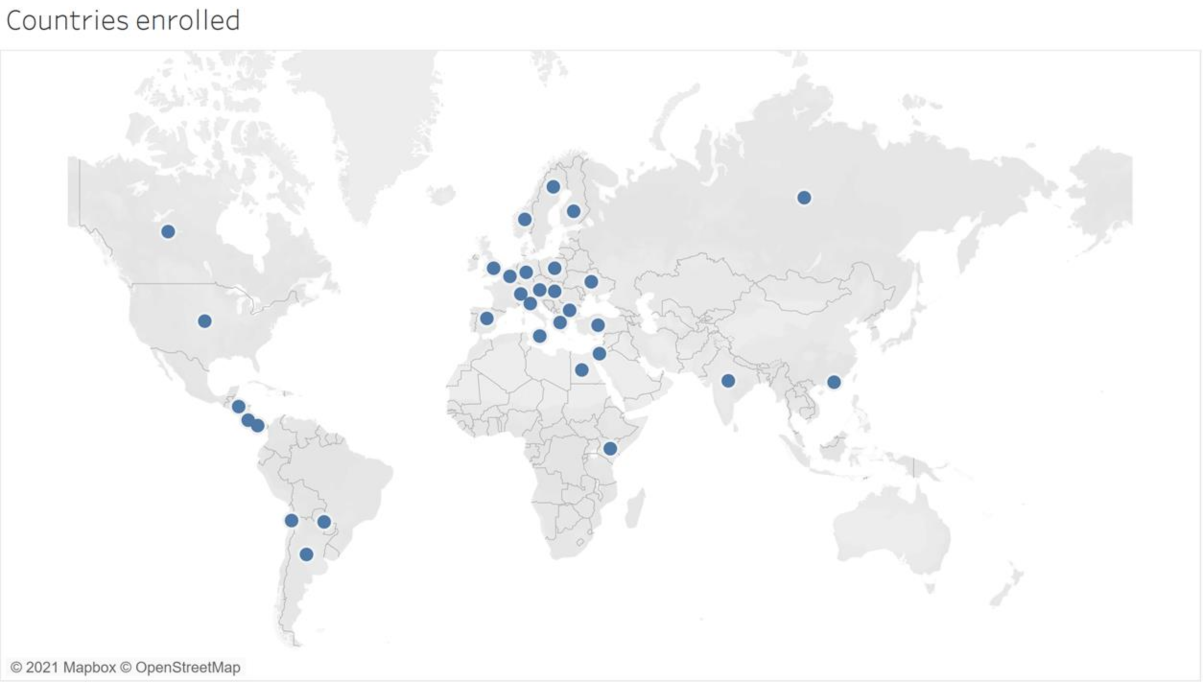

Figure S2a | Number of enrolment sites registered per country.
Data used in this figure is following exclusions. (countries in reverse alphabetical order)

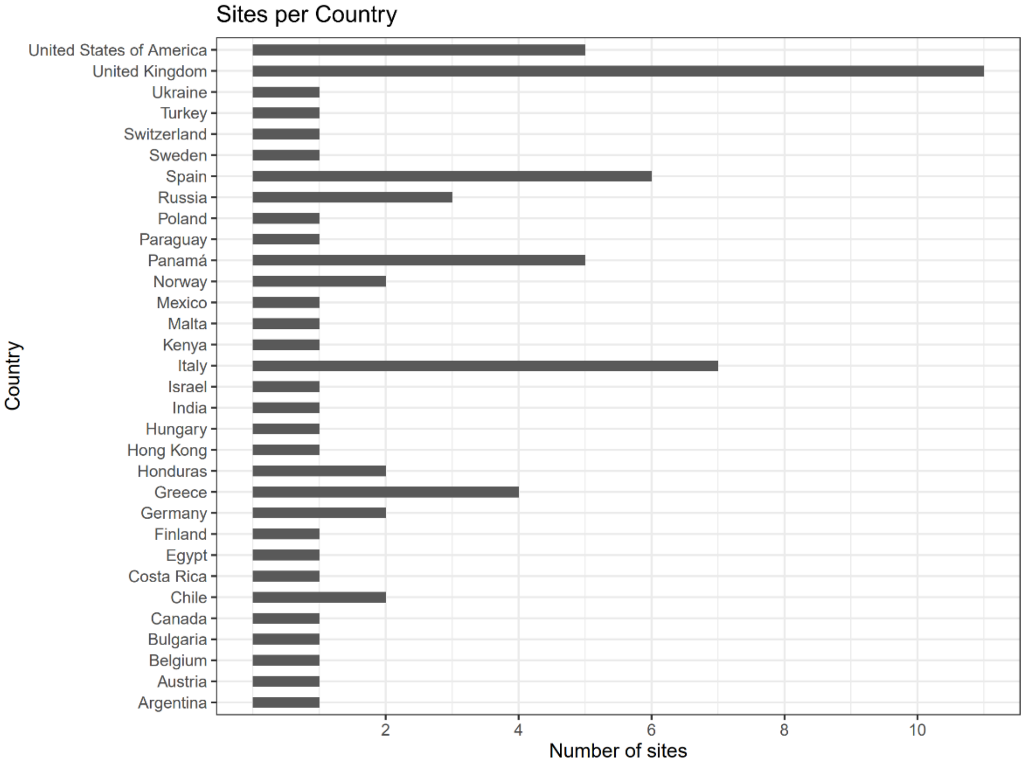

Figure S2b | Number of patients enrolled in BATS per country.
Data used in this figure is following exclusions. (countries in reverse alphabetical order)

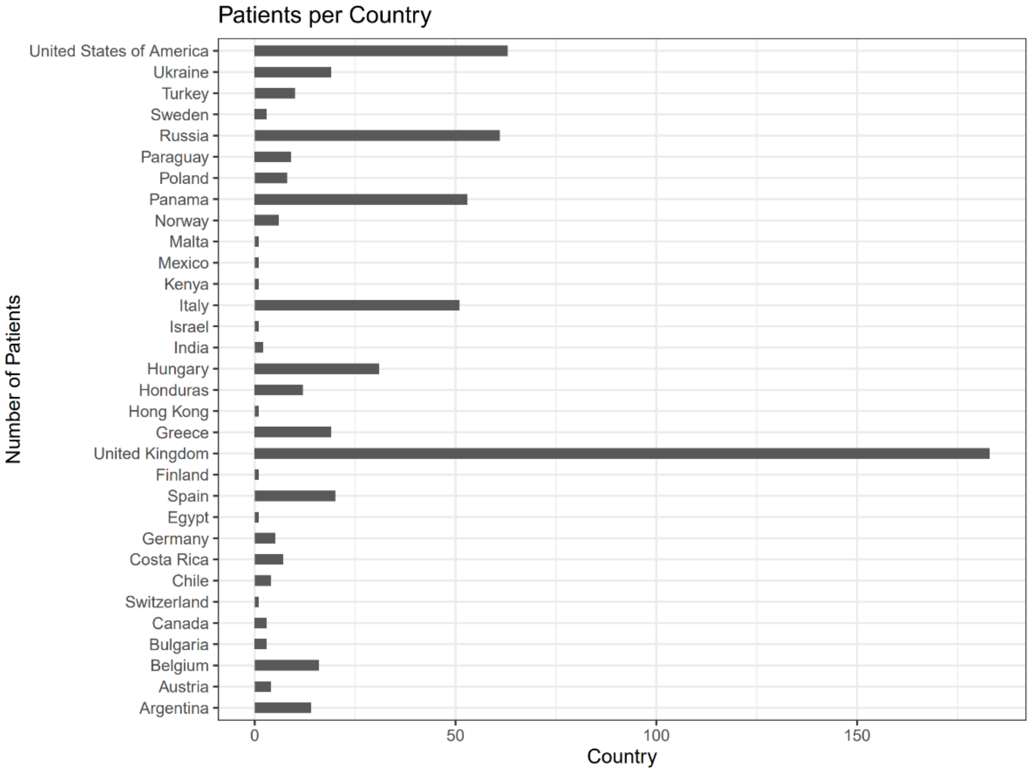

Figure S2c | Number of patients enrolled in BATS by sites in each country.
Data used in this figure is following exclusions. (countries in reverse alphabetical order)

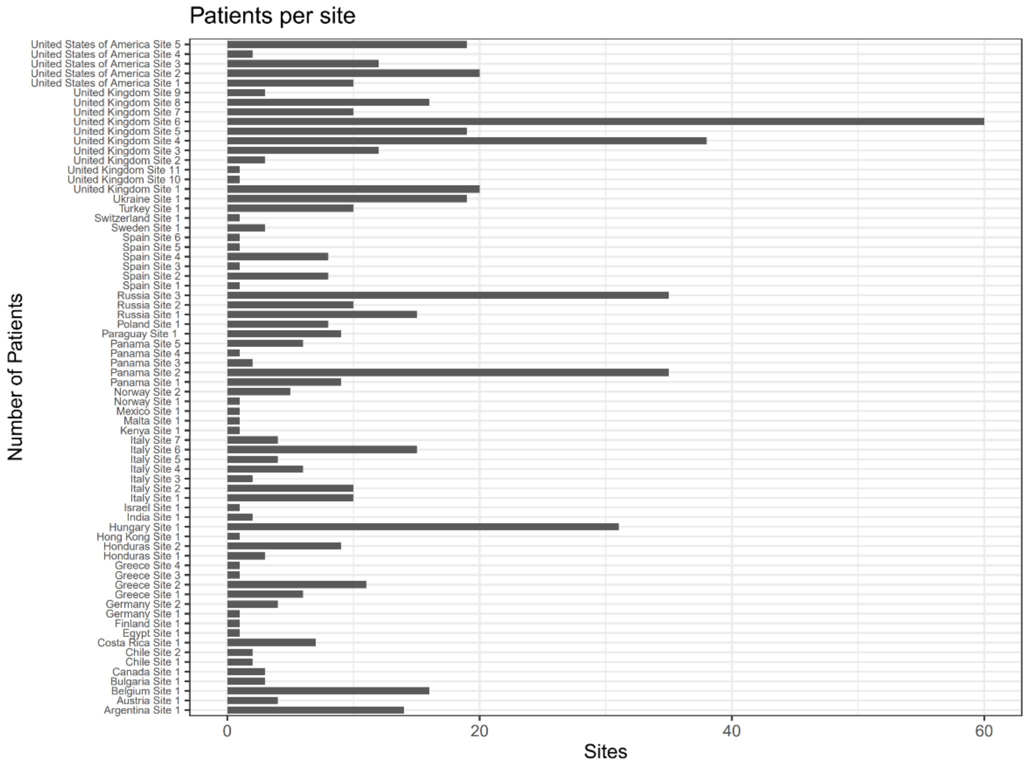

Figure S3 | BATS registration by month between May 2020 and February 2021.

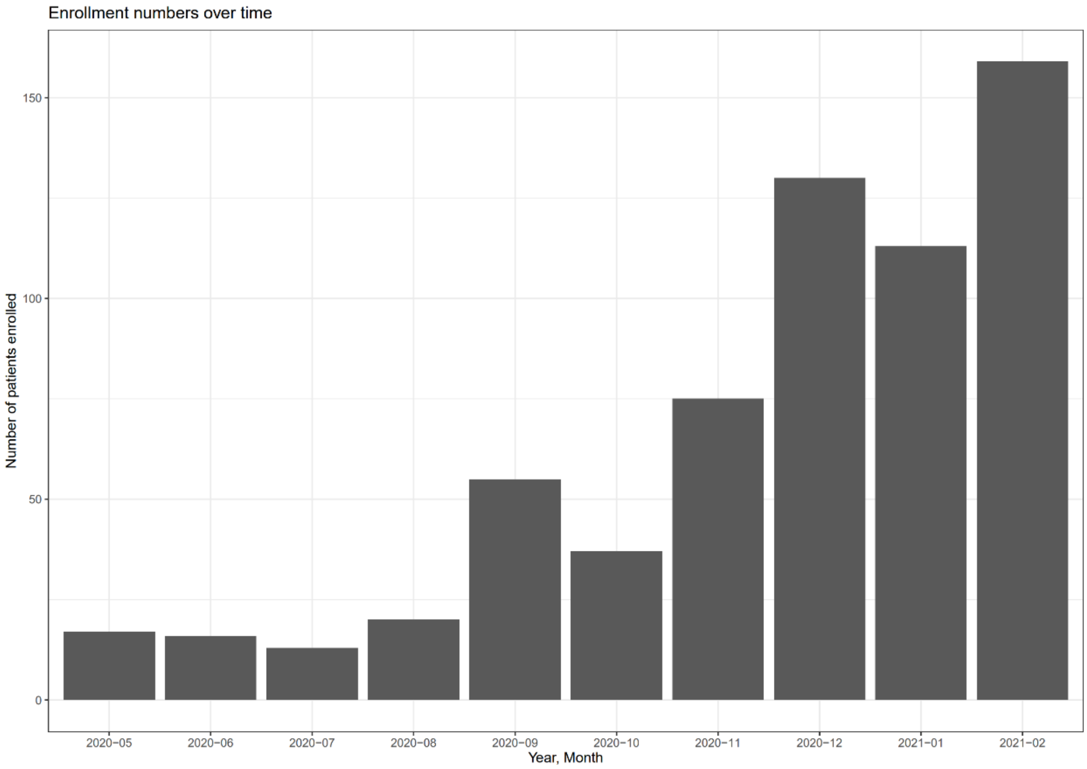

Figure S4 | Comparison of blood results across treatment groups at day 0.
Comparison of blood results by first immunomodulator treatment given at day 0. Statistical significance was calculated using the t-test comparing the blood results in each group versus all other groups. ns: P > 0.05; *: P ≤ 0.05; **: P ≤ 0.01; ***: P ≤ 0.001

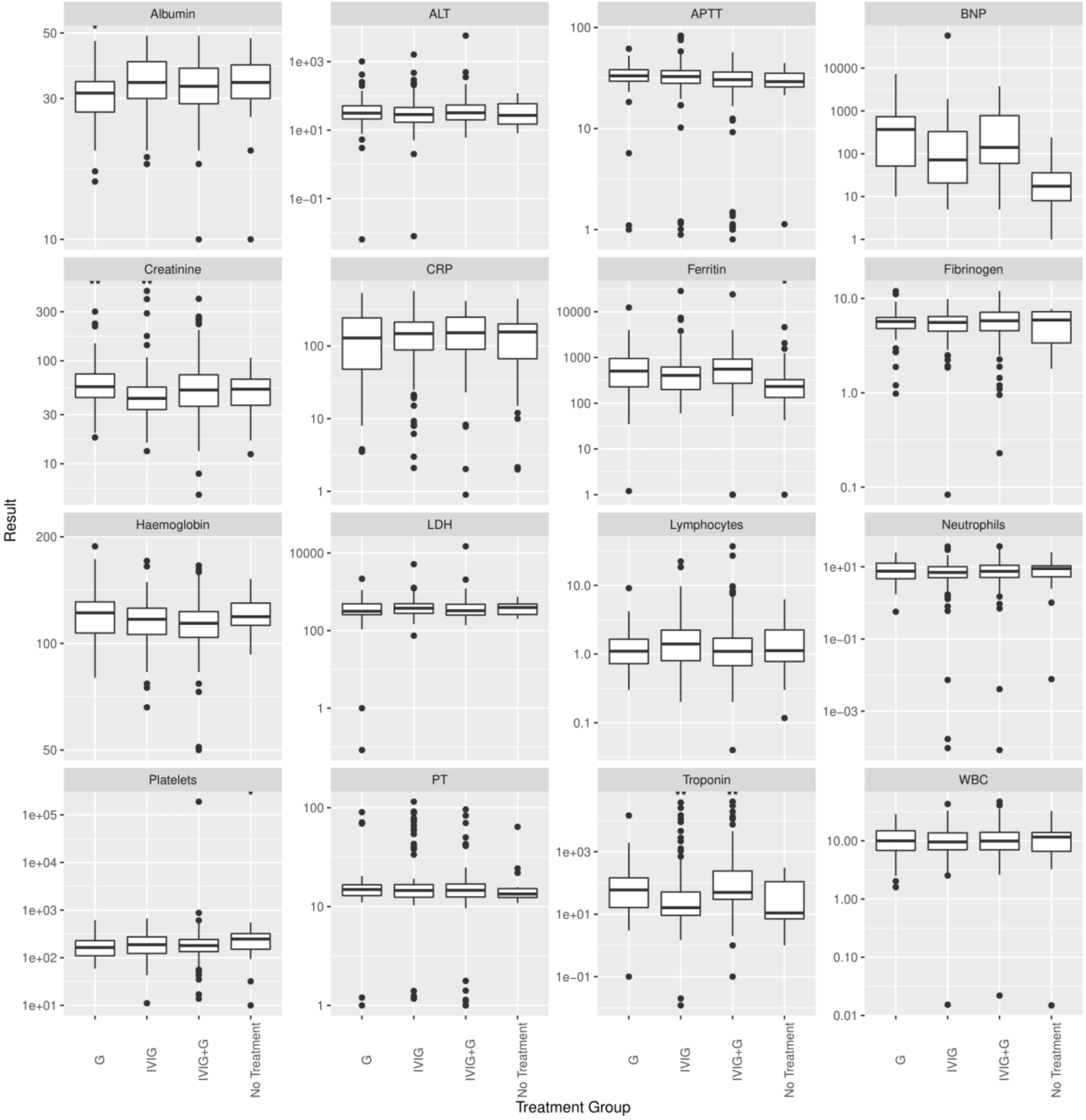

Figure S5 | Comparison of baseline CRP and troponin across treatment groups between day 0 and day 2

Comparison of baseline CRP and baseline troponin by treatment group at day 2. If a patient did not change primary treatment between day 0 and 2, they were classified by that primary treatment (IVIG, IVIG+Glucocorticoid, or glucocorticoid). Patients that changed treatment arm (of IVIG, IVIG+Glucocorticoid, or glucocorticoid) between day 0 and day 2, were classified as "switched Rx arm". Patients who started on primary treatments (IVIG, IVIG+Glucocorticoid, or glucocorticoid) but switched to a biological agent, or a biological agent was added, was classified as "switched to biological". Statistical significance was calculated using the t-test comparing the blood results in each group versus all other groups ns: P > 0.05; *: P ≤ 0.05; **: P ≤ 0.01; ***: P ≤ 0.001.

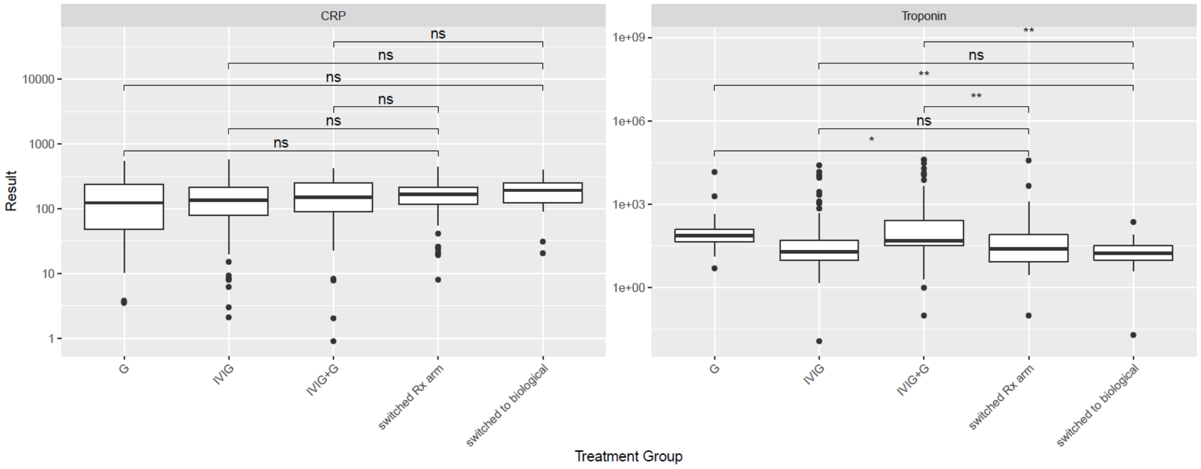

Figure S6A | Proportion of patients on inotropes or ventilated at baseline across treatment arms at day 0.

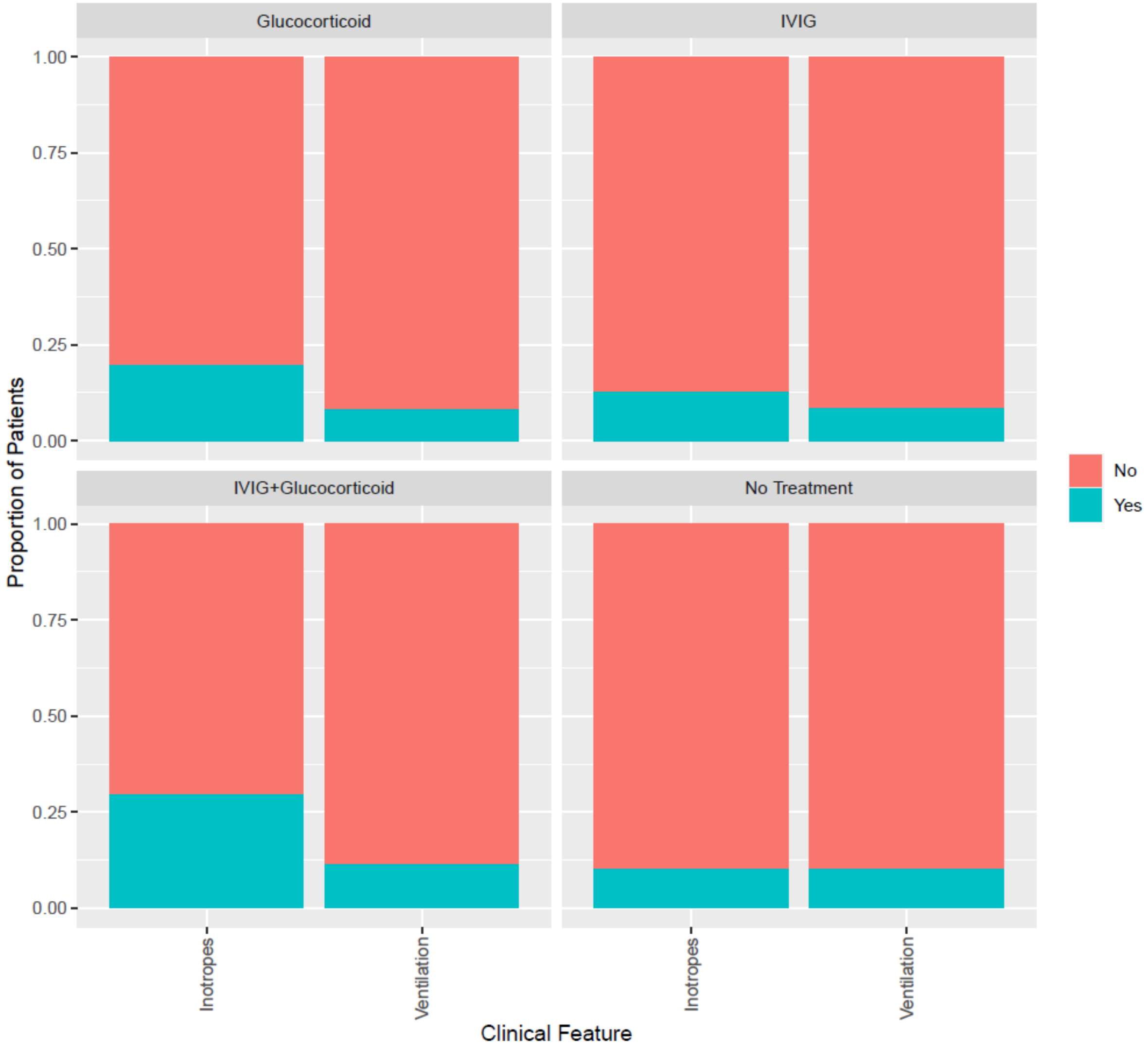

Figure S6B | Proportion of patients on inotropes or ventilated at baseline across treatment groups between day 0 and day 2.
Proportion of patients on inotropes or requiring inotropes at baseline grouped by treatment group at day 2. If a patient did not change primary treatment between day 0 and 2, they were classified by that primary treatment (IVIG, IVIG+Glucocorticoid, or glucocorticoid). Patients that changed treatment arm (of IVIG, IVIG+Glucocorticoid, or glucocorticoid) between day 0 and day 2, were classified as “switched Rx arm”. Patients who started on primary treatments (IVIG, IVIG+glucocorticoid, or glucocorticoid) but switched to a biological agent, or a biological agent was added, was classified as “switched to biological”.

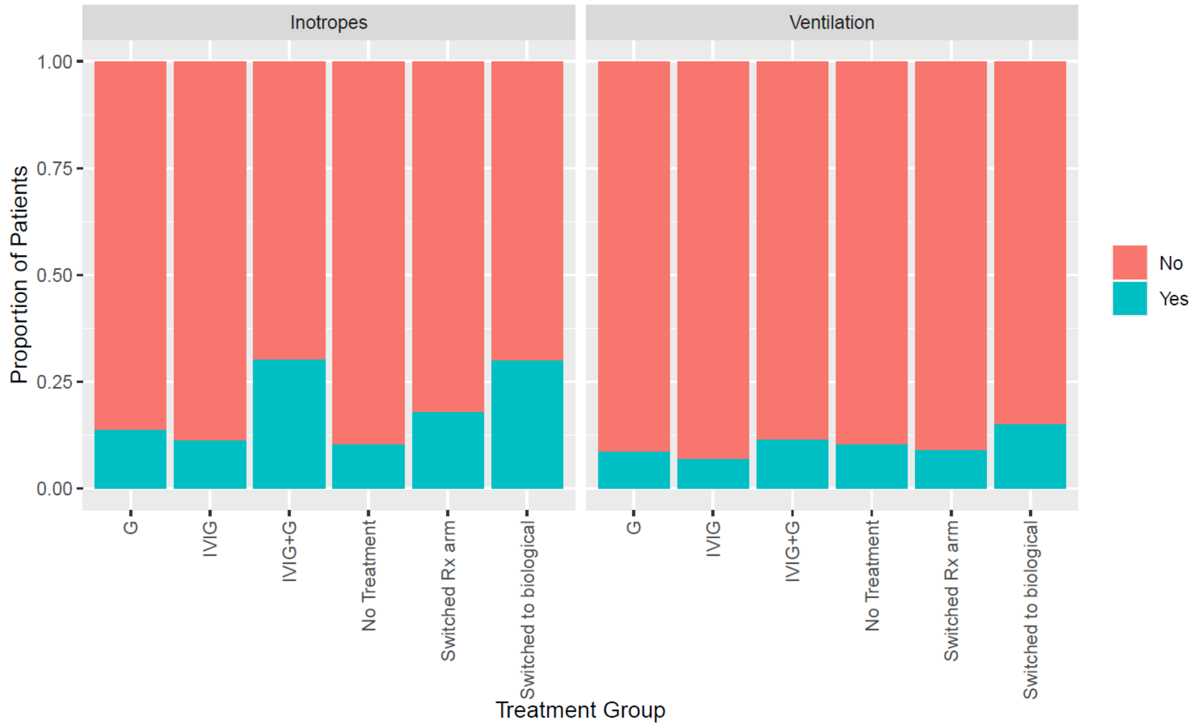

Figure S7 | Missing components in patients with all but one component of the WHO MIS-C criteria
Patients not meeting the full WHO criteria were subsetted by mandatory criteria to assess the most common reasons that they missed full classification. The COVID-19 criterion was defined as evidence of SARS-CoV-2 on RT-PCR, positive antibody result, or likely contact with COVID-19 patients.

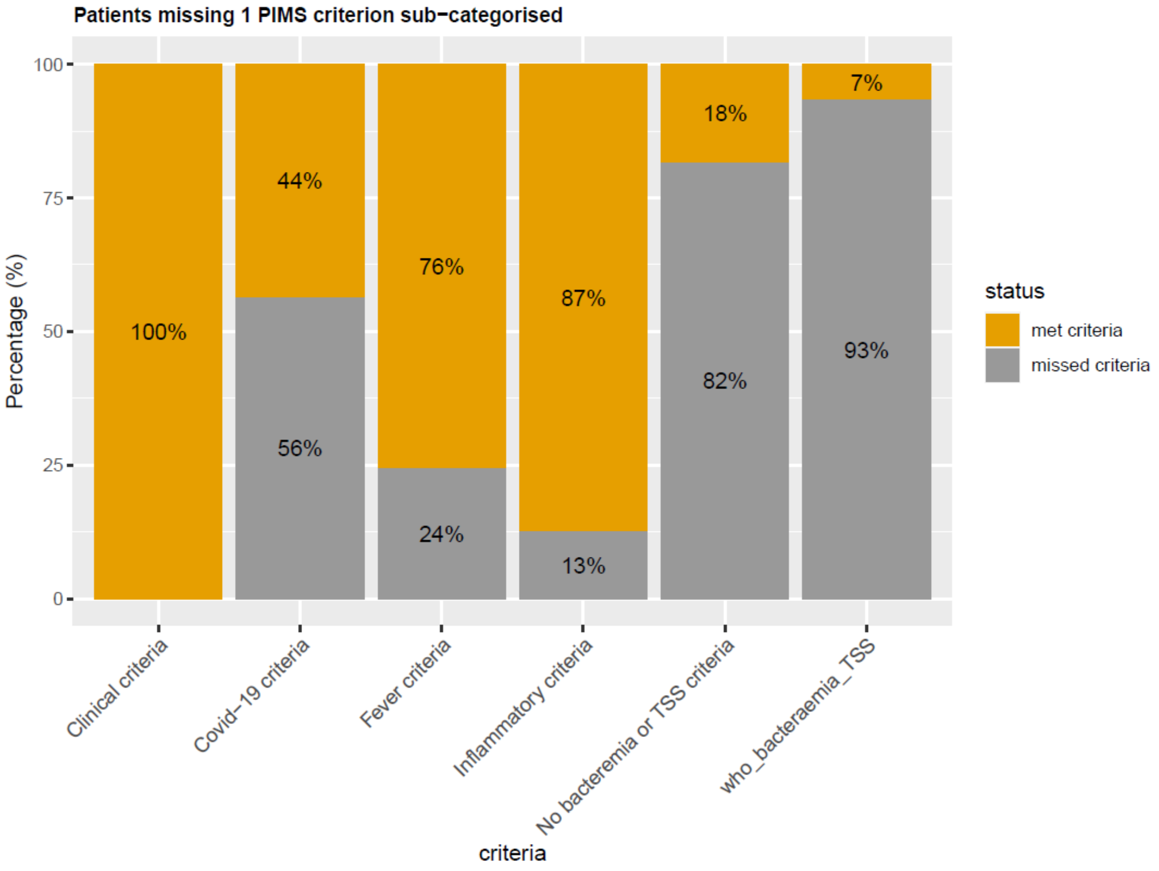

Figure S8 | Proportion of patients with clinical features of Kawasaki disease across treatment groups at day 0

Proportion of patients with clinical features of Kawasaki disease and those that met the criteria for classical and typical Kawasaki Disease across treatment groups at day 0.

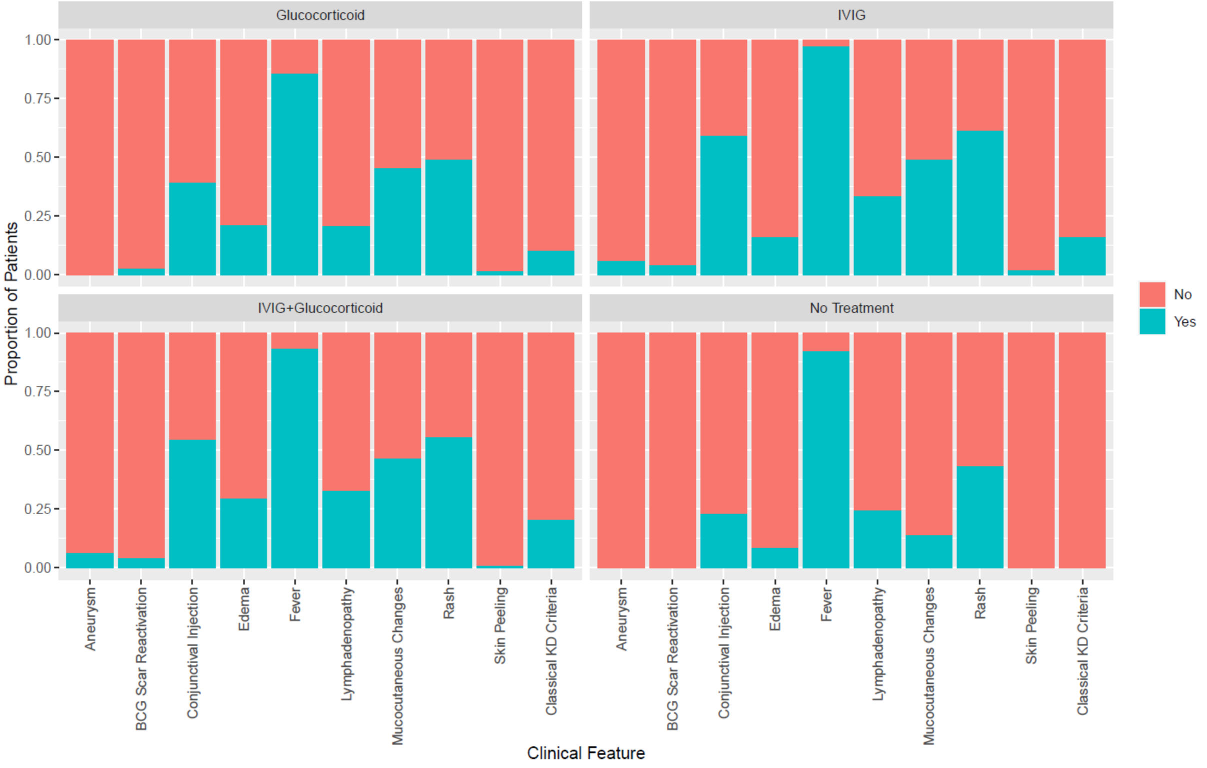

Figure S9 | Clinical improvement over time

(A) Kaplan-Meier chart showing time to one-point improvement in clinical severity on ordinal scale weighted by inverse probability of treatment. (B) Clinical severity shown by day relative to first treatment for patients by treatment group, with weighting by inverse probability of treatment.

A

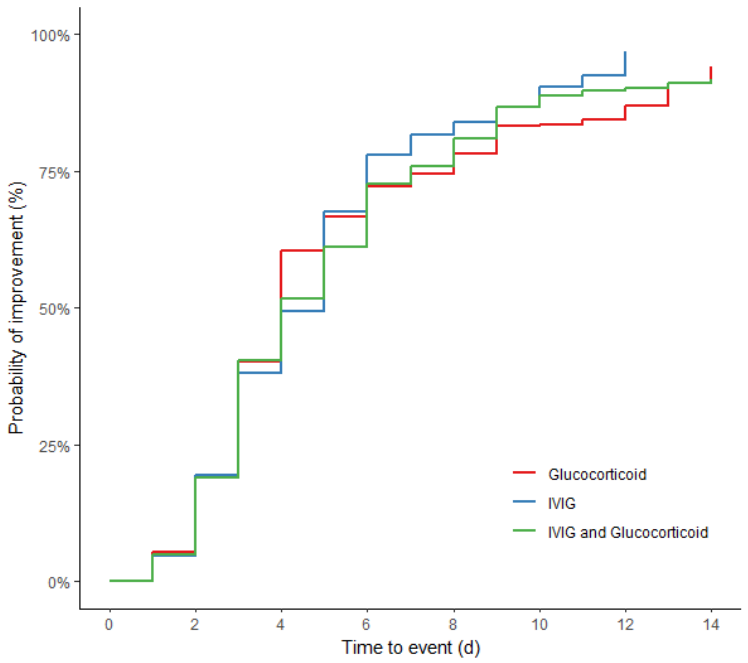

B

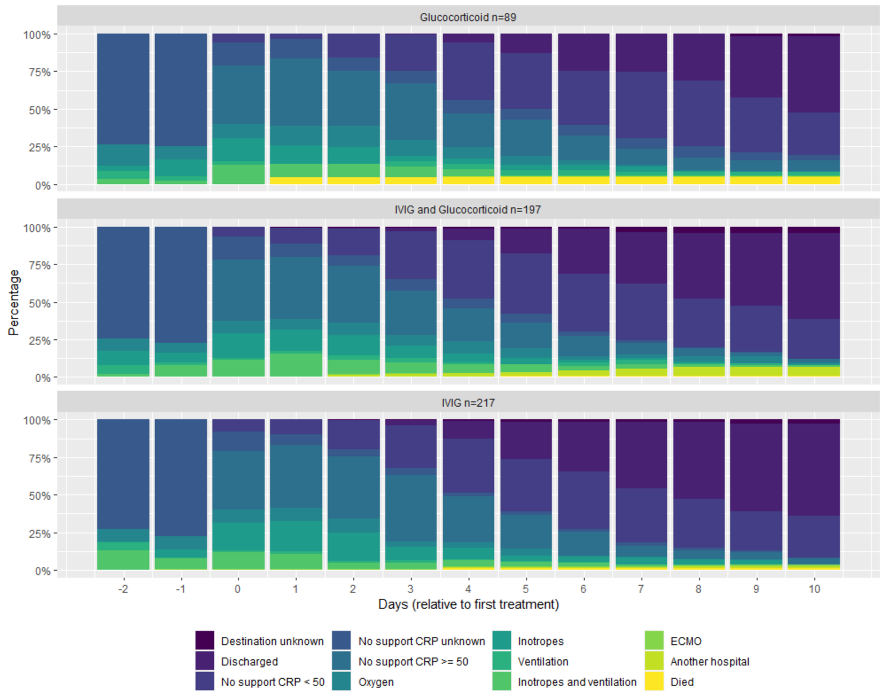

Figure S10 | Percentage of the CRP peak value by admission day for three primary treatments (IVIG, glucocortioids and IVIG and glucocorticoids combined).

Percentage of the CRP peak value by admission day for three primary treatments (IVIG, glucocorticoid and IVIG and glucocorticoid combined). CRP was plotted for each patient and at each time point (day) as a line, weighted by covariate-balancing propensity scores (CBPS) and fitted by a generalized additive model (GAM) for each treatment group. Panel A shows the fitted curves for CRP of children receiving IVIG, glucocorticoid and IVIG+glucocorticoid on the day of admission, younger versus older than 6 years old. Panel B shows the fitted curves for CRP of children who were given IVIG, glucocorticoid or IVIG and glucocorticoid combined, on the day of admission. The fitted curves represent children who meet the KD AHA criteria and are younger than 6 years old, and children who do not meet the KD AHA criteria or are older than 6 years old. Panel C shows the fitted curves for CRP of children who were given glucocorticoids or IVIG alone and whose treatment remained the same between the day of admission and day 3. The fitted curves represent children who meet the KD AHA criteria and are younger than 6 years old, and children who do not meet the KD AHA criteria or are older than 6 years old.

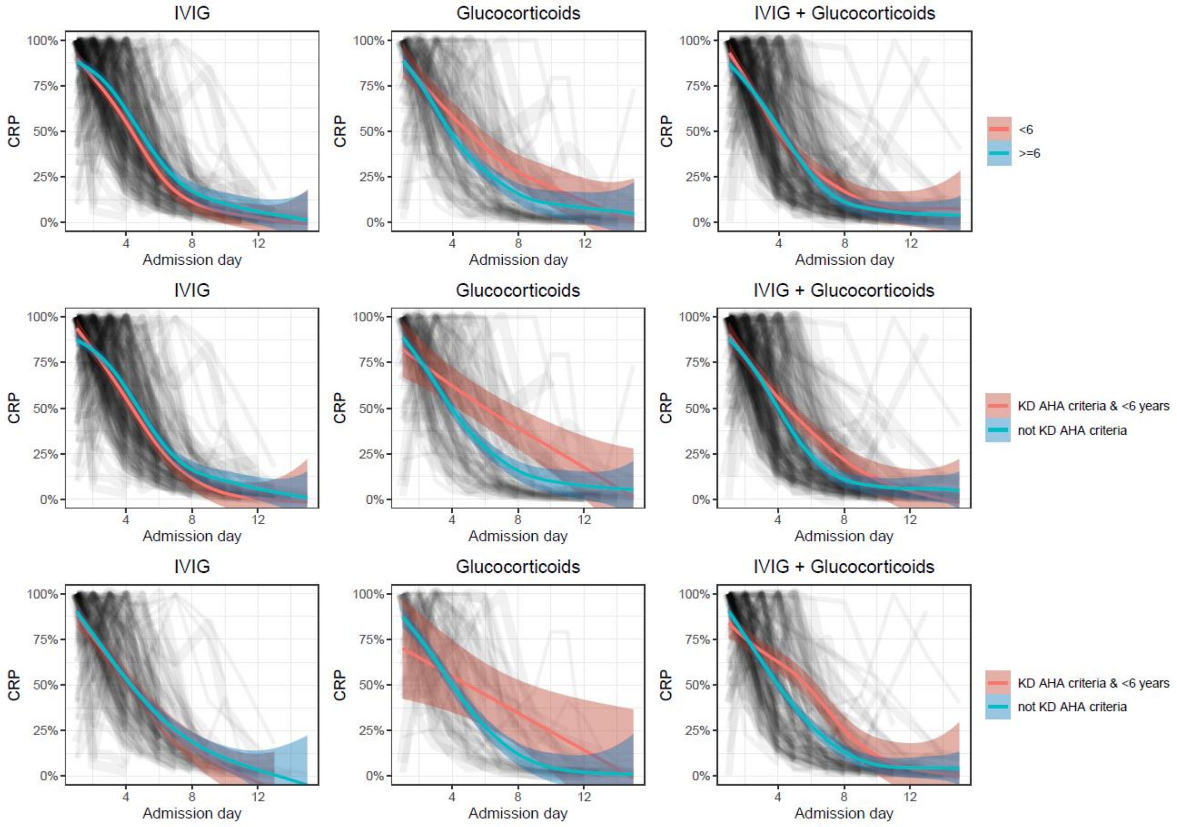

Figure S11 | Inverse probability weight distributions and covariate balance plots

Column A contains unstandardized inverse probability weight distributions for the three treatment groups derived from the covariate-balancing propensity score models. Column B contains covariate balance plots. Red coloured points show unadjusted absolute standardized mean differences; blue coloured line reflects absolute standardized mean differences following covariate-balancing propensity score weighting.

**Primary outcome: inotropes/ventilation day 2+ or /death A**	**B**
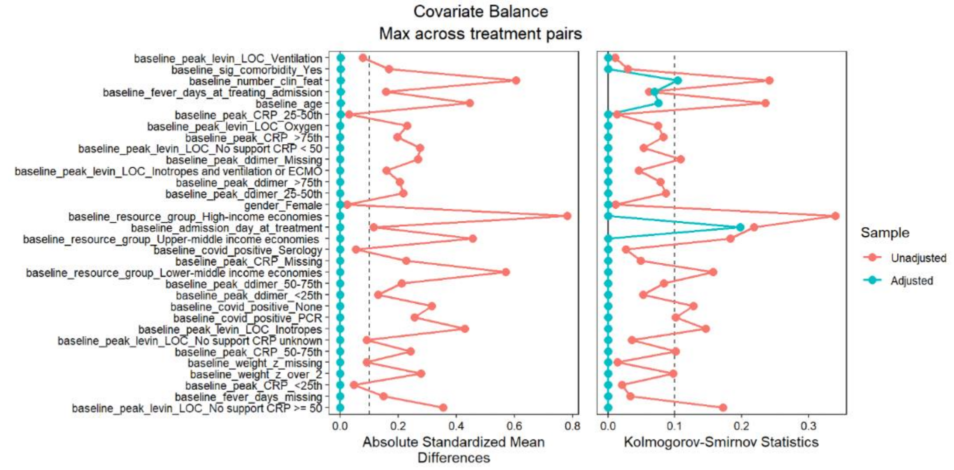	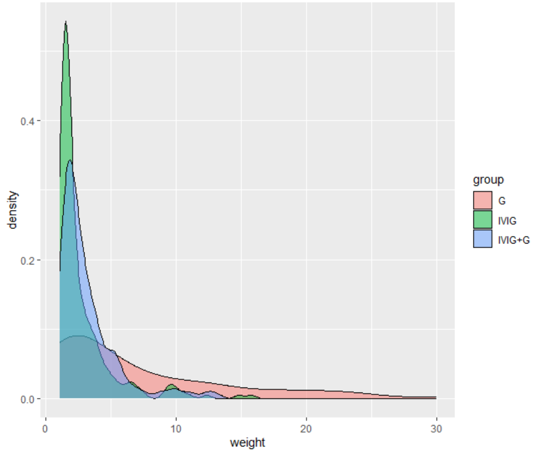
**Primary outcome: Improvement**	
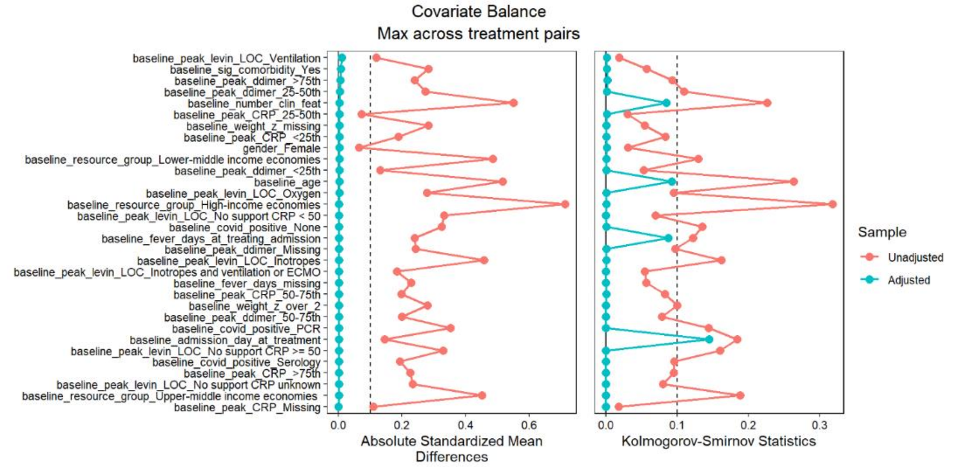	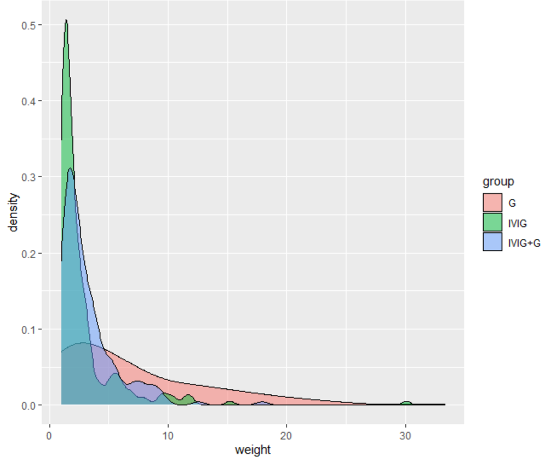

WHO MIS-C subgroup: inotropes/ventilation day 2+ or /death

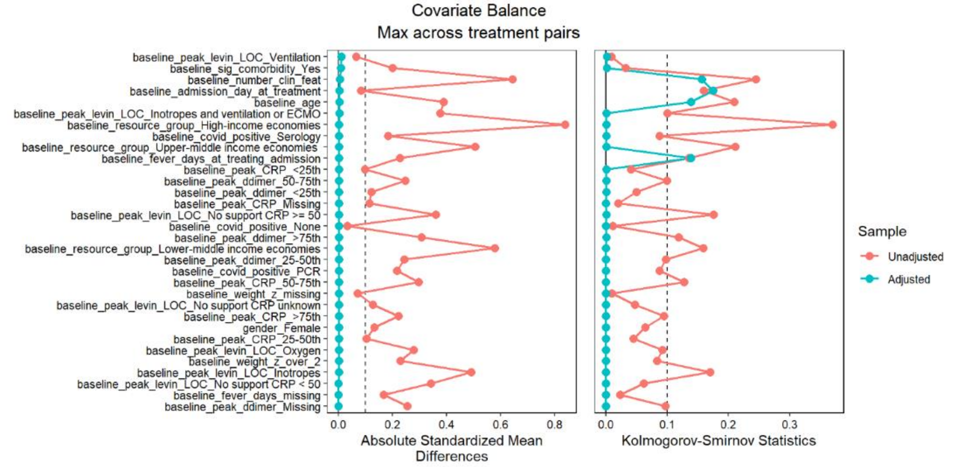

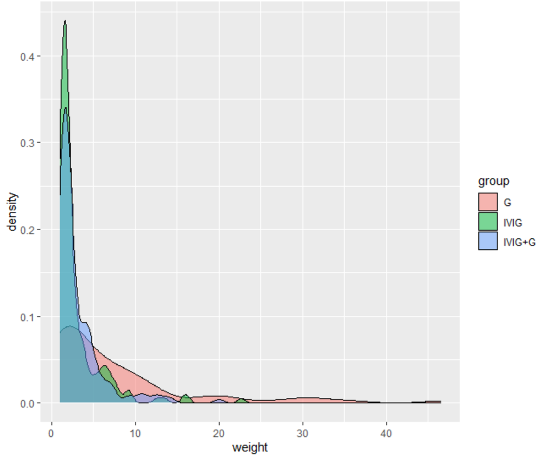

WHO MIS-C subgroup: improvement

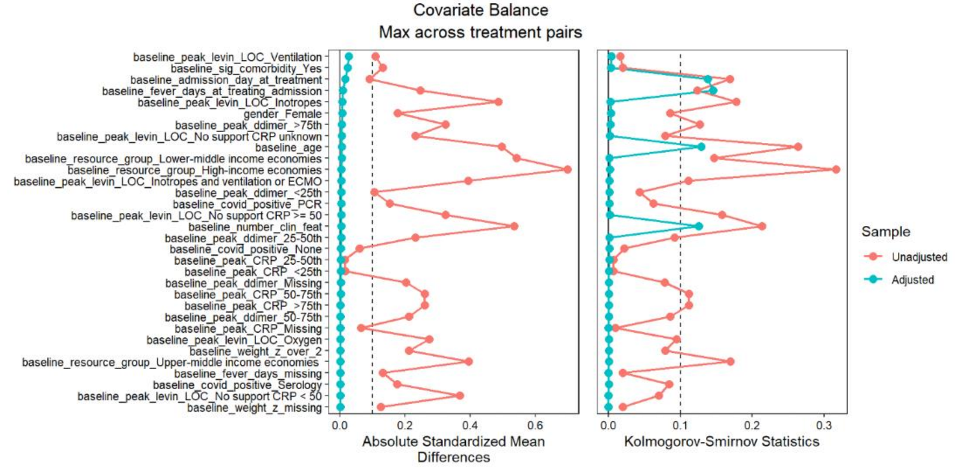

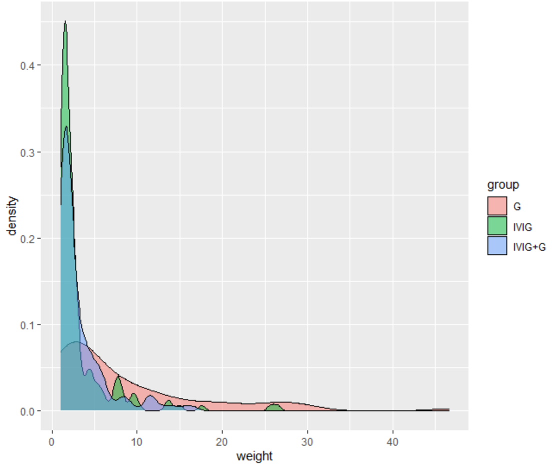

References

1. RCPCH. Paediatric multisystem inflammatory syndrome temporally associated with COVID-19 (PIMS) Available from: https://www.rcpch.ac.uk/resources/paediatric-multisystem-inflammatory-syndrome-temporally-associated-covid-19-pims-guidance
2. CDC C for DC and P. Information for Healthcare Providers about Multisystem Inflammatory Syndrome in Children (MIS-C) Available from: https://www.cdc.gov/mis-c/hcp/
3. WHO. Multisystem inflammatory syndrome in children and adolescents temporally associated with COVID-19 Available from: https://www.who.int/publications/i/item/multisystem-inflammatory-syndrome-in-children-and-adolescents-with-covid-19
4. RCPCH growth app Available from: https://www.rcpch.ac.uk/resources/growth-charts
5. Lopez L, Colan SD, Stylianou Mario, et al. Relationship of Echocardiographic Z-Scores Adjusted for Body Surface Area to Age, Sex, Race, and Ethnicity: The Pediatric Heart Network Normal Echocardiogram Database. 2017;10(11).
6. Schemper, M., Wakounig, S. & Heinze, G. The estimation of average hazard ratios by weighted Cox regression. Stat. Med. 28, 2473–2489 (2009).
7. VanderWeele, T. J. & Ding, P. Sensitivity Analysis in Observational Research: Introducing the E-Value. Ann. Intern. Med. 167, 268–274 (2017).
8. McCrindle BW, Rowley AH, Newburger JW, et al. Diagnosis, Treatment, and Long-Term Management of Kawasaki Disease: A Scientific Statement for Health Professionals From the American Heart Association. Circulation. 2017;135(17):e927–99.
